# Molecular Engineering and Morphology Control of Covalent Organic Frameworks for Enhancing Activity of Metal‐Enzyme Cascade Catalysis

**DOI:** 10.1002/advs.202400730

**Published:** 2024-04-23

**Authors:** Hao Zhao, Jialin Zhang, Yunting Liu, Xinlong Liu, Li Ma, Liya Zhou, Jing Gao, Guanhua Liu, Xiaoyang Yue, Yanjun Jiang

**Affiliations:** ^1^ National‐Local Joint Engineering Laboratory for Energy Conservation in Chemical Process Integration and Resources Utilization School of Chemical Engineering and Technology Hebei University of Technology Tianjin 300401 China

**Keywords:** covalent organic framework, dynamic kinetic resolution, hollow structure, metal‐enzyme integrated catalysts, microenvironment modulation

## Abstract

Metal‐enzyme integrated catalysts (MEICs) that combine metal and enzyme offer great potential for sustainable chemoenzymatic cascade catalysis. However, rational design and construction of optimal microenvironments and accessible active sites for metal and enzyme in individual nanostructures are necessary but still challenging. Herein, Pd nanoparticles (NPs) and *Candida antarctica* lipase B (CALB) are co‐immobilized into the pores and surfaces of covalent organic frameworks (COFs) with tunable functional groups, affording Pd/COF‐X/CALB (X = ONa, OH, OMe) MEICs. This strategy can regulate the microenvironment around Pd NPs and CALB, and their interactions with substrates. As a result, the activity of the COF‐based MEICs in catalyzing dynamic kinetic resolution of primary amines is enhanced and followed COF‐OMe > COF‐OH > COF‐ONa. The experimental and simulation results demonstrated that functional groups of COFs modulated the conformation of CALB, the electronic states of Pd NPs, and the affinity of the integrated catalysts to the substrate, which contributed to the improvement of the catalytic activity of MEICs. Further, the MEICs are prepared using COF with hollow structure as support material, which increased accessible active sites and mass transfer efficiency, thus improving catalytic performance. This work provides a blueprint for rational design and preparation of highly active MEICs.

## Graphical abstract

Pd NPs and CALB are space‐separately co‐immobilized into the pores and surfaces of covalent organic frameworks with tunable functional groups to prepare metal‐enzyme integrated catalysts. The microenvironment is regulated by molecular engineering and morphology control to improve the dynamic kinetic resolution activity of metal‐enzyme cascade catalyzed primary amine.

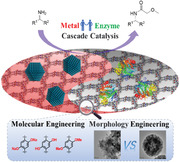

## 1 Introduction

The one‐pot cascade catalysis combined with chemical catalysis and biocatalysis provides an elegant process route for asymmetric synthesis, which inherits the advantages of strong productivity of chemical catalysts and high chemo‐, regio‐, and stereo‐selectivity of enzyme catalysts.^[^ ^1^ ^]^ Besides, the separation and accumulation of intermediate products can be avoided, and the synthesis efficiency of cascade reaction can be improved. However, in a typical example, the stability, compatibility (toxicity of metals to enzymes, incompatibility of reaction conditions) and reusability of free metal nanoparticles (NPs) and enzymes limit the application of metal‐enzyme cascade catalysis.^[^ ^2^ ^]^ The preparation of heterogeneous metal‐enzyme integrated catalysts (MEICs) by co‐immobilization of two catalysts to different compartments of the same support is an effective strategy to solve the above problems, and can shorten the space distance between the two catalysts to enhance synergistic effects.^[^ ^1^, ^3^ ^]^ So far, several porous materials (e.g., metal−organic framework, C_3_N_4_, mesoporous silica, etc.) have been used as carriers to prepare MEICs to improve the stability and compatibility of the two catalysts and realize the recycling in one‐pot cascade catalysis.^[^ ^2^, ^4^ ^]^ Nevertheless, the low activity of immobilized metal NPs and enzymes due to the lack of microenvironmental modulation and accessible active sites remains a significant barrier to the application of MEICs in cascade reactions. Therefore, the development of a suitable platform to regulate the local microenvironment around the catalysts and provide unimpeded guest molecular transfer channels is crucial for the construction of MEICs with high activity.

Covalent organic frameworks (COFs) are periodic porous polymer materials with high stability, high crystallinity, and structural tailorability formed from multiple organic monomers linked by reversible covalent bonds.^[^ ^5^ ^]^ The adjustable pore structure and customizable function allow metal NPs and enzyme to be space‐separately co‐immobilized to the pore and surface of COFs through the pore sieving effect, which enhances the catalyst stability.^[^ ^6^ ^]^ More importantly, the designable and easily modifiable functional groups of COFs can regulate the local microenvironment around the catalysts offering specific interactions between COFs and catalysts, and then regulate the properties of catalysts (such as the electronic properties of metal, the conformation of enzyme) providing the possibility to improve the activity of the MEICs.^[^ ^7^ ^]^ Recent studies have focused on the molecular engineering of COFs to regulate the microenvironment of a single metal or enzyme catalyst, but the simultaneous regulation of both has not been reported. Besides, most heterogeneous catalysts prepared with COFs as carrier face the following problems: (i) The poor dispersion of COF/metal NPs or COF/enzyme in the reaction system leads to poor accessibility of the substrate; (ii) Metal NPs or enzyme loaded on COFs surface can block pores and affect the accessible active sites. Hollow structure with through channel offers a solution to these challenges. The presence of a central cavity reduces the density of the catalyst, making it easier to disperse uniformly in the solution. The through channel and pore structure allow for simultaneous transfer of guest molecules from the inner and outer surfaces of the shell, enhancing mass transfer efficiency.^[^ ^8^ ^]^

Palladium nanoparticles (Pd NPs)^[^ ^9^ ^]^ and lipase are commonly used catalysts in chemical catalysis and biocatalysis, respectively.^[^ ^10^ ^]^ In this study, a typical example of metal‐enzyme cascade catalysis is chosen as a reaction model (Figure S1, Supporting Information), that is Pd NPs and *Candida antarctica* lipase B (CALB) catalyzed dynamic kinetic resolution (DKR) to obtain optically pure chiral amines. First, COF‐X with different groups (X = ONa, OH, and OMe) were prepared by molecular engineering, and then Pd NPs and CALB were co‐immobilized on the pore and surface of COF by encapsulation and surface modification, respectively, for the preparation of Pd/COF‐X/CALB MEICs. The hydrophobic microenvironment provided by COF‐OMe improves the specific affinity of the Pd/COF‐OMe/CALB to hydrophobic substrate, which enhances the catalytic activity. The Pd/COF‐OMe/CALB showed the highest activity in Pd/COF‐X/CALB. Mechanism studies have shown that the change of enzyme conformation, could improve the accessibility of the catalytic site of CALB. In addition, different groups in the pore wall of COF can regulate the electronic state of Pd NPs. The electron density of Pd NPs in Pd/COF‐OMe calculated by density functional theory (DFT) was the highest, which improves the racemization activity of primary amines. Thus the simultaneous microenvironment regulation of both metal and enzyme catalysts was achieved for the first time. Further, the MEICs were prepared by using COF‐OMe with hollow structure (HCOF‐OMe) as support material through morphology control, which enhanced accessible active sites and mass transfer efficiency, thus improving catalytic performance.

## 2 Results and Discussion

**Figure 1:**
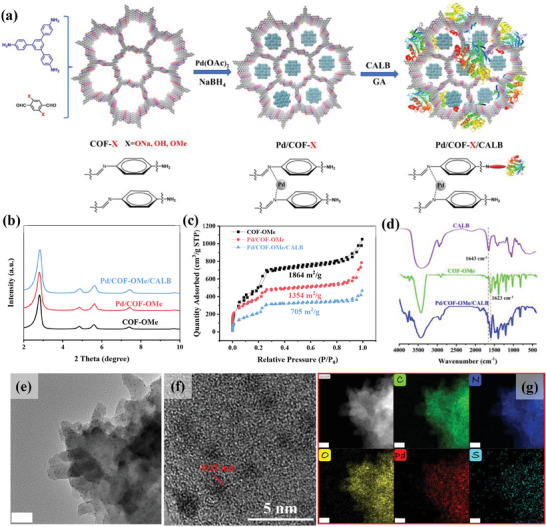
a) Synthetic routes of Pd/COF‐X/CALB. b) Small‐angle PXRD patterns and c) N_2_ adsorption‐desorption curves of COF‐OMe, Pd/COF‐OMe, and Pd/COF‐OMe/CALB. d) FT‐IR spectra of CALB, COF‐OMe, and Pd/COF‐OMe/CALB. e) TEM image, f) HR‐TEM image, and g) EDX elemental mapping of Pd/COF‐OMe/CALB (scale: 50 nm).

To carry out this study, we developed MEICs based on COF, and the preparation process is shown in **Figure** **1**a. Specifically, an imine‐linked COF (COF‐OMe) with hydrophobicity, high stability and 1D hexagonal mesoporous channels was synthesized from 2,5‐imethoxyterephthalaldehyde (DMTA) and 1,3,5‐tris(4‐aminophenyl)‐benzene (TAPB) by Schiff base reaction,^[^ ^11^ ^]^ which served as prototype carrier to co‐immobilize Pd NPs and CALB. Then the precursor Pd^2+^ was immobilized into the pore of COF‐OMe via ultrasound‐assisted double‐solvent approach, and further reduced by NaBH_4_ to form Pd NPs in Pd/COF‐OMe. The imide groups of COF‐OMe can provide nucleation sites for Pd NPs, and play the role of anchoring and stabilizing Pd NPs.^[^ ^12^ ^]^ Finally, Pd/COF‐OMe was treated with glutaraldehyde (GA), and CALB was immobilized on the surface of Pd/COF‐OMe to obtain Pd/COF‐OMe/CALB.^[^ ^13^ ^]^ The preliminary experiments show that CALB catalysis was the rate‐limiting step for Pd/COF‐OMe/CALB catalyzed DKR reaction. Therefore, the enzyme immobilized conditions (GA concentration and initial enzyme concentration) were optimized to increase the enzyme catalytic efficiency (Figures S2 and S3, Supporting Information). When GA concentration was 3% and initial enzyme concentration was 0.5 mg mL^−1^, the relative enzyme activity of Pd/COF‐OMe/CALB was the highest, the maximum CALB loading amount by Pd/COF‐OMe was 79.8 mg g^−1^_support_.

The crystallinity of COF‐OMe was characterized by powder X‐ray diffraction (PXRD), which showed characteristic peaks at 2*θ* = 2.81°, 4.85°, 5.59°, 7.41°, and 9.70°, corresponding to the reflections from the (100), (110), (200), (210), and (220) facets, respectively (Figure 1b). The experimental results were in good agreement with the eclipsed AA stacking model of the simulated COF‐OMe structure (Figure S4, Supporting Information). The characteristic peaks of Pd/COF‐OMe and Pd/COF‐OMe/CALB after loading Pd NPs and CALB had no significant change compared with that of COF‐OMe, indicating that the crystal structure of COF was completely reserved. It is worth noting that no characteristic peak of Pd NPs was observed in the PXRD of Pd/COF‐OMe and Pd/COF‐OMe/CALB (Figure S5, Supporting Information), which was caused by the small particle size and well dispersion of Pd.^[^ ^14^ ^]^ The N_2_ adsorption‐desorption characterization showed that COF‐OMe, Pd/COF‐OMe, and Pd/COF‐OMe/CALB were all type IV isotherm with Brunauer‐Emmett‐Teller (BET) surface area of 1864, 1354, and 705 m^2^ g^−1^, respectively (Figure 1c). The decrease of BET surface area after immobilization of Pd NPs and CALB is related to the occupation and blockage of pore channels by Pd and CALB.^[^ ^15^ ^]^ Moreover, as shown in Figure S6 (Supporting Information), the pore size of COF ranged from 2.8–3.5 nm, which is larger than the particle size of Pd NPs (2–3 nm) (Figure S7, Supporting Information), allowing the immobilization of Pd NPs in the pores. The average pore size also decreased successively, which proved that Pd NPs and CALB were successfully immobilized. It is worth noting that the particle size of CALB (3 × 4 × 5 nm) is larger than the pore size of COF‐OMe (Figure S6, Supporting Information),^[^ ^16^ ^]^ so Pd NPs and CALB achieve space‐separated co‐immobilization through pore sieving effect to avoid mutual interference of catalysts. As shown in Figure S8 (Supporting Information), the Fourier transform infrared (FT‐IR) spectra of COF‐OMe showed the disappearance of the peaks corresponding to the amino group in TAPB and the aldehyde group in DMTA, which are at 3300 cm^−1^ and 1670 cm^−1^, respectively. Meanwhile, the vibration peak at 1623 cm^−1^ also indicated the formation of C = N bond via Schiff base reaction between the aldehyde group of DMTA and the amino group of TAPB. All these results indicated the successful synthesis of COF‐OMe.^[^ ^17^ ^]^ FT‐IR spectra showed not only the retention of the stretching peak of C = N bond at 1623 cm^−1^ in Pd/COF‐OMe/CALB compared to COF‐OMe, but also the appearance of a new peak at 1643 cm^−1^ attributed to the stretching vibrational peak of amide I of CALB, indicating the successful immobilization of CALB (Figure 1d).^[^ ^17^, ^18^ ^]^ The scanning electron microscopy (SEM) showed that Pd/COF‐OMe/CALB was an irregular block stacked structure (Figure S9, Supporting Information). The transmission electron microscopy (TEM) and high‐angle annular dark field (HAADF) images showed that Pd NPs in Pd/COF‐OMe/CALB were mainly 2–3 nm and well dispersed, which was attributed to the COF pore‐limiting effect that inhibited the growth of Pd NPs to large particle size and agglomeration (Figure 1e–g; Figure S7, Supporting Information).^[^ ^14^, ^19^ ^]^ It can be seen in high‐resolution TEM (HR‐TEM) image that the lattice space of Pd was 0.22 nm, which corresponded to the (111) plane of Pd (Figure 1f).^[^ ^20^ ^]^ TEM‐mapping showed that C, N, O, Pd, and S elements in Pd/COF‐OMe/CALB were uniformly distributed (Figure 1g), where Pd element corresponded to Pd NPs, and S element was characteristic element of CALB proving that CALB was uniformly immobilized on COF. Fluorescein isothiocyanate (FITC) was used as a fluorescence probe to label CALB. Fluorescence microscopy images of Pd/COF‐OMe/FITC‐CALB showed that green fluorescence was distributed throughout the catalyst (Figure S10, Supporting Information), which demonstrated that Pd/COF‐OMe successfully immobilized CALB. The X‐ray photoelectron spectrometry (XPS) showed that the surface of COF‐OMe contained C, N, and O elements (Figure S11, Supporting Information). Compared with COF‐OMe, Pd element appeared on the surface of Pd/COF‐OMe and Pd/COF‐OMe/CALB, indicating that Pd atom was successfully introduced. The appearance of S element on the surface of Pd/COF‐OMe/CALB indicated the successful immobilization of CALB. Besides, no other elements were detected, indicating no other impurities were present. The high‐resolution spectra of element C showed that the proportion of chemical bonds did not change much except that the proportion in Pd/COF‐OMe/CALB increased at 287.75 eV (C═N), which was mainly formed by Schiff base reaction between CALB and GA (Figure S12, Supporting Information). Figure S13 (Supporting Information) shows the high‐resolution spectra of element N. Compared with COF‐OMe, Pd/COF‐OMe not only showed the presence of C═N and C─N bonds at 398.7 eV and 400.71 eV, respectively, but also showed the coordination bond between Pd and N at 399.31 eV, mainly because the imine group of COF can provide nucleation sites for Pd, playing a role in anchoring Pd and on COF‐OMe. The reducible functional groups of Pd/COF‐OMe are similar to those of COF‐OMe, indicating the stability of these groups in COF‐OMe during the reduction process. The proportion of N‐H bonds in Pd/COF‐OMe/CALB was significantly increased, which was contributed by the amino group in the CALB. According to XPS results, the Pd NPs and CALB were successfully immobilized on COF‐OMe through coordination and covalent interaction.

In order to better understand the influence of catalyst microenvironment on the activity of the metal‐enzyme cascades, COF‐OH and COF‐ONa were further prepared with similar topological structure but different hydrophilicity compared to COF‐OMe. The Pd/COF‐OH/CALB and Pd/COF‐ONa/CALB were prepared by similar method to Pd/COF‐OMe/CALB, and their hydrophilicities follow the trend of Pd/COF‐ONa/CALB > Pd/COF‐OH/CALB > Pd/COF‐OMe/CALB (Figure S14, Supporting Information). The PXRD patterns showed that these COFs had high crystallinity and 1D hexagonal pores, and the crystal structure did not change significantly after loading Pd NPs and CALB (Figures S15 and S16, Supporting Information). N_2_ adsorption‐desorption characterization showed that COF‐OMe, COF‐OH, and COF‐ONa had similar BET surface area and pore distribution, making them ideal candidates to specifically study the effects of microenvironment (Figure S6 and Figures S17 and S18, Supporting Information). Since COF‐OMe, COF‐OH, and COF‐ONa have similar pore‐limiting effects, the particle size distribution of Pd NPs in Pd/COF‐ONa/CALB, Pd/COF‐OH/CALB and Pd/COF‐OMe/CALB was concentrated in 2–3 nm (Figure S7 and Figures S19 and S20, Supporting Information), and the Pd content measured by inductively coupled plasma (ICP) was 2.41 wt.%, 2.55 wt.%, and 2.70 wt.%, respectively. Fluorescence microscopy images of Pd/COF‐ONa/FITC‐CALB and Pd/COF‐OH/FITC‐CALB showed that green fluorescence was distributed throughout the COF carriers, which demonstrated that Pd/COF successfully immobilized CALB (Figures S21 and S22, Supporting Information). The CALB loading capacity of Pd/COF‐ONa and Pd/COF‐OH measured by the Bradford method was 77.1 mg g^−1^_support_ and 78.7 mg g^−1^_support_, respectively.

The catalytic activity of Pd/COF‐X/CALB catalyst was examined by one of the representative metal‐enzyme catalyzed reactions, i.e., the DKR of primary amines.^[^ ^4^, ^20^, ^21^ ^]^ The metal‐enzyme bicatalyst overcomes the limit of 50% maximum yield by single enzyme and ensures a maximum theoretical yield of 100%. In this reaction, selective acylation of one enantiomer of racemic amines is catalyzed by enzyme, while Pd NPs catalyze the racemization of the unreacted enantiomer in a one‐pot process (Figure S1, Supporting Information).

**Figure 2:**
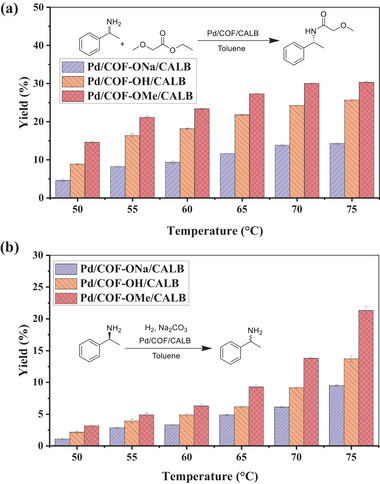
a) Effect of temperature and COF structure on the kinetic resolution of 1‐PEA catalyzed by Pd/COF‐X/CALB. Reaction conditions: 1‐PEA (0.25 mmol), ethyl methoxyacetate (0.5 mmol), toluene (2 mL), catalyst (3 mg, based on the CALB) and reaction time (2 h). b) Effect of temperature and COF structure on the racemization of (*S*)−1‐PEA catalyzed by Pd/COF‐X/CALB. Reaction conditions: (*S*)−1‐PEA (0.4 mmol), anhydrous sodium carbonate (20 mg), toluene (2 mL), catalyst (1 mg, based on the Pd) and reaction time (0.5 h).

First, the catalytic activity of CALB in Pd/COF‐X/CALB was evaluated by kinetic resolution of 1‐phenylethylamine (1‐PEA). The results showed that the activities of immobilized enzymes were all higher than those of free enzyme (**Figure** **2**a; Figure S23, Supporting Information). This was because free CALB molecules were easy to agglomerate in the organic phase, and the active site could not be fully utilized.^[^ ^8c^ ^]^ In contrast, the CALB immobilized in COF can effectively prevent it from agglomerating. For immobilized enzymes, the activity followed the trend of Pd/COF‐ONa/CALB < Pd/COF‐OH/CALB < Pd/COF‐OMe/CALB, which was the opposite of hydrophilicity (Figure 2a). On the one hand, the hydrophobic microenvironment of Pd/COF‐OMe/CALB can enrich organic substrates more effectively (Figure S24, Supporting Information), increase the local substrate concentration, and thus increase the reaction rate.^[^ ^22^ ^]^ On the other hand, the circular dichroism (Figure S25, Supporting Information) showed that the hydrophobic effect of COF‐OMe can cause conformational changes in CALB, inducing amino acid residues near the catalytic sites to be in an open state, thereby increasing the accessibility of the active sites.^[^ ^15^, ^21^, ^23^ ^]^

The effect of reaction temperature on the enzyme activity of Pd/COF‐X/CALB for the kinetic resolution of 1‐PEA was investigated. As shown in Figure 2a, the catalytic activity first increased with the increase of temperature to 70 °C, and then kept steady. However, high temperature could denaturate and deactivate enzyme molecules, affecting the activity of CALB. Therefore, the thermal stability of Pd/COF‐OMe/CALB and free CALB was investigated (Figure S26, Supporting Information). The results showed that 60%, 53%, and 36% of the initial enzyme activity were retained after incubation at 60, 70, and 80 °C for 72 h, respectively, indicating that the stability of Pd/COF‐OMe/CALB was worse under higher temperature, and the loss of activity was accelerated from 70 to 80 °C. Nevertheless, Pd/COF‐OMe/CALB showed great advantages over free enzyme, which retained only 17% of the initial activity after incubation at 60 °C for 72 h. The high thermal stability of CALB in Pd/COF‐OMe/CALB was attributed to the fact that CALB was immobilized on Pd/COF‐OMe by covalent bond, which increased the rigid structure of enzyme molecules and made the spatial structure more stable.^[^ ^13^ ^]^ Therefore, Pd/COF‐OMe/CALB can still maintain good catalytic activity at high temperature.

**Figure 3:**
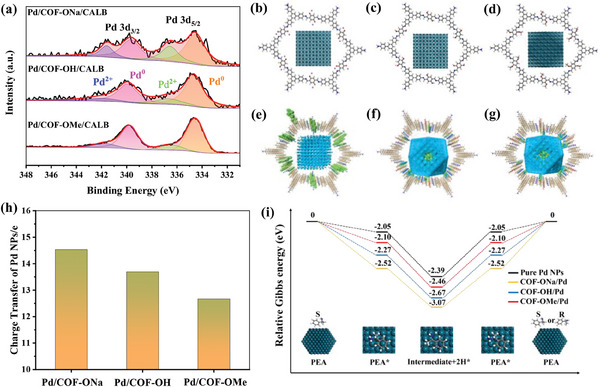
a) Pd 3d region in the XPS spectra of Pd/COF‐X/CALB. Top views of molecular models of b) Pd/COF‐ONa, c) Pd/COF‐OH, and d) Pd/COF‐Me. Electron density distribution profiles of e) Pd/COF‐ONa, f) Pd/COF‐OH, and g) Pd/COF‐OMe. (Blue represents a decrease in electron density and green represents an increase in electron density.) h) Calculated number of electron transfer from Pd NPs to the host COF‐X in Pd/COF‐X. i) Free‐energy profiles for the (*S*)−1‐PEA racemization on Pd/COF‐X and pure Pd NPs.

It can be observed that the catalytic activity of Pd/COF‐X/CALB for (*S*)−1‐phenethylamine raised with the increasing temperature (Figure 2). The activity did not show any inflection point from 50 °C to 75 °C. However, the biocatalytic step by CALB is the rate‐determining step for Pd/COF‐OMe/CALB catalyzed DKR reaction. Considering the compatibility with enzyme catalysis, and the long reaction time for the dynamic kinetic resolution, 70 °C was chosen as the reaction temperature for subsequent study. The catalytic activity of Pd NPs in Pd/COF‐X/CALB was evaluated by the racemization of (*S*)−1‐PEA (Figure 2b). The yields of racemic 1‐PEA by Pd/COF‐ONa/CALB, Pd/COF‐OH/CALB, and Pd/COF‐OMe/CALB were 6%, 9%, and 14%, respectively. The activity also followed the trend of Pd/COF‐ONa/CALB < Pd/COF‐OH/CALB < Pd/COF‐OMe/CALB, which was the opposite of hydrophilicity. Similarly, the hydrophobic microenvironment of Pd/COF‐OMe/CALB was conducive to enriching organic substrates, which was one of the reasons for improving the catalytic activity of Pd NPs. In addition, different supports also affect the surficial electronic properties of Pd NPs, and then affect the activity of Pd NPs. We noticed that although Pd^0^ and Pd^2+^ presented in all catalysts, the proportion was different (**Figure** **3**a). The percentages of Pd^0^ in Pd/COF‐ONa/CALB, Pd/COF‐OH/CALB, and Pd/COF‐OMe/CALB were 69%, 79%, and 84%, respectively, which may be another reason for the difference in racemization activity of Pd NPs catalytic (*S*)−1‐PEA.

In order to have a deeper understanding of the reasons for the difference in Pd^0^ content in Pd/COF‐X/CALB and the difference in catalytic activity, the electronic states on the surface of Pd in Pd/COF‐ONa, Pd/COF‐OH and Pd/COF‐OMe and the Gibbs free energy change during the racemization of (*S*)−1‐PEA were calculated by DFT. According to TEM results, a model of Pd NPs consisting of 561 Pd atoms (particle size ≈3 nm) (Figure S27a, Supporting Information) and a model of three COF‐X immobilized Pd NPs (Figure 3b–d; Figure S27b–d, Supporting Information) were constructed. In order to reflect the holding and anchoring effect of COF‐X pores on Pd NPs, the constructed COF unit consists of 6 single‐layer COF fragments with AA stacking. DFT calculation showed that the surface electron density of Pd NPs decreased, while the electron density of COF‐X increased, indicating electrons transferred from Pd NPs to COF‐X (Figure 3e–g).^[^ ^24^ ^]^ The number of transferred electrons in Pd/COF‐ONa, Pd/COF‐OH, and Pd/COF‐OMe were further calculated as 14.53e, 13.69e, and 12.67e, respectively (Figure 3h). The larger number of transferred electrons meant that Pd NPs lost more electrons and Pd valence state rised more, which was consistent with XPS characterization. According to the number of electrons transferred from Pd NPs to COF‐X, Pd NPs with 14, 13, and 12 positive charges were constructed to represent the Pd/COF‐ONa, Pd/COF‐OH, and Pd/COF‐OMe catalysts, respectively. The Gibbs free energy changes during the racemization of (*S*)−1‐PEA catalyzed by Pd/COF‐OX were further studied by DFT calculation (Figure 3i). In the racemization process of (*S*)−1‐PEA catalyzed by Pd NPs, (*S*)−1‐PEA first adsorbed on Pd NPs, and then transformed into imine intermediates and finally into (*S*)−1‐PEA and (*R*)−1‐PEA. The calculation showed that the desorption energy (PEA*→PEA) of Pd/COF‐OMe was 0.42 eV and 0.17 eV lower than that of Pd/COF‐ONa and Pd/COF‐OH, respectively. And the dehydrogenation/hydrogenation energy (PEA*→intermediate→PEA*) of Pd/COF‐OMe was 0.19 eV and 0.04 eV lower than those of Pd/COF‐ONa and Pd/COF‐OH, respectively. The Pd/COF‐OMe with the least number of transferred electrons had the lowest hydrogenation and desorption energy in the racemization of (*S*)−1‐PEA.^[^ ^20^ ^]^ Therefore, the metal activity of Pd/COF‐OMe was higher than that of Pd/COF‐ONa and Pd/COF‐OH. More Pd^0^ contributed to the higher catalytic activity of Pd/COF‐OMe during catalyzing the racemization of (*S*)−1‐PEA.

After revealing the activity of CALB and Pd NPs of Pd/COF‐OX/CALB in kinetic resolution of 1‐PEA and racemization of (*S*)−1‐PEA, we further investigated the activity of metal‐enzyme cascade catalytic DKR. Considering the catalytic activity of metals and enzymes and the long‐term stability of enzymes under harsh reaction conditions, a reaction temperature of 70 °C has been chosen for the metal‐enzyme cascade catalyzed DKR. After comparing different catalysts (**Table** **1** and Table S1), Pd/COF‐OMe/CALB showed the best catalytic activity with the highest yield of 94% at 10 h, while Pd/COF‐ONa/CALB and Pd/COF‐OH/CALB were 58% and 76%, respectively. This is attributed to the fact that COF‐OMe provides a suitable microenvironment for Pd NPs and CALB and has a favorable impact: (i) Hydrophobic action causes the lid above the CALB active site to open, increasing the accessibility of the active site; (ii) Molecular engineering of COFs regulates the electronic state of Pd NPs, controls the interactions of Pd NPs with substrates, and improves the catalytic activity of Pd NPs; (iii) The hydrophobic microenvironment of Pd/COF‐OMe/CALB can enrich organic substrates, increase the local substrate concentration, and thus increase the reaction rate. In control experiments, the catalytic activity of the physical mixture of Pd/COF‐OMe and COF‐OMe/CALB was lower than that of the integrated Pd/COF‐OMe/CALB catalyst, indicating that the co‐immobilization of Pd NPs and CALB has synergistic effect (Table 1, Entry 2–3).

**Table 1:** Effect of different catalysts on DKR.

Entry^a)^	Catalyst	Time [h]	Yield^b)^ [%]	ee^b)^ [%]
1	Pd/COF‐OMe/CALB	10	94	> 99
2	Pd/COF‐OMe + COF‐OMe/CALB	10	83	> 99
3	Pd/COF‐OMe + COF‐OMe/CALB	12	91	> 99

^a)^ Reaction conditions: All reactions were carried out in dry toluene (2.0 mL) with 1‐PEA (0.25 mmol), ethyl methoxyacetate (0.5 mmol), catalyst (40 mg) and dry Na_2_CO_3_ (20 mg);

^b)^ Determined by gas chromatography and pentadecane as internal standard.

The space‐separated co‐immobilization of Pd NPs and CALB in Pd/COF‐X/CALB can synergistically enhance the cascaded catalytic performance. However, CALB loading on the surface of Pd/COF‐X will inevitably block part of the COF pores, resulting in dead‐end pores and inhibiting the activity of Pd NPs in the pores (Figure S28, Supporting Information). To solve this problem, we proposed to co‐immobilize Pd NPs and CALB to a hollow COF (HCOF‐OMe). The size of CALB is larger than the pore diameter of HCOF‐OMe, so the enzyme cannot be immobilized to the inner cavity surface of HCOF‐OMe. In this way, even if the outer surface is partially blocked, the substrate is still expected to contact with Pd NPs through the internal channels. Therefore, the effect of morphology structure of COF on the catalytic activity of MEICs was further studied.

**Figure 4:**
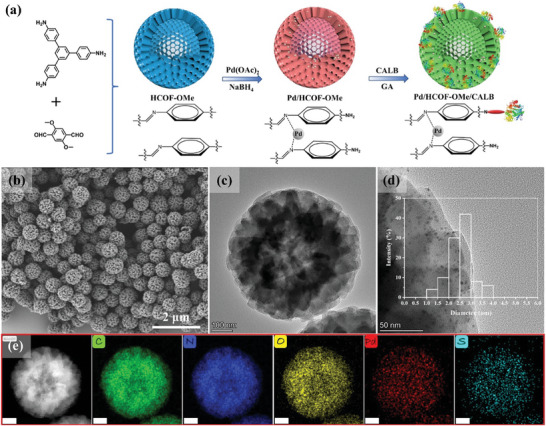
a) Synthetic route of Pd/HCOF‐OMe/CALB. b) SEM, c) TEM, and d) HR‐TEM (inset: size distribution of Pd NPs) images of Pd/HCOF‐OMe/CALB. e) EDX elemental mapping of Pd/HCOF‐OMe/CALB (scale: 100 nm).

First, according to previous reports,^[^ ^8c^ ^]^ hollow spherical COF (HCOF‐OMe) was synthesized, and the morphology was characterized by SEM and TEM (Figure S29, Supporting Information). SEM image showed that HCOF‐OMe was uniformly dispersed sphere with 500–600 nm diameter and rough flower‐like surface. The TEM image showed that the interior of HCOF‐OMe contained a cavity, and it was observed that the interior cavity was connected to the outside by a through channel which could provide the rapid transfer of substrate/product during catalysis.^[^ ^8c^ ^]^ Solid satate ^13^C NMR test results (Figure S30, Supporting Information) were consistent with previously reported results, indicating the successful preparation of HCOF‐OMe.^[^ ^8c^ ^]^ The Pd/HCOF‐OMe/CALB was prepared by similar method to Pd/COF‐OMe/CALB (**Figure** **4a**). The content of Pd measured by ICP was 2.77 wt.%, and the CALB loading capacity of Pd/HCOF‐OMe measured by the Bradford method was 89.2 mg g^−1^_support_. Compared with Pd/COF‐OMe/CALB, the loading capacity of Pd NPs was similar, while the enzyme loading was higher due to the numerous rod‐like structures on the surface of HCOF‐OMe (Figure 4b), which provided more anchoring sites for enzyme immobilization. The SEM and TEM images (Figure 4b,c) showed that the morphology and structure of Pd/HCOF‐OMe/CALB after loading Pd NPs and CALB had no obvious changes compared with HCOF‐OMe. The particle size distribution of Pd NPs in Pd/HCOF‐OMe/CALB was also concentrated in 2–3 nm (Figure 4d). TEM‐mapping showed the existence of Pd and S elements in Pd/HCOF‐OMe/CALB, which proved that Pd and enzyme have been successfully immobilized (Figure 4e). Fluorescence microscopy images further demonstrated the successful immobilization of CALB (Figure S31, Supporting Information).

**Figure 5:**
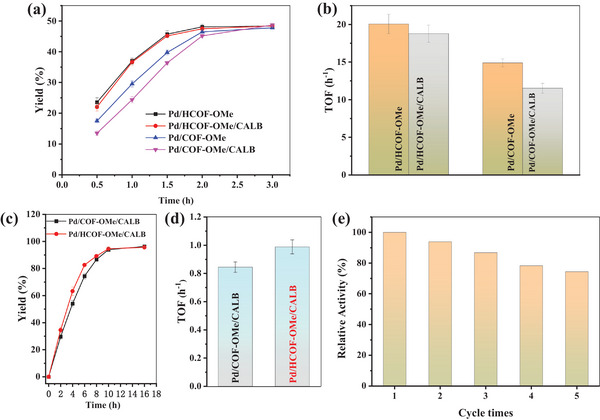
a) Reaction process and b) corresponding TOF of the racemization reaction toward (*S*)−1‐PEA catalyzed by Pd/COF‐OMe, Pd/COF‐OMe/CALB, Pd/HCOF‐OMe, and Pd/HCOF‐OMe/CALB. c) Reaction process and d) corresponding TOF of DKR of 1‐PEA catalyzed by Pd/HCOF‐OMe/CALB and Pd/COF‐OMe/CALB. e) Recyclability of Pd/HCOF‐OMe/CALB for DKR of 1‐PEA.

In order to investigate the effect of morphology and structure on the MEICs, the activity of Pd NPs before and after CALB immobilization on Pd/COF‐OMe and Pd/HCOF‐OMe was studied (**Figure** **5**a,b). With adding the same amount of Pd NPs, Pd/HCOF‐OMe had higher activity than Pd/COF‐OMe attributed to the special hollow structure of HCOF‐OMe which enhanced the diffusion of substrates and products. Figure 5b showed after the immobilization of CALB, the TOF of Pd NPs in Pd/HCOF‐OMe/CALB did not decrease significantly (6.4%), while that of Pd/COF‐OMe/CALB decreased by 22.7%. Surprisingly, the activity of Pd/HCOF‐OMe/CALB was still higher than that of Pd/COF‐OMe. This was because the immobilized enzyme on the surface of Pd/COF‐OMe caused pore blocking thus inhibiting mass transfer. Whereas Pd/HCOF‐OMe contained through channel and cavity, so even if the external surface was blocked, the substrate or product can still diffuse and bound to Pd NPs through the channel communicating with the internal cavity.^[^ ^8b^ ^]^

The number of accessible active sites of Pd NPs before and after loading CALB on Pd/HCOF‐OMe and Pd/COF‐OMe was further studied by CO titration experiments.^[^ ^22b^ ^]^ The molecular kinetic diameter of CO is 0.376 nm,^[^ ^25^ ^]^ and the layer spacing of COF‐OMe is 0.350 nm (Figure S4, Supporting Information). Therefore, CO can only bind to Pd NPs through 1D pore migration. The dispersion of Pd in Pd/HCOF‐OMe and Pd/HCOF‐OMe/CALB was 45.4% and 43.6%, respectively, with little change after CALB loading (Table S2, Supporting Information). The corresponding average diameter of Pd NPs was 2.47 nm and 2.56 nm, respectively, which was consistent with the characterization by TEM. However, Pd dispersion decreased from 43.8% to 33.6% after loading CALB on Pd/COF‐OMe. It was further indicated that CALB loading on Pd/COF‐OMe with irregular morphology was easy to block the pore of COF, which affected the accessibility of the active sites of Pd NPs. On the contrary, the effect of CALB on the activity of Pd NPs in the pore can be effectively avoided by using the hollow spherical HCOF‐OMe to co‐immobilize Pd NPs and CALB.

**Scheme 1:**
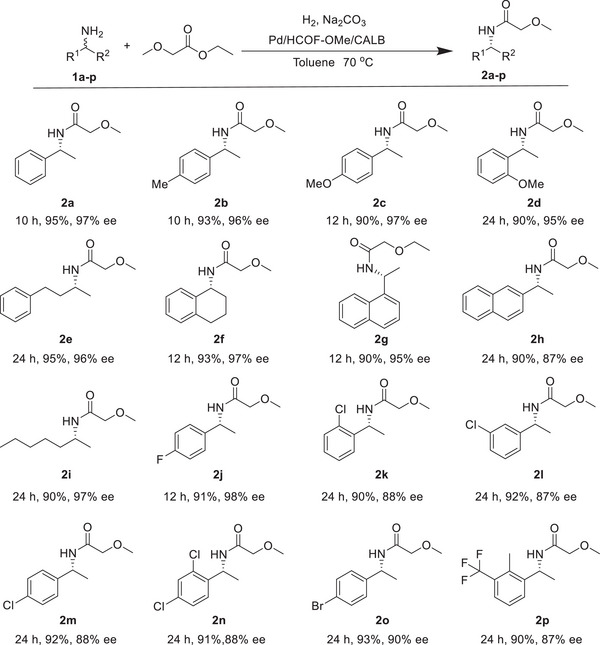
Chemoenzymatic DKR of primary amines.

The DKR of 1‐PEA was investigated by Pd/COF‐OMe/CALB and Pd/HCOF‐OMe/CALB in one‐pot cascade catalysis. Pd/HCOF‐OMe/CALB showed a higher initial reaction rate than Pd/COF‐OMe/CALB (Figure 5c). In addition, Pd/HCOF‐OMe/CALB had a 17% higher TOF than Pd/COF‐OMe/CALB (Figure 5d). Therefore, the catalytic activity of the MEICs can be improved by adjusting the morphology structure of the carrier. In order to understand the heterogeneous properties and stability of Pd/HCOF‐OMe/CALB, the filtration test was performed.^[^ ^24a^ ^]^ The catalyst was removed at 3 h of reaction and no product was produced (Figure S32, Supporting Information), reflecting the heterogeneous feature of the catalytic process with no catalyst leakage. The reusability of the Pd/HCOF‐OMe/CALB in one‐pot cascade DKR of 1‐PEA was further investigated. After 5 cycles, Pd/HCOF‐OMe/CALB retained 68% of the initial activity (Figure 5e) and the enantioselectivities (ee) remained >98% (Figure S33, Supporting Information), indicating that the prepared integrated catalyst had good reusability. The decreased activity of Pd/HCOF‐OMe/CALB after use may be caused by the long reaction time and high reaction temperature, which could damage the structure of enzyme molecules. The crystal structure and morphological structure of Pd/HCOF‐OMe/CALB did not change significantly after 5 cycles of use (Figures S34 and S35, Supporting Information). The high stability of the carriers provided an important guarantee for the stability of the integrated catalyst. The TEM image showed that Pd NPs were still uniformly distributed on the carrier (Figure S35b, Supporting Information) attributed to the pore sieving effect of COF, which could effectively avoid Pd NPs agglomeration. After 5 cycles, the content of Pd in the integrated catalyst was 2.67%, which had little change compared with the initial content (2.77%). This was because Pd NPs interacted with imines of the pores thus preventing the loss of Pd NPs.^[^ ^6^ ^]^ The FT‐IR spectra (Figure S36, Supporting Information), Solid state ^13^C NMR spectra (Figure S37, Supporting Information), and the XPS (Figure S38, Supporting Information) results of reused Pd/HCOF‐OMe/CALB showed no obvious changes after use, indicating its high stability. In addition, the immobilization of CALB on COF also enhanced the relatively stability and reusability of enzyme. The applicability of Pd/HCOF‐OMe/CALB to other primary amines with different substituents in DKR reaction was further investigated (**Scheme** **1**). It is delightful that all sixteen substrates showed high yields (90–95%) and ee value (87–99%). Based on the above results, the general applicability of Pd/HCOF‐OMe/CALB can be well fulfilled.

## 3 Conclusion

In summary, we proposed a strategy to prepare MEICs by using COFs with easily adjustable functional groups to space‐separately co‐immobilize Pd NPs and CALB, which provided the compatibility and stability of the metal and enzyme in the application of one‐pot cascade catalytic DKR of primary amines. More importantly, the activity of the MEICs can be improved through the molecular engineering and morphology control of COFs. As a result, the activity of the Pd‐CALB integrated catalyst in catalyzing DKR of primary amines follows Pd/COF‐OMe/CALB > Pd/COF‐OH/CALB > Pd/COF‐ONa/CALB. The circular dichroism indicated that the hydrophobic effect of COF‐OMe changed the secondary structure of CALB, exposed the active sites, and then increased the catalytic activity of the enzyme. The XPS and DFT calculations show that the molecular engineering of COFs regulates the electronic state of Pd NPs, controls the interactions of Pd NPs with substrates, and improves the catalytic activity of Pd NPs. In addition, the hydrophobic COF‐OMe also facilitates the substrate enrichment of the integrated catalyst and further improves the activity. Furthermore, the MEICs were prepared by using HCOF‐OMe with hollow structure as carrier material, which enhances accessible active sites and improves mass transfer efficiency, thus improving catalytic performance. This work provides rational molecular engineering and morphology control for carrier materials to provide a new route for the preparation of high‐performance cascade catalysts.

## 4 Experimental Section

### Chemicals and Characterization

Details in supporting materials.

### Synthesis of COF‐OMe

**Scheme 2:**
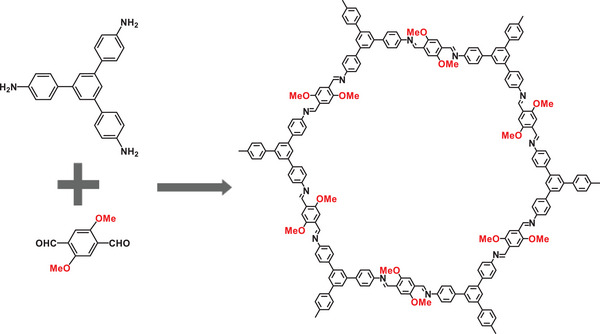
Synthetic routine for COF‐OMe.

COF‐OMe was prepared according to the method in reference with minor modification.^[^ ^11^ ^]^ First, TAPB (0.080 mmol), DMTA (0.12 mmol), 1,2‐dichlorobenzene (1 mL), *n*‐butylalcohol (1 mL), and acetic acid (6 M, 0.2 mL) were added to the reaction tube with side neck and degassed via three freeze–pump–thaw cycles. Then, the mixture was reacted in an oil bath at 120 °C for 3 days. When the reaction was completed, the resulting precipitate was washed six times with tetrahydrofuran. Then, the obtained precipitate was purified with a Soxhlet extractor containing tetrahydrofuran for 24 h. Finally, the product named COF‐OMe was dried under vacuum at 60 °C for 12 h for further use (**Scheme** 2).

### Synthesis of HCOF‐OMe

TAPB (1.0 mmol), DMTA (1.5 mmol), and acetonitrile (125 mL) were added into a round‐bottom flask and mixed by sonication. Then, the acetic acid (7.5 mL, 12 M) was added to the round‐bottom flask and stirred vigorously. After a yellow precipitate appeared, the stirring was stopped, and the reaction was allowed to stand at room temperature for 5 days. The yellow precipitate was separated by centrifugation and washed with tetrahydrofuran and ethanol. The product was dried under vacuum at 60 °C for 12 h.

### Synthesis of Pd/COF‐X

First, 100 mg of COF‐OMe was added to 30 mL of dichloromethane solution containing 7.8 mg palladium acetate, and the mixed solution was ultrasonically treated for 24 h. Then the precipitation was centrifuged and cleaned with methylene chloride several times. The precipitation was collected and dried under vacuum at 60 °C for 6 h. The metal precursor‐loaded COF‐OMe is added to a round‐bottomed flask containing absolute ethanol (30 mL) and ultrasonically dispersed. Then, newly prepared NaHB_4_ aqueous solution (0.53 M, 1 mL) was added under agitation to reduce metal precursor, and the reaction was carried out at room temperature for 4 h. The product was separated by filtration, washed with ethanol and water, and dried under vacuum at 60 °C for 12 h to obtain Pd/COF‐OMe.

Pd/COF‐OH, Pd/COF‐ONa, and Pd/HCOF‐OMe were prepared by the similar method as Pd/COF‐OMe.

### Synthesis of Pd/COF‐X/CALB

First, 50 mg of Pd/COF‐OMe was added into 20 mL of glutaraldehyde solution with a certain concentration (0–9 wt.%) for 4 h. After the reaction was completed, the precipitation was separated by filtration and cleaned with pure water for three times. Further, 10 mg of glutaraldehyde modified Pd/COF‐OMe was dispersed into 3 mL of PBS (100 mM, pH = 7) with a certain concentration of CALB (0.1–0.75 mg mL^−1^), and then the mixture was placed on a shaking table at 25 °C with stirring (170 r min^−1^) for 2 h. After centrifugal separation, the obtained precipitate was washed 3 times with ultrapure water. The obtained Pd/COF‐OMe/CALB was freeze‐dried for 12 h, and the loading amount of the enzyme was determined by the Bradford method.

Pd/COF‐OH/CALB, Pd/COF‐ONa/CALB, and Pd/HCOF‐OMe/CALB were prepared by the similar method as Pd/COF‐OMe/CALB.

## Conflict of Interest

The authors declare no conflict of interest.

## Supporting information

Supporting Information

## Data Availability

The data that support the findings of this study are available from the corresponding author upon reasonable request.
