# Supplementary material for: Molecular Engineering and Morphology Control of Covalent Organic Frameworks for Enhancing Activity of Metal‐Enzyme Cascade Catalysis
Source: Adv Sci (Weinh). 2024 Apr 23;11(25):2400730. doi: 10.1002/advs.202400730 (PMC11220694; doi:10.1002/advs.202400730)
Supplement: Supplementary file 1 — Supporting Information [file ADVS-11-2400730-s001.pdf]

ADVANCED SCIENCE
Open Access

Supporting Information

for *Adv. Sci.*, DOI 10.1002/advs.202400730

Molecular Engineering and Morphology Control of Covalent Organic Frameworks for Enhancing Activity of Metal-Enzyme Cascade Catalysis

Hao Zhao, Jialin Zhang, Yunting Liu, Xinlong Liu, Li Ma, Liya Zhou, Jing Gao, Guanhua Liu, Xiaoyang Yue* and Yanjun Jiang**

Supporting Information

Molecular engineering and morphology control of covalent organic frameworks for enhancing activity of metal-enzyme cascade catalysis

Hao Zhao [1], Jialin Zhang [1], Yunting Liu, Xinlong Liu, Li Ma, Liya Zhou, Jing Gao, Guanhua Liu *, Xiaoyang Yue * and Yanjun Jiang *

[1] These authors contributed equally.

H. Zhao; J. Zhang; Y. Liu; X. Liu; L. Ma; L. Zhou; J. Gao; G. Liu; X. Yue; Y. Jiang
National-Local Joint Engineering Laboratory for Energy Conservation in Chemical Process Integration and Resources Utilization, School of Chemical Engineering and Technology, Hebei University of Technology, Tianjin 300401, China
E-mail: ghliu@hebut.edu.cn; xiaoyang.yue@hebut.edu.cn; yanjunjiang@hebut.edu.cn

Table of Contents

1. Materials and Instruments. 5

1.1 Chemicals and materials. 5

1.2 Characterization. 5

2. Experimental Section. 6

2.1 Synthesis of COF-OH 6

2.2 Synthesis of COF-ONa 6

2.3 Synthesis of racemic amides 7

2.4 Racemization of (*S*)-1-PEA...7

2.5 Kinetic resolution of 1-PEA 8

2.6 Dynamic Kinetic Resolution of Primary Amines...8

2.7 The thermal stability of free CALB and Pd/COF-OMe/CALB 9

2.8 1-PEA adsorption studies 9

2.9 Reusability of Pd/HCOF-OMe/CALB 9

2.10 Calculation method 10

3. Figures and Tables..11

Figure S1. Schematic representation of dynamic kinetic resolution of chiral amines with esters as resolving agents..11

Figure S2. Standard curve of BSA concentration. 12

Figure S3. Influence of (a) GA concentration and (b) initial CALB concentration on relative activity of Pd/COF-OMe/CALB..13

Figure S4. (a) PXRD patterns of COF-OMe: experimental patterns (red) and the simulated pattern for eclipsed AA stacking mode (black); (b) Graphic view of COF-OMe AA stacking mode (The layer spacing is 0.35 nm)..14

Figure S5. Wide-angle PXRD patterns of COF-OMe, Pd/COF-OMe and Pd/COF-OMe/CALB. 15

Figure S6. Pore size distributions of COF-OMe, Pd/COF-OMe and Pd/COF-OMe/CALB. 16

Figure S7. Particle size profile of Pd NPs in Pd/COF-OMe/CALB. 17

Figure S8. FT-IR spectra of TAPB, DMTP and COF-OMe 18

Figure S9. SEM image of Pd/COF-OMe/CALB. 19

Figure S10. CLSM images of Pd/COF-OMe/FITC-CALB in (a) optical and (b) fluorescence. 20

Figure S11. XPS survey spectra of COF-OMe, Pd/COF-OMe and Pd/COF-OMe/CALB. ..21

Figure S12. C 1s regions in the XPS spectra of (a) COF-OMe, (b) Pd/COF-OMe and (c) Pd/COF-OMe/CALB...22

Figure S13. N 1s regions in the XPS spectra of (a) COF-OMe, (b) Pd/COF-OMe and (c) Pd/COF-OMe/CALB...23

Figure S14. The water contact angles of (a) Pd/COF-ONa/CALB, (b) Pd/COF-OH/CALB and (c) Pd/COF-OMe/CALB..24

Figure S15. (a) Small-angle XRD and (b) wide-angle XRD patterns of COF-OH, Pd/COF-OH and Pd/COF-OH/CALB...25

Figure S16. (a) Small-angle XRD and (b) wide-angle XRD patterns of COF-ONa, Pd/COF-ONa and Pd/COF-ONa/CALB. 26

Figure S17. (a) Nitrogen sorption isotherm curve and (b) pore-size distribution of COF-OH, Pd/COF-OH and Pd/COF-OH/CALB. 27
Figure S18. (a) Nitrogen sorption isotherm curve and (b) pore-size distribution of COF-ONa, Pd/COF-ONa and Pd/COF-ONa/CALB. 28
Figure S19. (a) TEM image, (b) HAADF image, (c) size distribution of Pd NPs and (d) EDX elemental mapping of Pd/COF-OH/CALB (scale: 50 nm). 29
Figure S20. (a) TEM image, (b) HAADF image, (c) size distribution of Pd NPs and (d) EDX elemental mapping of Pd/COF-ONa/CALB (scale: 50 nm). 30
Figure S21. CLSM images of Pd/COF-OH/FITC-CALB in (a) optical and (b) fluorescence. 31
Figure S22. CLSM images of Pd/COF-Na/FITC-CALB in (a) optical and (b) fluorescence. 32
Figure S23. The kinetic resolution of 1-phenethylamine catalyzed by free CALB. Reaction conditions: 1-PEA (0.25 mmol), ethyl methoxyacetate (0.5 mmol), toluene (2 mL) and CALB (3 mg). 33
Figure S24. Kinetics of 1-phenethylamine adsorption by Pd/COF-ONa/CALB, Pd/COF-OH/CALB and Pd/COF-OMe/CALB. 34
Figure S25. (a) Hydrophobic amino acids in α-helix 5; Representation of CALB at (b) closed and (c) open conformations; (d) CD spectra of COF-X/CALB and free CALB. 35
Figure S26. Thermal stability of free CALB and Pd/COF-OMe/CALB in toluene. 36
Figure S27. (a) Model of Pd NPs; Lateral molecular models of (b) Pd/COF-ONa, (c) Pd/COF-OH and (d) Pd/COF-OMe. 37
Figure S28. Schematic diagram of mass transfer of substrate molecules in (a) Pd/COF-OMe/CALB and (b) Pd/HCOF-Me/CALB catalytic system. 38
Figure S29. (a) SEM and (b) TEM images of HCOF-OMe. 39
Figure S30. The ^{13}C CP-MAS NMR spectrum of HCOF-OMe 40
Figure S31. CLSM images of Pd/HCOF-OMe/FITC-CALB in (a) optical and (b) fluorescence. 41
Figure S32. Filtration test of Pd/HCOF-OMe/CALB for the reaction. 42
Figure S33. Recyclability of Pd/HCOF-OMe/CALB for DKR of 1-PEA. 43
Figure S34. (a) Small-angle and (b) wide-angle PXRD patterns of Pd/HCOF-OMe/CALB after 5 times of cascade reaction. 44
Figure S35. (a) SEM and (b) TEM images of Pd/HCOF-OMe/CALB after 5 times of cascade reaction. 45
Figure S36. FT-IR spectra of Pd/HCOF-OMe/CALB before and after cascade reaction. 46
Figure S37. The ^{13}C CP-MAS NMR spectra of Pd/HCOF-OMe/CALB before and after cascade reaction. 47
Figure S38. XPS of Pd/HCOF-OMe/CALB before and after cascade reaction: (a) wide scan spectrum and (b) high-resolution spectra of Pd 3d. 48
Table S1. Effect of different catalysts on DKR[a]. 49
Table S2. CO chemisorption measurements at 293K over Pd/HCOF-OMe, Pd/HCOF-OMe/CALB, Pd/COF-OMe and Pd/COF-OMe/CALB 50
4. ^{1}H NMR spectra of the products recorded in $CDCl_3$. 51
5. GC and HPLC traces for productions 55

6. ^{1}H NMR spectra of compounds 71
7. Reference 79

1. Materials and Instruments.

1.1 Chemicals and materials.

1,3,5-tri-(4-aminophenyl)benzene (TAPB), 2,5-dimethoxyterephthalaldehyde (DMTA) and 2,5-dihydroxyterephthaldeyde (DHTP) were purchased from Jilin Chinese Academy of Sciences-Yanshen Technology Co., Ltd (Jilin, China). *Candida antarctica lipase* B (CALB) was purchased from Gaoruisen Technology Co., Ltd. (Beijing, China). Palladium acetate, 1,2-dichlorobenzene, *n*-butylalcohol, acetic acid, tetrahydrofuran (THF), NaOH, acetonitrile, glutaraldehyde (GA), chloroauric acid fluorescein isothiocyanate (FITC), 1-phenylethylamine (1-PEA) were purchased from Aladdin Industrial Corporation, (Shanghai, China). All chemicals were commercial and used without further purification.

1.2 Characterization.

Powder X-ray diffraction (PXRD) patterns were recorded using a Bruker AXS D8 Discover X-ray diffractometer with a Cu Kα anode (λ=0.15406 nm) at 40 kV and 40 mA. Scanning electron microscopy (SEM) images were obtained with a Nova Nano SEM450. Transmission electron microscopy (TEM) images were obtained with a JEOL 2100F transmission electron microscope under 200 kV accelerating voltage. Fourier transform infrared spectra (FT-IR) were recorded with a Bruker VECTOR22 spectrometer using KBr. X-ray photoelectron spectrometry (XPS) was performed on a Thermo Scientific KAlpha X-ray photoelectron spectrometer. The N_2 adsorption-desorption isotherms were measured using a Micromeritics ASAP 2020 analyzer. The samples were outgassed at 120 °C for 12 h under vacuum conditions before the test. The total pore volume of the samples was estimated from the amount adsorbed at the highest P/P_0 (ca. 0.99) by the Barrett-Joyner-Halenda (BJH) model. The surface water contact angle (WCA) of the sample was measured by the optical contact angle measuring device (KRUSS DSA-100, Germany). The fully automated chemisorption analyzer (Micromeritics, Autochem II 2920) was applied to determine the number of accessible Pd sites in different catalysts via CO titration at 293 K.

2. Experimental Section.

2.1 Synthesis of COF-OH

COF-OH was prepared according to the method in reference with minor modification.[1] Firstly, TAPB (0.080 mmol), DHTP (0.12 mmol), 1,2-dichlorobenzene (1 mL), n-butylalcohol (1 mL) and acetic acid (6 M, 0.2 mL) were added to the reaction tube with side neck and degassed via three freeze–pump–thaw cycles. Then, the mixture was reacted in an oil bath at 120 °C for 3 days. When the reaction was completed, the resulting precipitate was washed six times with tetrahydrofuran. Then, the obtained precipitate was purified with a Soxhlet extractor containing tetrahydrofuran for 24 h. Finally, the product named COF-OMe was dried under vacuum at 60 °C for 12 h for further use (Scheme S1).

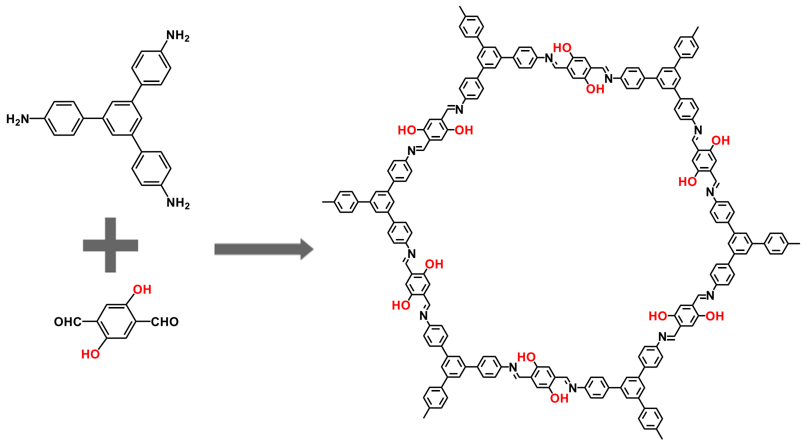

Scheme S1. Synthetic routine for COF-OH.

2.2 Synthesis of COF-ONa

COF-ONa was prepared according to the method in reference with minor modification.[1] COF-ONa was obtained by the treatment of COF-OH (100 mg) with NaOH aqueous solution (1 M, 20 mL). After being stirred at room temperature for 3 h, the product was isolated by filtration, washed by water and acetone, and dried under vacuum at 50 °C for further use (Scheme S2).

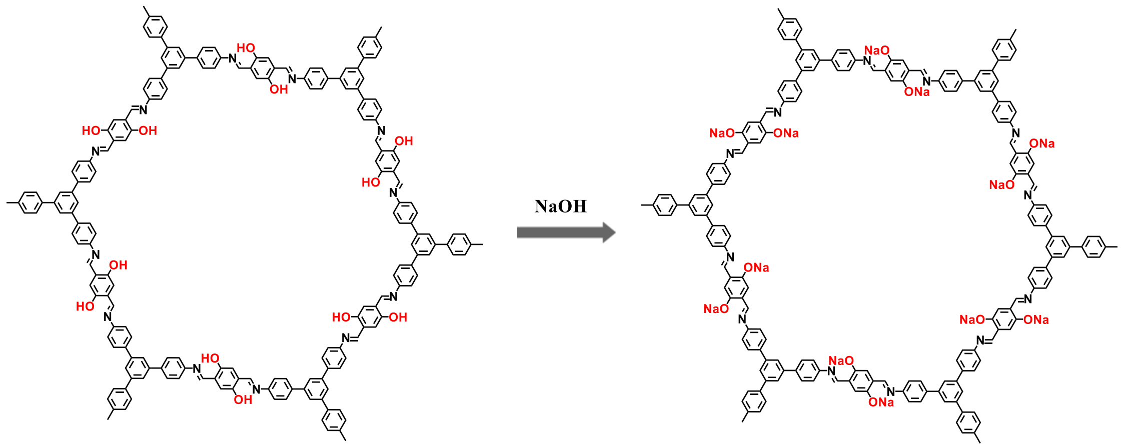

Scheme S2. Synthetic routine for COF-ONa.

2.3 Synthesis of racemic amides

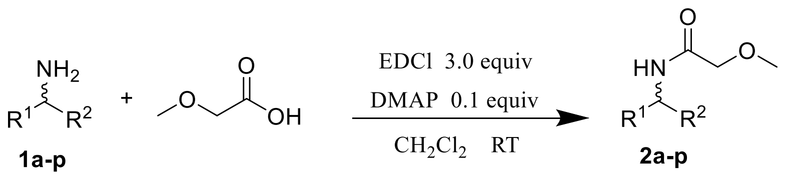

A mixture of the corresponding amine (4.0 mmol), methoxyacetic acid (2.0 mmol) and DMAP (0.4 mmol) was dissolved in CH_2Cl_2 (5 mL) and the solution cooled in an ice bath. Then N-(3-Dimethylaminopropyl)-N-ethylcarbodiimide hydrochloride (EDCI, 3.0 mmol) was added in a single portion and the mixture was stirred for 10 min at 0 ºC and then room temperature until TLC indicated complete consumption of the amine. The reaction mixture was then poured into H_2O and extracted with CH_2Cl_2. The combined organic extracts were dried over Na_2SO_4 and evaporated to give a crude material. The residue was purified by a column chromatography on a silica gel with petroleum ether/ethyl acetate (1:1) as an eluent to offer the pure product.

2.4 Racemization of (*S*)-1-PEA

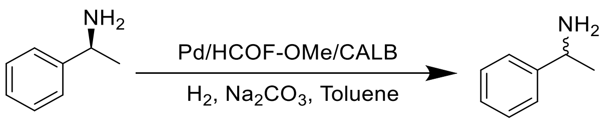

(*S*)-1-PEA (0.50 mmol), Na_2CO_3 (0.3 mmol) and Pd/COF or Pd/COF-X/CALB (1 mg, base on Pd) were suspended in dry toluene (2 mL) in a Schlenk tube. The tube was

evacuated and filled with nitrogen two times, before it was evacuated one final time and filled with hydrogen gas. A hydrogen replacement balloon was connected to the tube. The reaction is stirred at a specified temperature and regularly sampled for detection. The yield and ee value were determined by GC. The samples were filtered with a 0.22 μm membrane before detection.

The turnover frequency (TOF) of the racemization of (*S*)-1-PEA catalyzed by Pd was calculated by using Equation (S1):

$$TOF = \frac{moles\ of\ coverted\ substrate}{moles\ of\ Pd*t} \tag{S1}$$

The molar number of substrate conversion to (*S*)-1-PEA is the amount of substance converted to (*R*)-1-PEA; The number of moles of Pd is 9.4×10^{-6}; The reaction time was 0.5 h (based on the reaction time of 0-0.5 h).

2.5 Kinetic resolution of 1-PEA

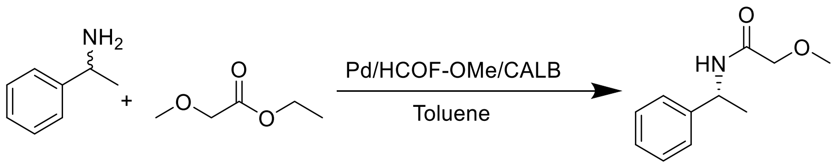

1-PEA (0.25 mmol), ethyl methoxyacetate (0.5 mmol) and CALB or Pd/COF-X/CALB (3 mg, base on CALB) were suspended in dry toluene (2 mL) in a Schlenk tube. The reaction is stirred at a specified temperature and regularly sampled for detection. The yield and ee value were determined by GC. The samples were filtered with a 0.22 μm membrane before detection.

2.6 Dynamic Kinetic Resolution of Primary Amines

1-PEA (0.25 mmol), ethyl methoxyacetate (0.5 mmol), Na_2CO_3 (0.3 mmol) and Pd/COF-X/CALB (40 mg) were suspended in dry toluene (2 mL) in a Schlenk tube. The tube was evacuated and filled with nitrogen two times, before it was evacuated one final time and filled with hydrogen gas. A hydrogen replacement balloon was connected to the tube. The reaction is stirred at a specified temperature and regularly sampled for

detection. The yield and ee value were determined by GC. The samples were filtered with a 0.22 μm membrane before detection.

2.7 The thermal stability of free CALB and Pd/COF-OMe/CALB

The thermal stability was determined by putting free CALB and Pd/COF-OMe/CALB into toluene at 60 °C, 70 °C and 80 °C, and incubating for a period of time. The kinetic resolution activity of Pd/COF-OMe/CALB as a catalyst for primary amines was measured every 12 h, and the remaining activity was calculated according to equation (S2).

$$\text{Remaining activity (\%)}=\frac{\text{Residual enzyme activity of treated samples}}{\text{Initial enzyme activity of the sample}}\times 100\% \qquad \text{(S2)}$$

2.8 1-PEA adsorption studies

1-PEA (0.25 mmol) and Pd/COF-X/CALB (40 mg) were immersed into toluene (2 mL) and the mixture was stirred at 25 °C for a period of time. The concentration of 1-PEA in the supernatant was analyzed by GC with pentadecane as an internal standard. The 1-PEA adsorption capacity was determined by the following equation:

$$q=\frac{(C_i-C_f)V}{m}\times 100\% \qquad \text{(S3)}$$

Where q is the amount of 1-PEA adsorbed (mmol/$g_{Pd/COF-X/CALB}$); C_i is the initial concentration of the 1-PEA (mmol/L); C_f is the final concentration after adsorption (mmol/L); V is the volume of solution (L); and m is the amount of added Pd/COF-X/CALB (g).

2.9 Reusability of Pd/HCOF-OMe/CALB

1-PEA (0.25 mmol), ethyl methoxyacetate (0.5 mmol), Na_2CO_3 (0.3 mmol) and Pd/COF-X/CALB (40 mg) were suspended in dry toluene (2 mL) in a Schlenk tube. The tube was evacuated and filled with nitrogen two times, before it was evacuated one final time and filled with hydrogen gas. A hydrogen replacement balloon was connected to the tube. The reaction is stirred at 70 °C for 10 h. The yield and ee value were determined by GC. The samples were centrifugally separated and filtered with a 0.22

μm membrane before detection. The precipitate Pd/HCOF-OMe/CALB was recovered and washed twice with toluene (3 mL) to enter the next cycle.

2.10 Calculation method

Models of COF/Pd nanoparticles (NP) were built to calculate charge transfer of Pd nanoparticles and electron density difference ($\Delta\rho$) via GFN0-xTB method.[2] $\Delta\rho$ is calculated via equation S4, where $\rho_{\mathrm{COF/Pd}}$, ρ_{COF}, and ρ_{Pd} represent electron density of COF/Pd, COF, and Pd, respectively. In these models, Pd NP is a cluster consisting of 561 Pd atoms. Its size is 3 nm, which is almost similar to TEM result. To incarnate the effect of COF pore canal on accommodating and anchoring the Pd NP, COF unit consists of six single layer COF fragments via AA stacking. Then, Gibbs free energies of related structures were also calculated during the process of (*S*)-1-PEA racemization via Pd NP catalyst. GFN0-xTB method was taken to optimize structures and perform frequency analysis. In this process, pure Pd NP catalyst and charged Pd catalyst (+14, COF-ONa/Pd; +13, COF-OH/Pd; +12, COF-OMe/Pd) were thought to compare catalytic effects of different COF composite catalysts.

$$\Delta\rho = \rho_{\mathrm{COF/Pd}} - \rho_{\mathrm{COF}} - \rho_{\mathrm{Pd}} \qquad \text{(S4)}$$

3. Figures and Tables

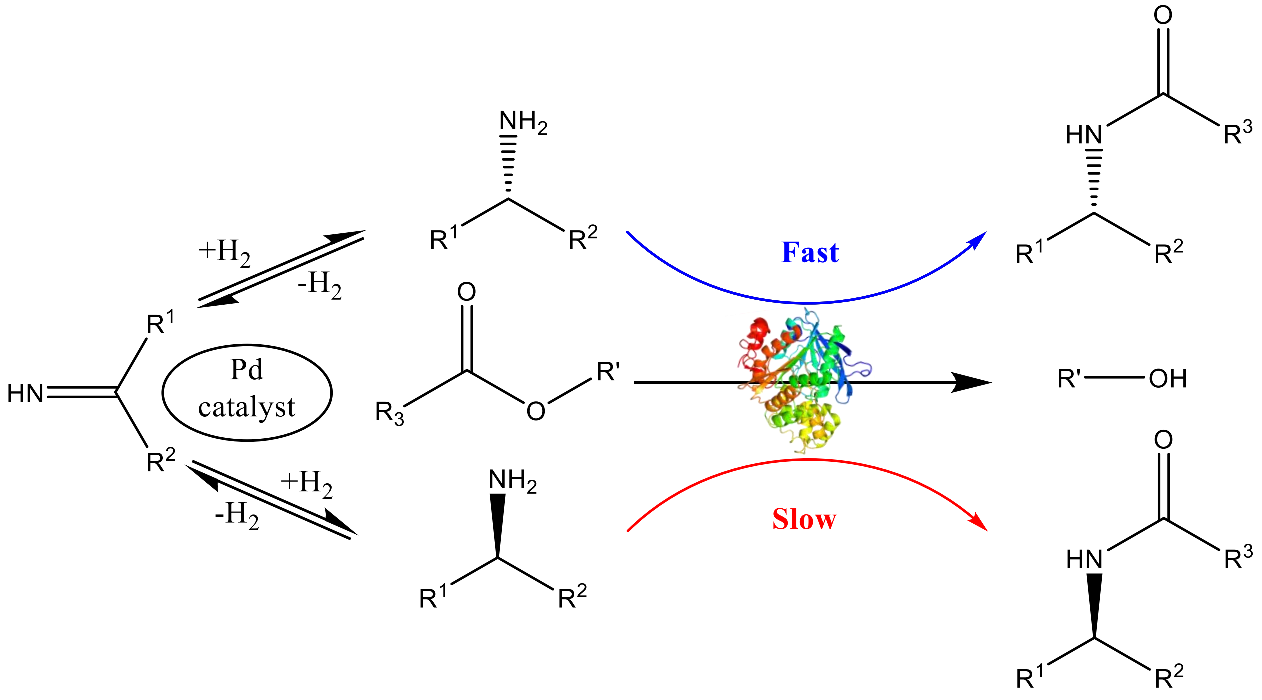

Figure S1. Schematic representation of dynamic kinetic resolution of chiral amines with esters as resolving agents.

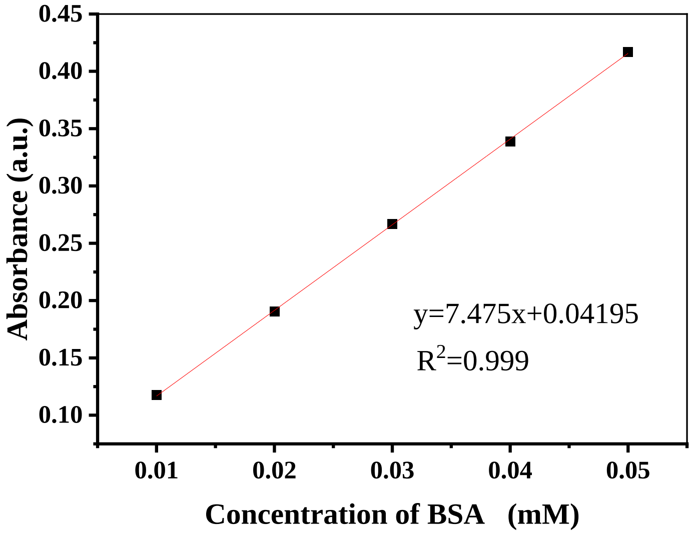

Figure S2. Standard curve of BSA concentration.

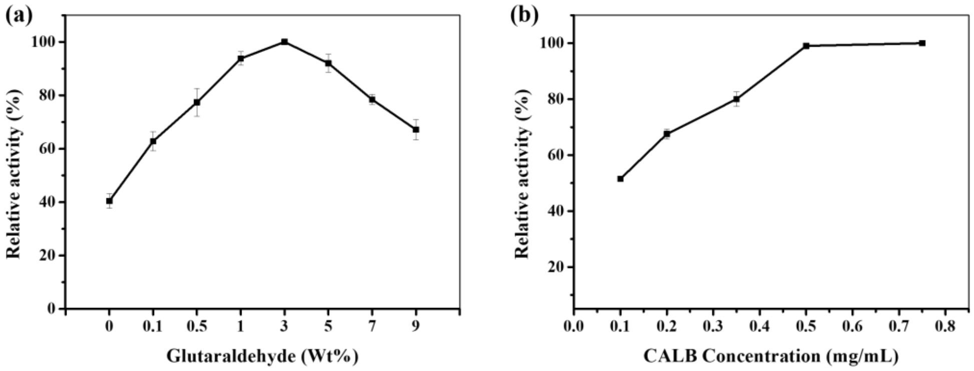

Figure S3. Influence of (a) GA concentration and (b) initial CALB concentration on relative activity of Pd/COF-OMe/CALB..

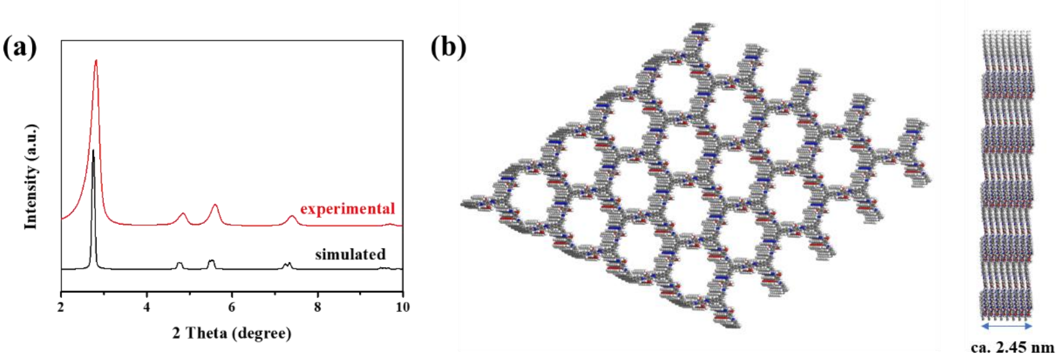

Figure S4. (a) PXRD patterns of COF-OMe: experimental patterns (red) and the simulated pattern for eclipsed AA stacking mode (black); (b) Graphic view of COF-OMe AA stacking mode (The layer spacing is 0.35 nm).

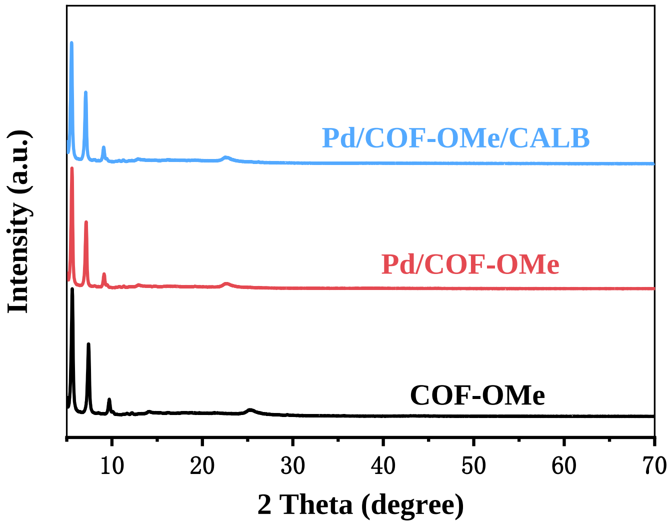

Figure S5. Wide-angle PXRD patterns of COF-OMe, Pd/COF-OMe and Pd/COF-OMe/CALB.

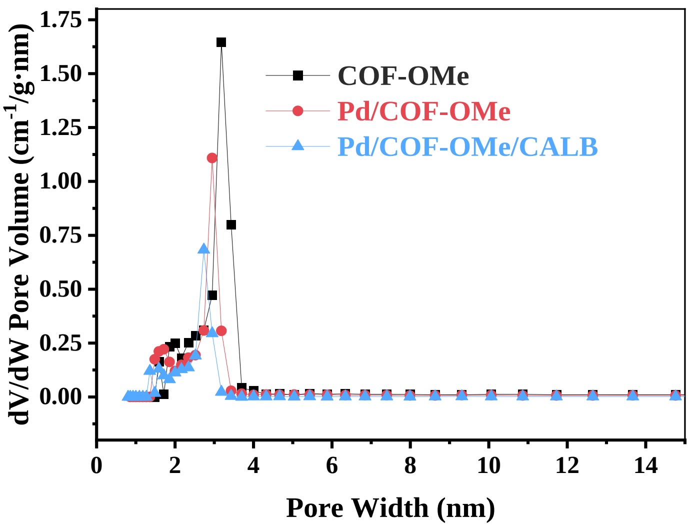

Figure S6. Pore size distributions of COF-OMe, Pd/COF-OMe and Pd/COF-OMe/CALB.

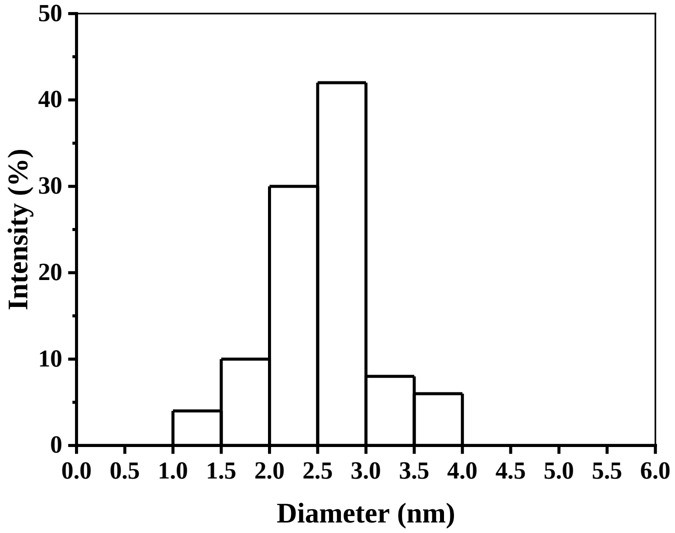

Figure S7. Particle size profile of Pd NPs in Pd/COF-OMe/CALB.

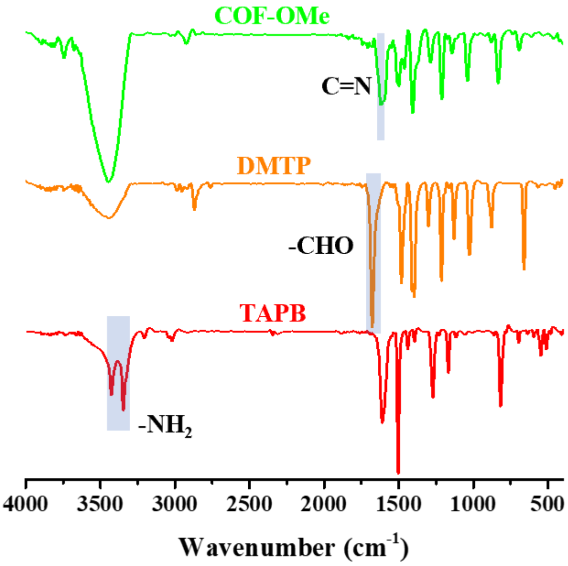

Figure S8. FT-IR spectra of TAPB, DMTP and COF-OMe

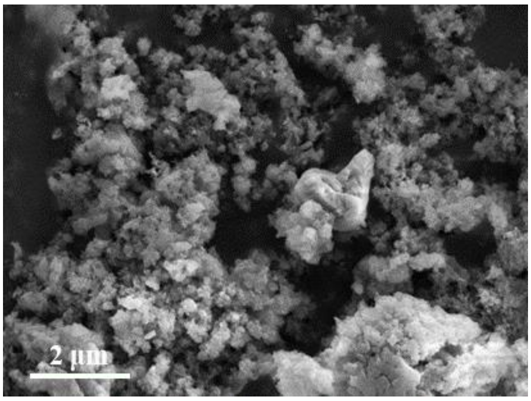

Figure S9. SEM image of Pd/COF-OMe/CALB.

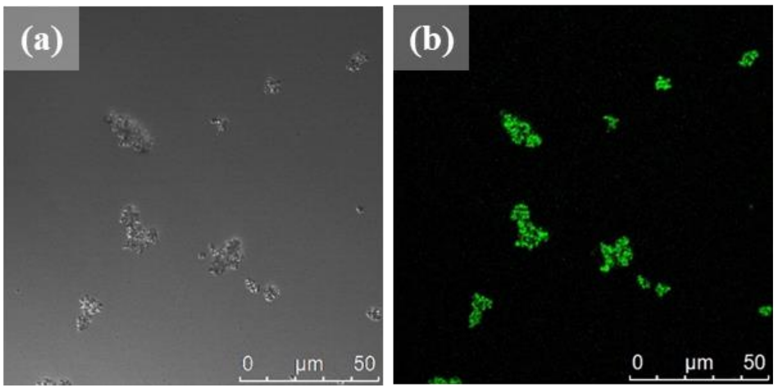

Figure S10. CLSM images of Pd/COF-OMe/FITC-CALB in (a) optical and (b) fluorescence.

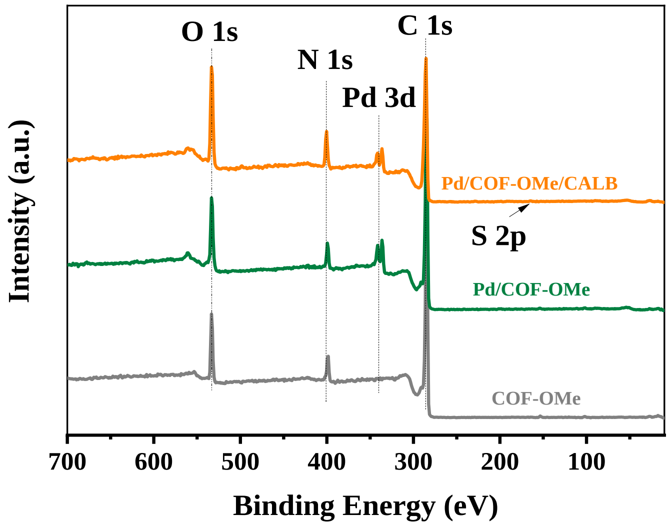

Figure S11. XPS survey spectra of COF-OMe, Pd/COF-OMe and Pd/COF-OMe/CALB.

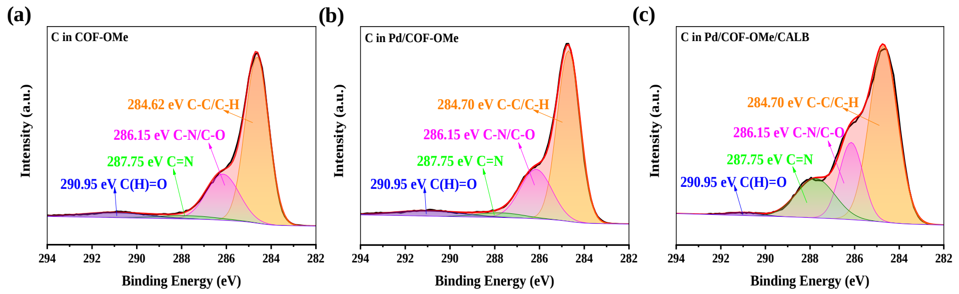

Figure S12. C 1s regions in the XPS spectra of (a) COF-OMe, (b) Pd/COF-OMe and (c) Pd/COF-OMe/CALB.

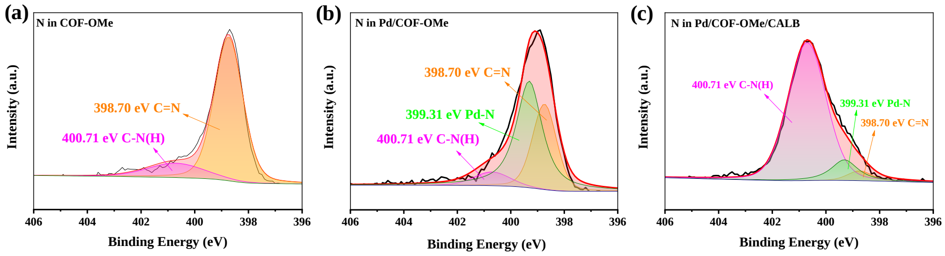

Figure S13. N 1s regions in the XPS spectra of (a) COF-OMe, (b) Pd/COF-OMe and (c) Pd/COF-OMe/CALB.

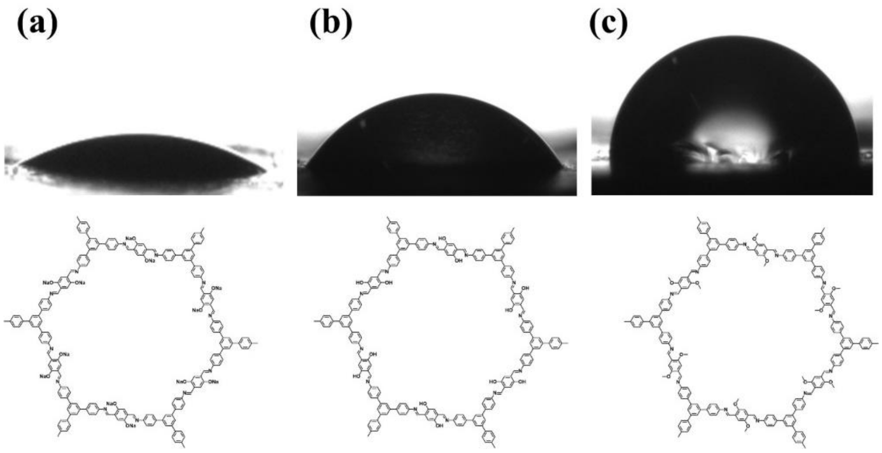

Figure S14. The water contact angles of (a) Pd/COF-ONa/CALB, (b) Pd/COF-OH/CALB and (c) Pd/COF-OMe/CALB.

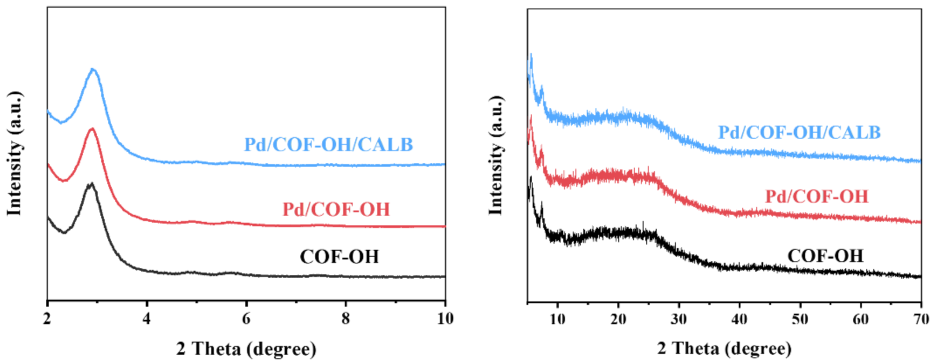

Figure S15. (a) Small-angle XRD and (b) wide-angle XRD patterns of COF-OH, Pd/COF-OH and Pd/COF-OH/CALB.

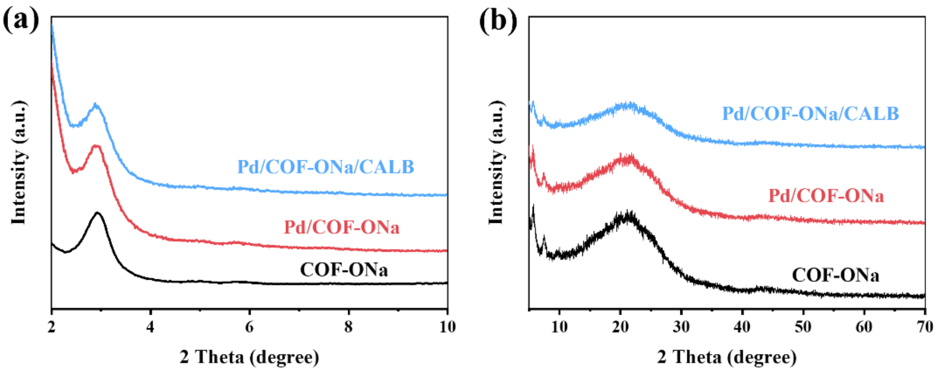

Figure S16. (a) Small-angle XRD and (b) wide-angle XRD patterns of COF-ONa, Pd/COF-ONa and Pd/COF-ONa/CALB.

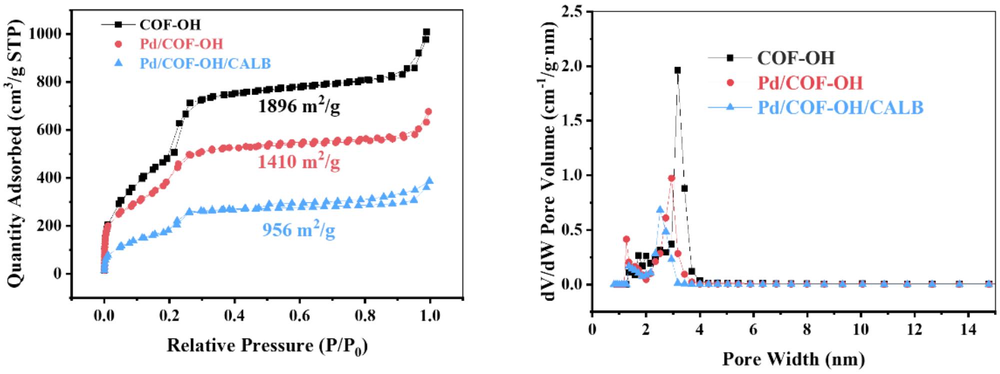

Figure S17. (a) Nitrogen sorption isotherm curve and (b) pore-size distribution of COF-OH, Pd/COF-OH and Pd/COF-OH/CALB.

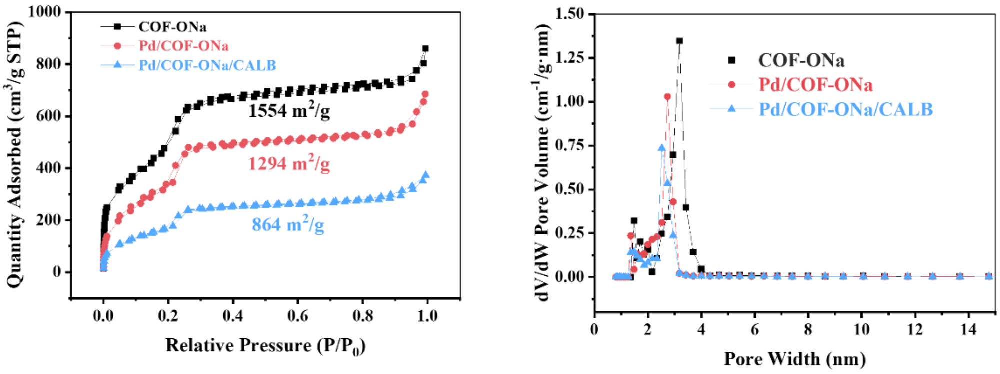

Figure S18. (a) Nitrogen sorption isotherm curve and (b) pore-size distribution of COF-ONa, Pd/COF-ONa and Pd/COF-ONa/CALB.

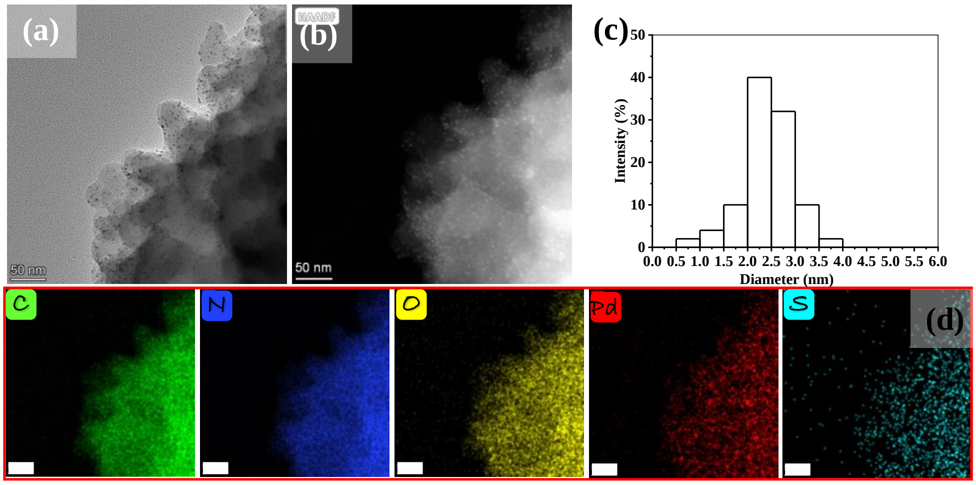

Figure S19. (a) TEM image, (b) HAADF image, (c) size distribution of Pd NPs and (d) EDX elemental mapping of Pd/COF-OH/CALB (scale: 50 nm).

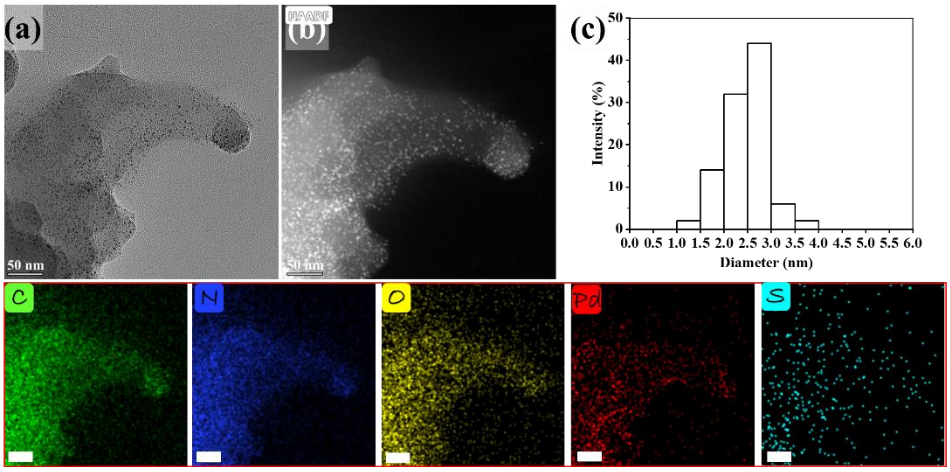

Figure S20. (a) TEM image, (b) HAADF image, (c) size distribution of Pd NPs and (d) EDX elemental mapping of Pd/COF-ONa/CALB (scale: 50 nm).

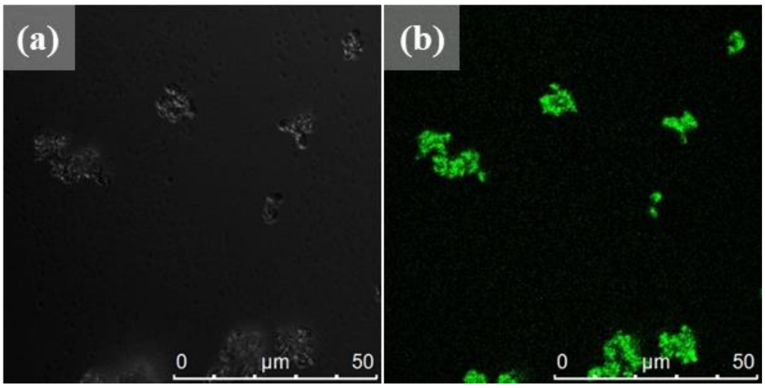

Figure S21. CLSM images of Pd/COF-OH/FITC-CALB in (a) optical and (b) fluorescence.

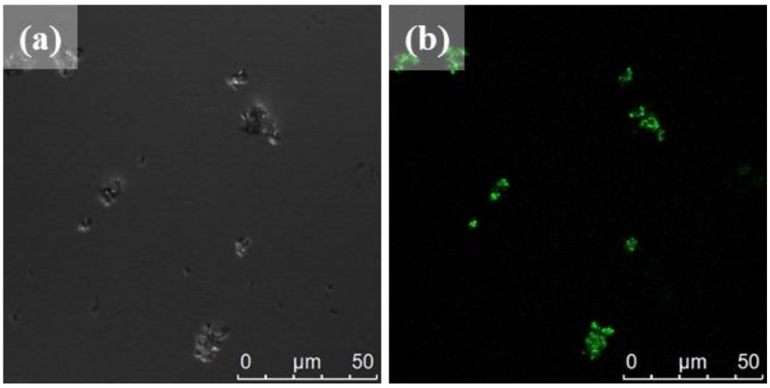

Figure S22. CLSM images of Pd/COF-Na/FITC-CALB in (a) optical and (b) fluorescence.

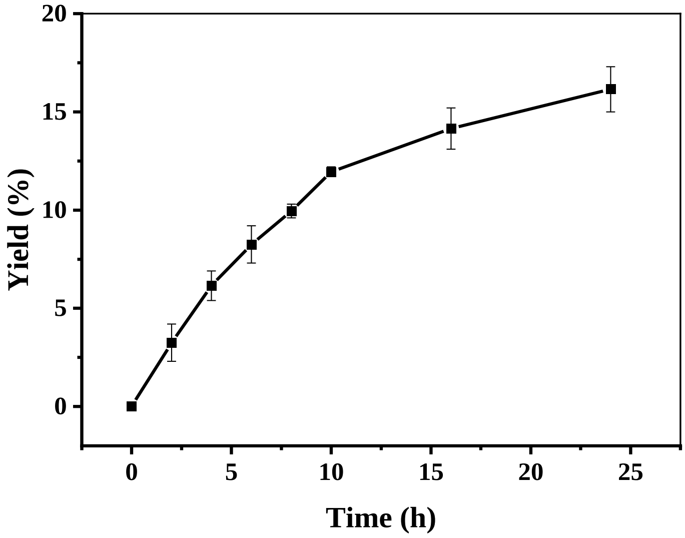

Figure S23. The kinetic resolution of 1-phenethylamine catalyzed by free CALB. Reaction conditions: 1-PEA (0.25 mmol), ethyl methoxyacetate (0.5 mmol), toluene (2 mL) and CALB (3 mg).

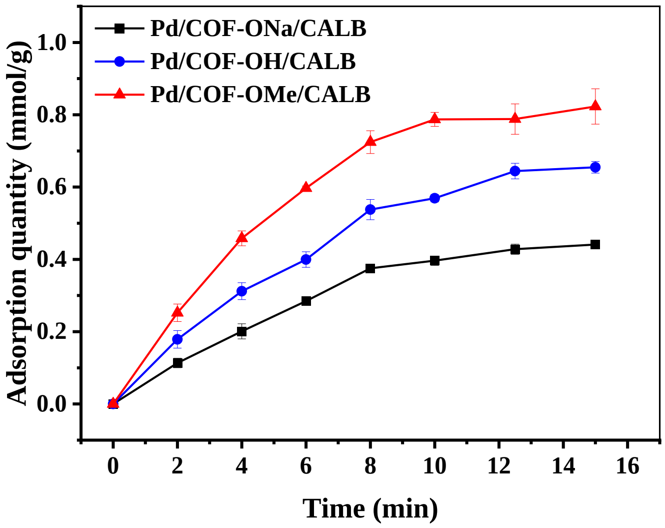

Figure S24. Kinetics of 1-phenethylamine adsorption by Pd/COF-ONa/CALB, Pd/COF-OH/CALB and Pd/COF-OMe/CALB.

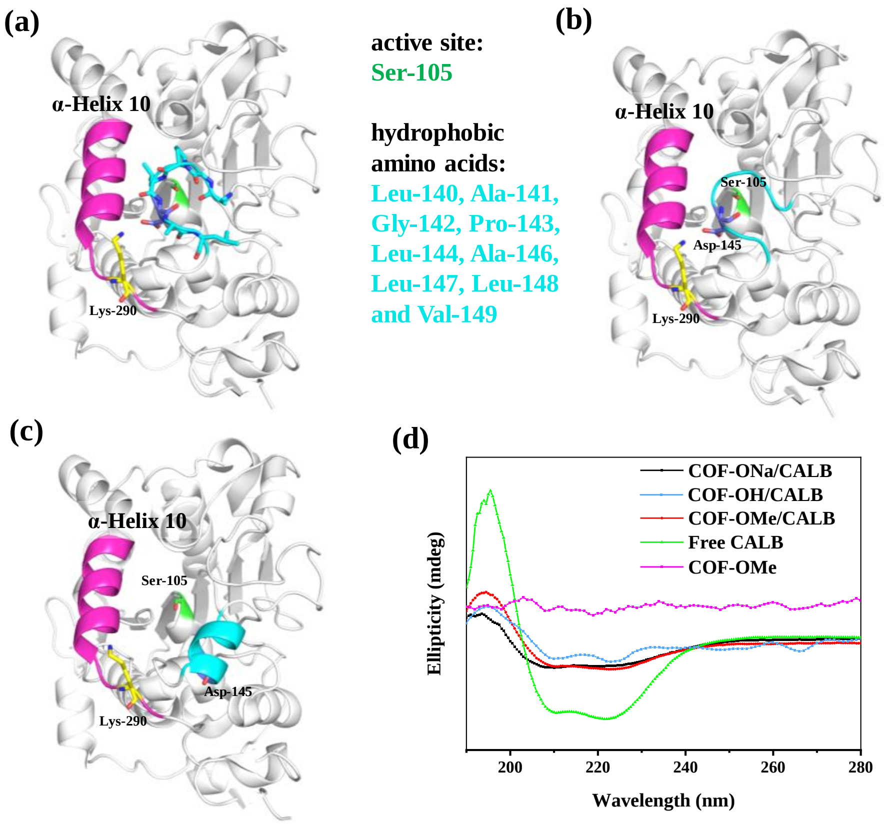

Figure S25. (a) Hydrophobic amino acids in α-helix 5; Representation of CALB at (b) closed and (c) open conformations; (d) CD spectra of COF-X/CALB and free CALB.

The presence of a lid with many hydrophobic amino acids and a salt bridge formed by the lid above the active site of CALB make the active site inaccessible.[3] The hydrophobic force allows the lid to open and undergo a conformational change from a loop structure to an α-helix.[1] It is known that CD spectroscopic analysis in the far-UV region provides information about the secondary structure of the protein. The free CALB showed typical features of *α*-helix structure with two distinct negative bands at 210 and 221 nm. The ellipticity at·221nm in·COF-OMe/CALB is lower than that of COF-OH/CALB and·COF-ONa/CALB, so the α-helix content follows the order from high to low as COF-OMe/CALB > COF-OH/CALB > COF-ONa/CALB.[1, 4] This is due to the decrease of α-helix in CALB covalent action and the increase of α-helix in hydrophobic action. The content of α-helix in COF-X/CALB is consistent with the activity of Pd/COF-X/CALB.

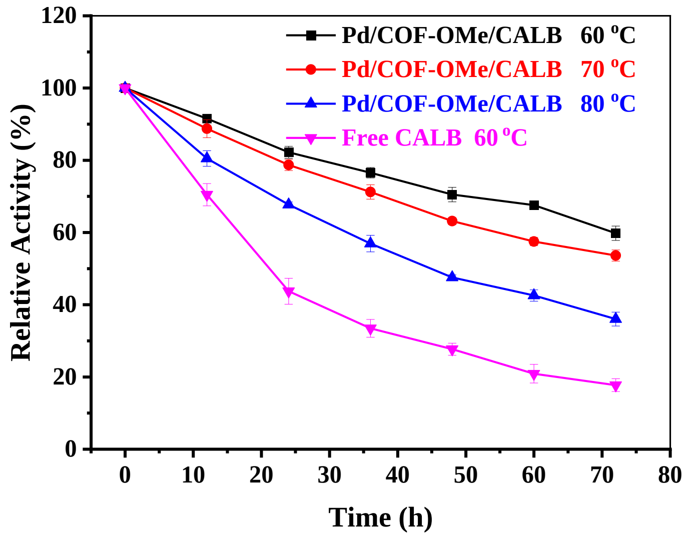

Figure S26. Thermal stability of free CALB and Pd/COF-OMe/CALB in toluene.

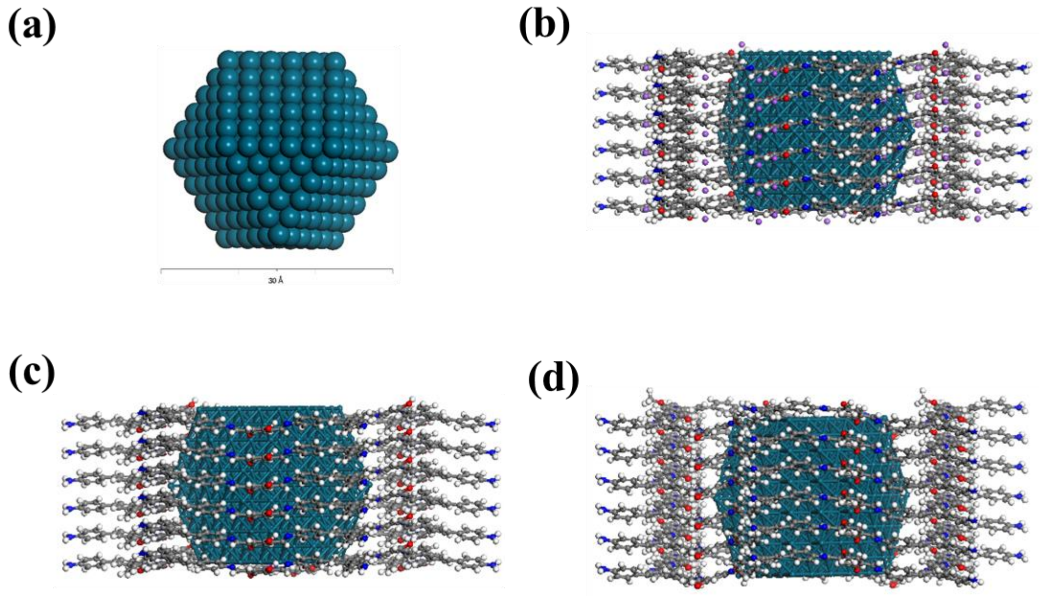

Figure S27. (a) Model of Pd NPs; Lateral molecular models of (b) Pd/COF-ONa, (c) Pd/COF-OH and (d) Pd/COF-OMe.

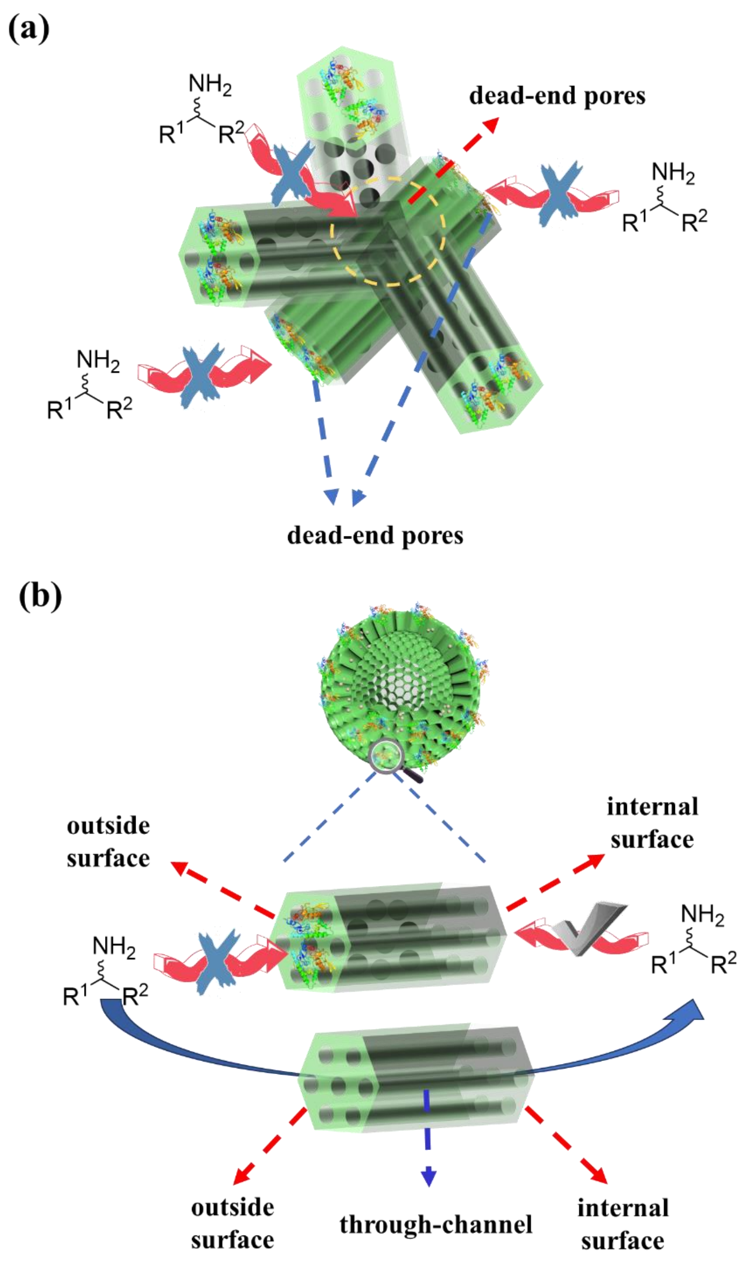

Figure S28. Schematic diagram of mass transfer of substrate molecules in (a) Pd/COF-OMe/CALB and (b) Pd/HCOF-Me/CALB catalytic system.

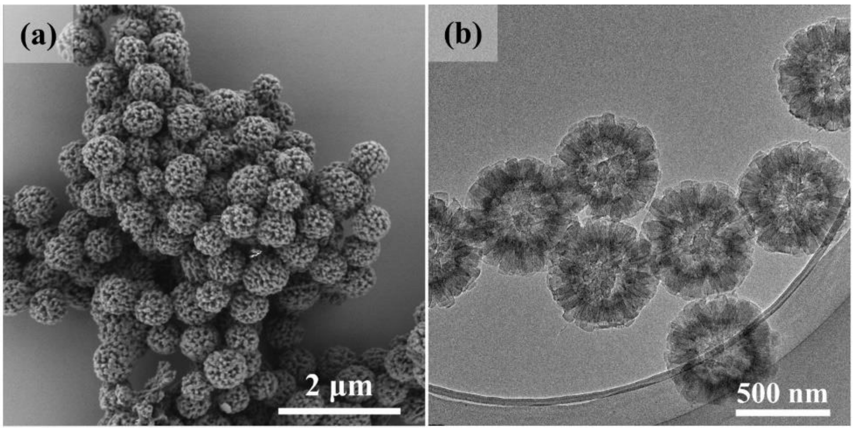

Figure S29. (a) SEM and (b) TEM images of HCOF-OMe.

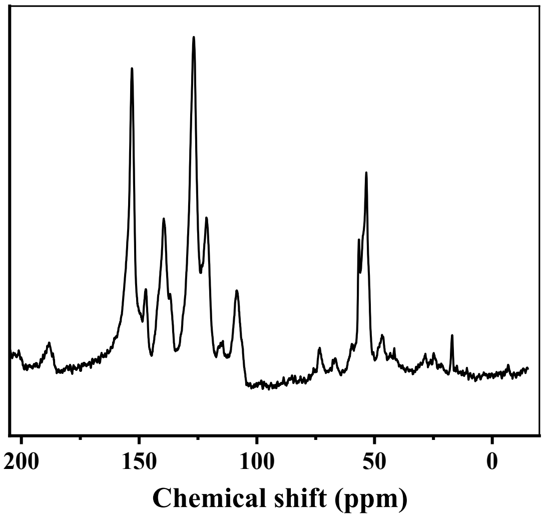

Figure S30. The ^{13}C CP-MAS NMR spectrum of HCOF-OMe

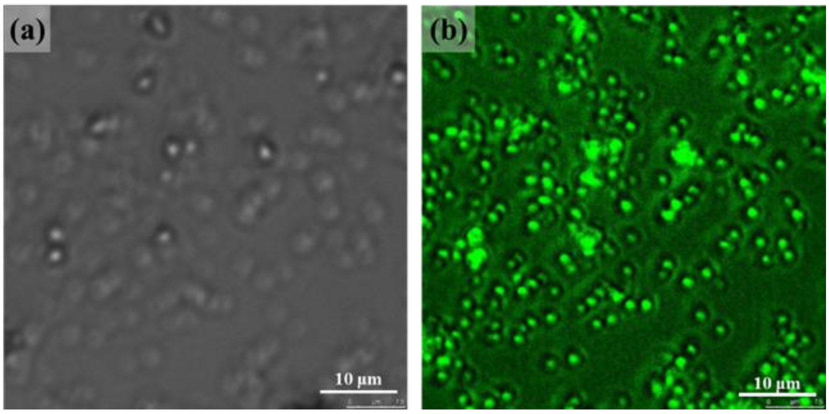

Figure S31. CLSM images of Pd/HCOF-OMe/FITC-CALB in (a) optical and (b) fluorescence.

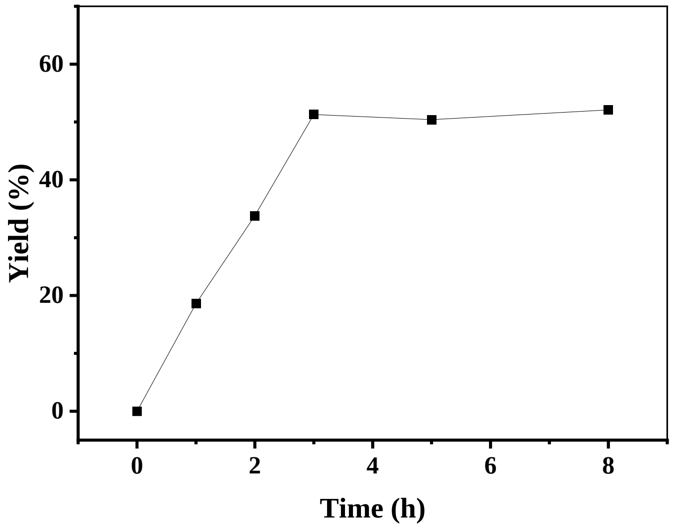

Figure S32. Filtration test of Pd/HCOF-OMe/CALB for the reaction.

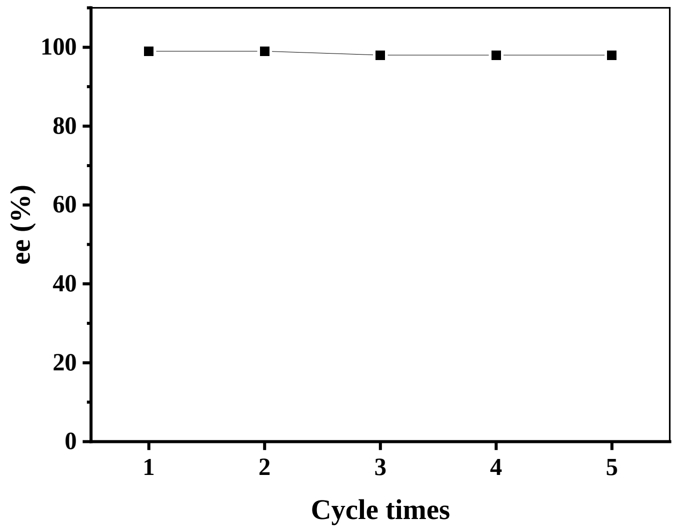

Figure S33. Recyclability of Pd/HCOF-OMe/CALB for DKR of 1-PEA.

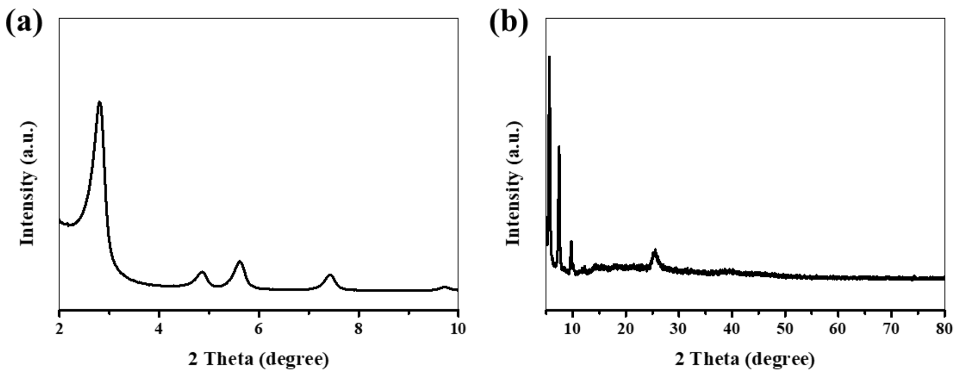

Figure S34. (a) Small-angle and (b) wide-angle PXRD patterns of Pd/HCOF-OMe/CALB after 5 times of cascade reaction.

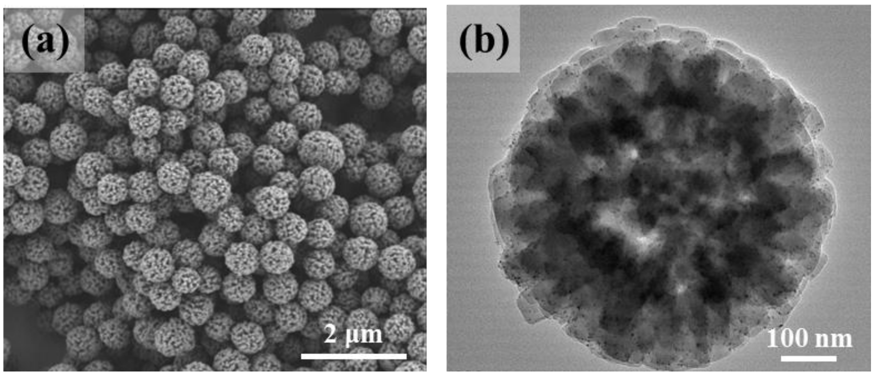

Figure S35. (a) SEM and (b) TEM images of Pd/HCOF-OMe/CALB after 5 times of cascade reaction.

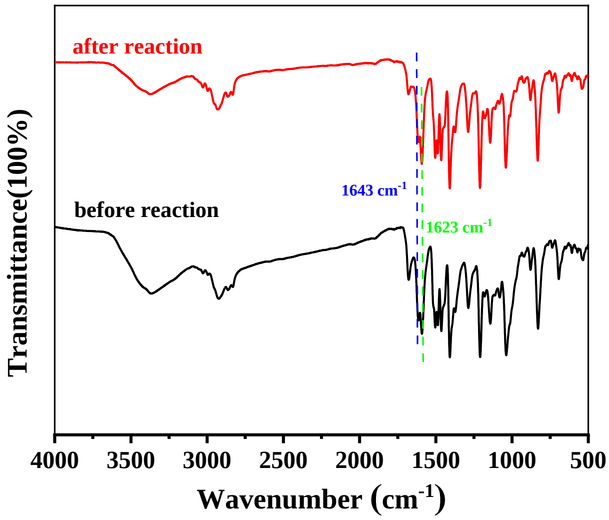

Figure S36. FT-IR spectra of Pd/HCOF-OMe/CALB before and after cascade reaction.

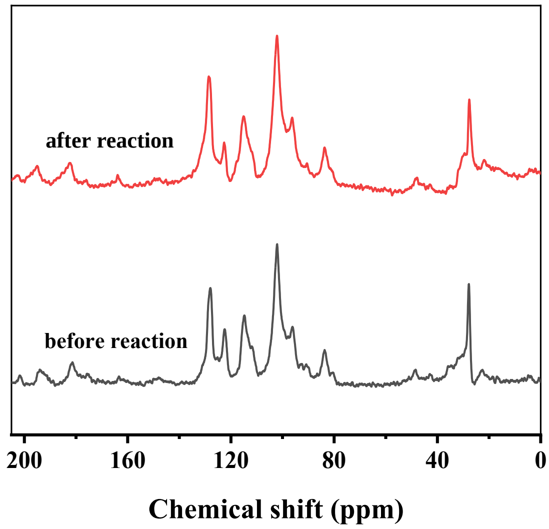

Figure S37. The ^{13}C CP-MAS NMR spectra of Pd/HCOF-OMe/CALB before and after cascade reaction.

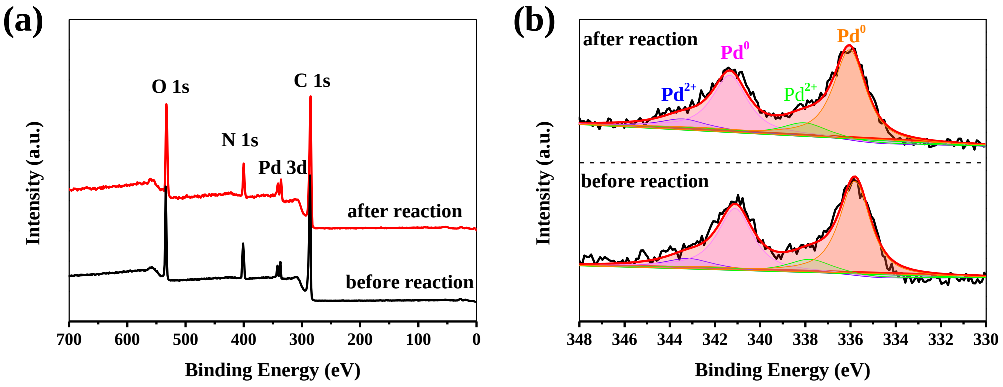

Figure S38. XPS of Pd/HCOF-OMe/CALB before and after cascade reaction: (a) wide scan spectrum and (b) high-resolution spectra of Pd 3d.

Table S1. Effect of different catalysts on DKR[a].

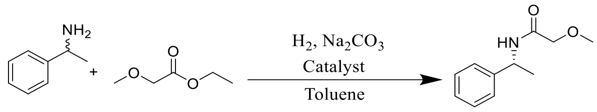

Entry	Catalyst	Time (h)	Yield[b] (%)	ee[b] (%)
1	Pd/COF-ONa/CALB	10	58	> 99
2	Pd/COF-ONa/CALB	24	91	> 99
3	Pd/COF-OH/CALB	10	76	> 99
4	Pd/COF-OH/CALB	16	91	> 99

[a)]Reaction conditions: All reactions were carried out in dry toluene (2.0 mL) with 1-PEA (0.25 mmol), ethyl methoxyacetate (0.5 mmol), catalyst (40 mg) and dry Na_2CO_3 (20 mg). [b)] Determined by gas chromatography and pentadecane as internal standard.

Table S2. CO chemisorption measurements at 293K over Pd/HCOF-OMe, Pd/HCOF-OMe/CALB, Pd/COF-OMe and Pd/COF-OMe/CALB

Entry	Catalysts	Amount of Pd (wt%)	Dispersion sites ($mol_{active\ sites}/mol_{metal,}$ %)	Average particle diameter (nm)
1	Pd/HCOF-OMe	3.13	45.4	2.47
2	Pd/HCOF-OMe/CALB	2.77	43.6	2.56
3	Pd/COF-OMe	2.91	43.8	2.55
4	Pd/COF-OMe/CALB	2.70	33.6	3.14

4. ^{1}H NMR spectra of the products recorded in $CDCl_3$.

(*R*)-2-methoxy-N-(1-phenylethyl)acetamide

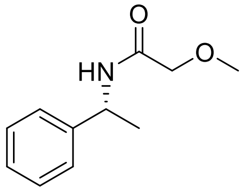

^{1}H NMR (400 MHz, Chloroform-*d*) δ 7.39 (d, *J* = 8.2 Hz, 16H), 7.36 – 7.29 (m, 10H), 6.79 (s, 4H), 5.22 (p, *J* = 7.2 Hz, 5H), 4.00 – 3.86 (m, 10H), 3.44 (s, 15H), 1.90 (s, 1H), 1.55 (d, *J* = 6.9 Hz, 15H), 1.29 (s, 1H).

(*R*)-2-methoxy-N-(1-(p-tolyl)ethyl)acetamide

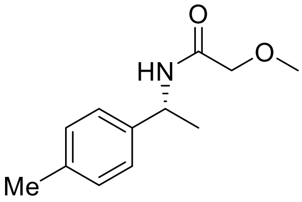

^{1}H NMR (400 MHz, Chloroform-*d*) δ 7.25 (s, 2H), 7.19 (d, *J* = 7.6 Hz, 2H), 6.79 – 6.73 (m, 1H), 5.19 (p, *J* = 7.1 Hz, 1H), 3.98 – 3.87 (m, 2H), 3.44 (s, 3H), 2.38 (s, 3H), 1.54 (d, *J* = 6.9 Hz, 3H).

(*R*)-2-methoxy-N-(1-(4-methoxyphenyl)ethyl)acetamide

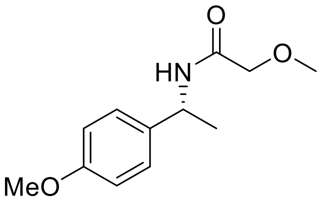

^{1}H NMR (400 MHz, Chloroform-*d*) δ 7.30 (s, 2H), 6.95 – 6.88 (m, 2H), 6.82 – 6.63 (m, 1H), 5.17 (p, *J* = 7.0 Hz, 1H), 3.97 – 3.87 (m, 2H), 3.83 (d, *J* = 2.9 Hz, 3H), 3.42 (d, *J* = 2.9 Hz, 3H), 1.54 (s, 3H).

(*R*)-2-methoxy-N-(1-(2-methoxyphenyl)ethyl)acetamide

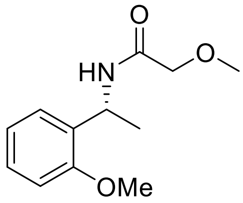

^{1}H NMR (400 MHz, Chloroform-*d*) δ 7.49 – 7.32 (m, 1H), 7.22 (t, *J* = 8.0 Hz, 2H), 6.91 (dd, *J* = 11.5, 7.8 Hz, 2H), 5.37 – 5.25 (m, 1H), 3.88 (d, *J* = 1.3 Hz, 3H), 3.66 (d, *J* = 1.3 Hz, 1H), 3.47 (d, *J* = 1.3 Hz, 1H), 3.40 (d, *J* = 1.3 Hz, 3H), 1.47 (dd, *J* = 6.8, 1.3 Hz, 3H).

(*R*)-2-methoxy-N-(4-phenylbutan-2-yl)acetamide

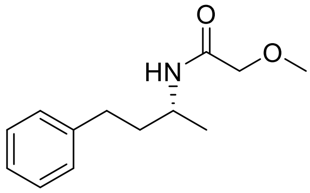

^{1}H NMR (400 MHz, Chloroform-*d*) δ 7.35 – 7.29 (m, 2H), 7.21 (dd, *J* = 7.7, 4.6 Hz, 3H), 6.36 (s, 1H), 4.20 – 4.08 (m, 1H), 3.91 (s, 2H), 3.44 (d, *J* = 2.5 Hz, 3H), 2.69 (dd, *J* = 9.3, 6.8 Hz, 2H), 1.88 – 1.78 (m, 2H), 1.24 (d, *J* = 6.6 Hz, 3H).

(*R*)-2-methoxy-N-(1,2,3,4-tetrahydronaphthalen-1-yl)acetamide

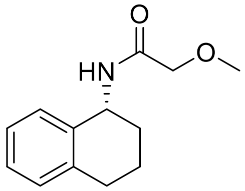

^{1}H NMR (400 MHz, Chloroform-*d*) δ 7.30 – 7.10 (m, 4H), 6.80 (d, *J* = 8.9 Hz, 1H), 5.27 (p, *J* = 5.5 Hz, 1H), 3.99 (s, 2H), 3.43 (s, 3H), 2.92 – 2.75 (m, 2H), 1.95 – 1.82 (m, 4H).

(*R*)-2-ethoxy-N-(1-(naphthalen-1-yl)ethyl)acetamide

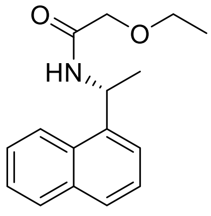

^{1}H NMR (400 MHz, Chloroform-*d*) δ 8.17 (d, *J* = 8.4 Hz, 1H), 7.95 – 7.88 (m, 1H), 7.85 (d, *J* = 8.1 Hz, 1H), 7.61 – 7.49 (m, 4H), 6.82 (s, 1H), 6.03 (dt, *J* = 13.8, 6.8 Hz, 1H), 4.00 (d, *J* = 15.0 Hz, 1H), 3.92 (d, *J* = 14.9 Hz, 1H), 3.37 (d, *J* = 2.4 Hz, 3H), 1.74 (d, *J* = 6.7 Hz, 3H).

(*R*)-2-methoxy-N-(1-(naphthalen-2-yl)ethyl)acetamide

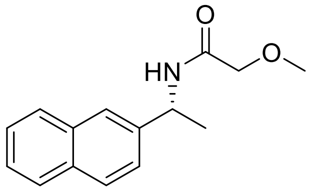

^{1}H NMR (400 MHz, Chloroform-*d*) δ 9.09 – 8.99 (m, 3H), 8.69 (ddt, *J* = 8.5, 7.0, 1.8 Hz, 2H), 8.07 (d, *J* = 7.5 Hz, 1H), 6.61 – 6.51 (m, 1H), 5.17 – 5.12 (m, 2H), 4.89 (d, *J* = 1.3 Hz, 1H), 4.71 (d, *J* = 1.3 Hz, 1H), 4.64 (d, *J* = 1.3 Hz, 2H), 4.54 (d, *J* = 1.3 Hz, 1H), 2.85 (dd, *J* = 6.9, 1.3 Hz, 3H).

(*R*)-N-(heptan-2-yl)-2-methoxyacetamide

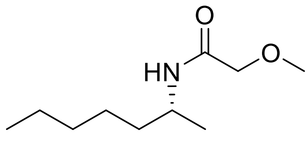

^{1}H NMR (400 MHz, Chloroform-*d*) δ 6.32 (s, 1H), 4.04 (dt, *J* = 13.6, 6.5 Hz, 2H), 3.91 (s, 4H), 3.45 (s, 6H), 1.47 (t, *J* = 7.2 Hz, 4H), 1.33 (h, *J* = 7.8, 6.0 Hz, 13H), 1.18 (d, *J* = 6.5 Hz, 6H), 0.91 (d, *J* = 7.0 Hz, 6H).

(*R*)-N-(1-(4-fluorophenyl)ethyl)-2-methoxyacetamide

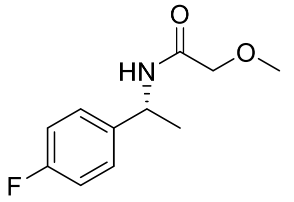

^{1}H NMR (400 MHz, Chloroform-*d*) δ 7.38 (d, *J* = 6.7 Hz, 4H), 6.80 (s, 1H), 5.23 (p, *J* = 7.2 Hz, 1H), 4.00 – 3.88 (m, 2H), 3.45 (s, 3H), 1.56 (d, *J* = 6.9 Hz, 3H).

(*R*)-N-(1-(2-chlorophenyl)ethyl)-2-methoxyacetamide

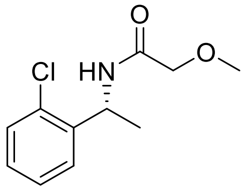

^{1}H NMR (400 MHz, Chloroform-*d*) δ 7.42 (ddd, *J* = 13.0, 7.7, 1.6 Hz, 2H), 7.36 – 7.27 (m, 2H), 7.02 (d, *J* = 8.8 Hz, 1H), 5.53 (p, *J* = 7.2 Hz, 1H), 4.03 – 3.92 (m, 2H), 3.51 (s, 3H), 1.60 (d, *J* = 6.9 Hz, 3H).

(*R*)-N-(1-(3-chlorophenyl)ethyl)-2-methoxyacetamide

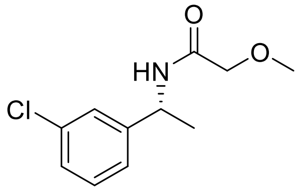

^{1}H NMR (400 MHz, Chloroform-*d*) δ 6.74 – 6.57 (m, 4H), 6.15 (d, *J* = 7.6 Hz, 1H), 4.54 (p, *J* = 7.0 Hz, 1H), 3.30 (t, *J* = 2.7 Hz, 2H), 2.82 (s, 3H), 0.90 (d, *J* = 6.9 Hz, 3H).

(*R*)-N-(1-(4-chlorophenyl)ethyl)-2-methoxyacetamide

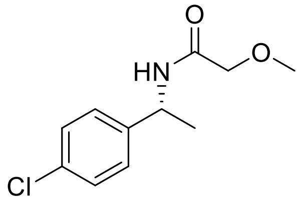

^{1}H NMR (400 MHz, Chloroform-*d*) δ 6.69 – 6.61 (m, 4H), 6.10 (s, 1H), 4.55 – 4.46 (m, 1H), 3.31 – 3.20 (m, 2H), 2.78 (d, *J* = 2.5 Hz, 3H), 0.86 (dd, *J* = 7.0, 2.4 Hz, 3H).

(*R*)-N-(1-(2,4-dichlorophenyl)ethyl)-2-methoxyacetamide

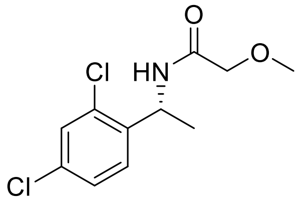

^{1}H NMR (400 MHz, Chloroform-*d*) δ 7.37 (d, *J* = 2.0 Hz, 1H), 7.23 – 7.20 (m, 1H), 6.89 (d, *J* = 7.6 Hz, 1H), 5.37 (t, *J* = 7.2 Hz, 1H), 3.88 (d, *J* = 6.1 Hz, 2H), 3.43 (d, *J* = 1.4 Hz, 3H), 1.50 – 1.47 (m, 3H), 1.19 (dd, *J* = 6.0, 1.4 Hz, 1H).

(*R*)-N-(1-(4-bromophenyl)ethyl)-2-methoxyacetamide

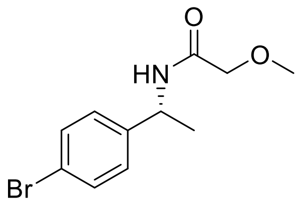

^{1}H NMR (400 MHz, Chloroform-*d*) δ 7.45 (dd, *J* = 8.4, 1.7 Hz, 2H), 7.23 – 7.16 (m, 2H), 6.72 (d, *J* = 8.3 Hz, 1H), 5.11 (p, *J* = 7.3 Hz, 1H), 3.93 – 3.83 (m, 2H), 3.40 (d, *J* = 1.6 Hz, 3H), 1.50 – 1.46 (m, 3H).

(*R*)-2-methoxy-N-(1-(2-methyl-3-(trifluoromethyl)phenyl)ethyl)acetamide

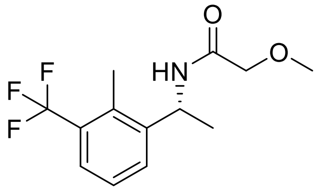

^{1}H NMR (400 MHz, Chloroform-*d*) δ 7.55 (s, 3H), 6.79 (d, *J* = 8.2 Hz, 1H), 5.22 (p, *J* = 7.3 Hz, 1H), 3.91 (dd, *J* = 3.7, 1.3 Hz, 2H), 3.42 (d, *J* = 1.3 Hz, 3H), 1.53 (dd, *J* = 7.0, 1.3 Hz, 3H), 1.20 (dd, *J* = 6.1, 1.3 Hz, 3H).

5. GC and HPLC traces for productions

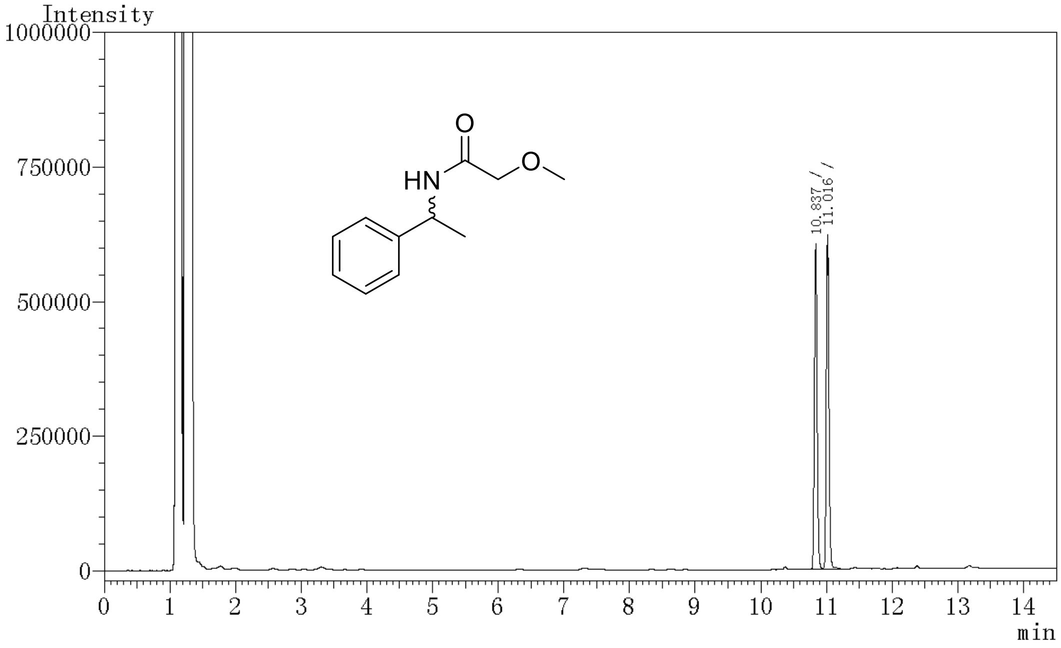

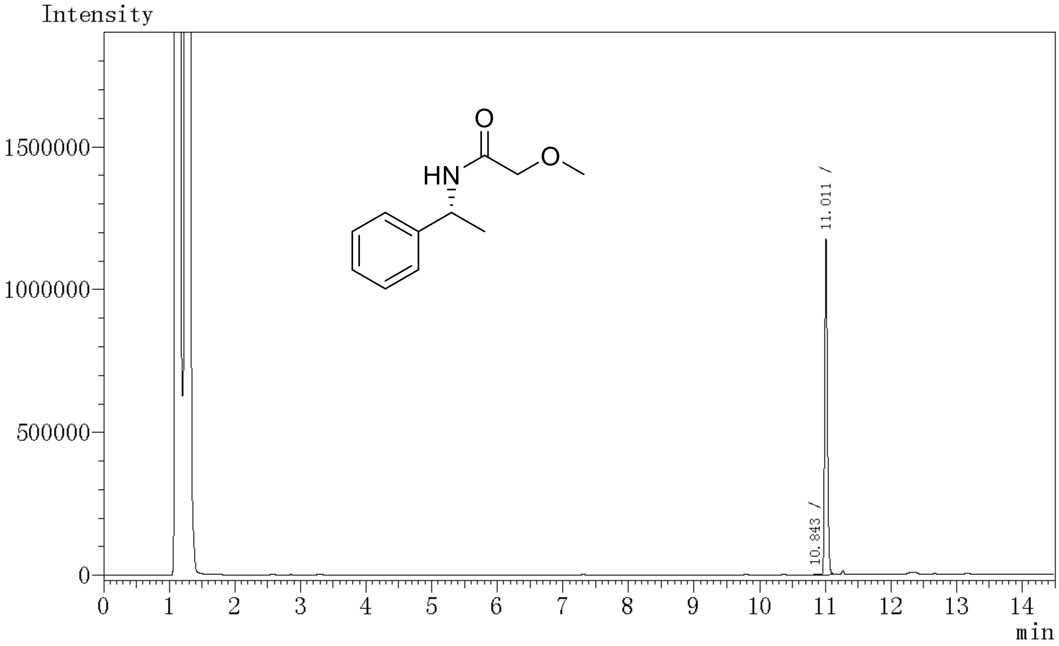

#	Time/min	Area	Height	Area%
1	10.843	4327	940	0.130
2	11.011	3314625	1172780	99.870

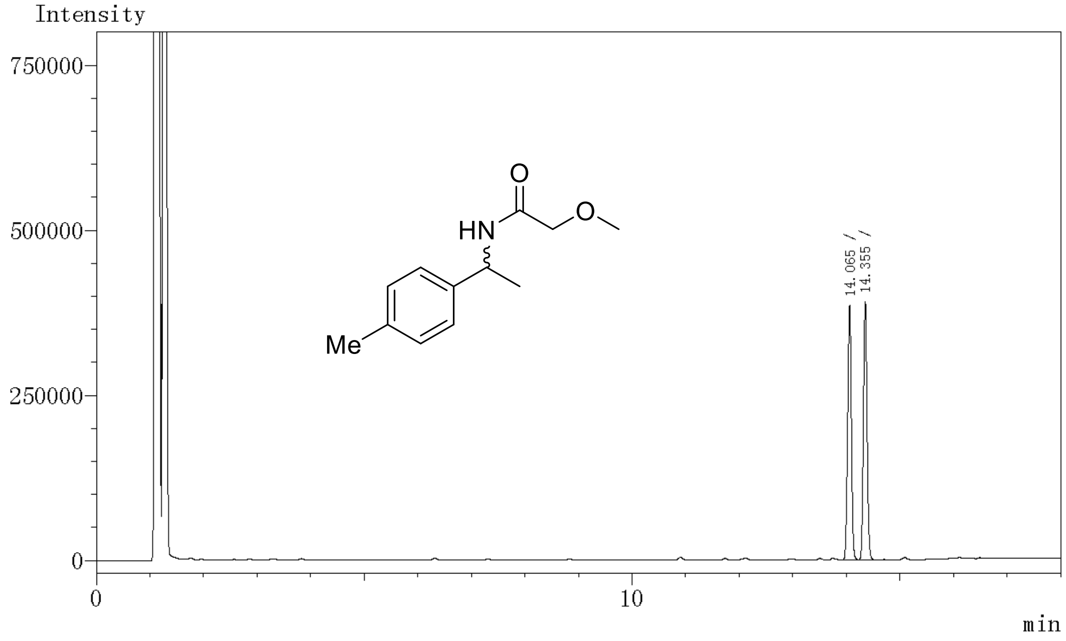

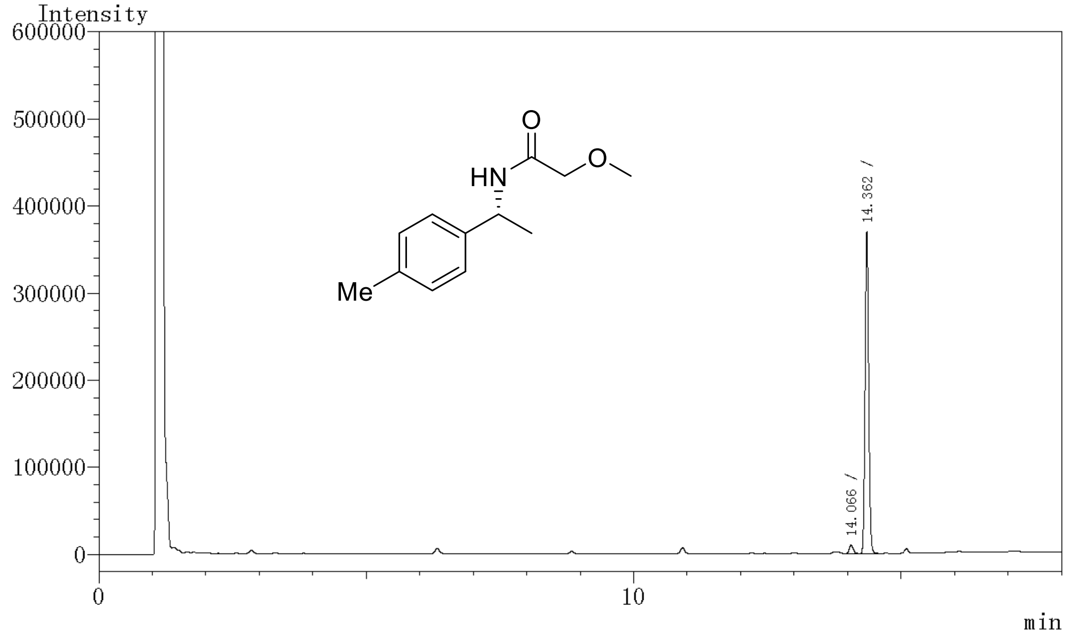

#	Time/min	Area	Height	Area%
1	14.066	48277	9413	2.739
2	14.362	1714603	368618	97.261

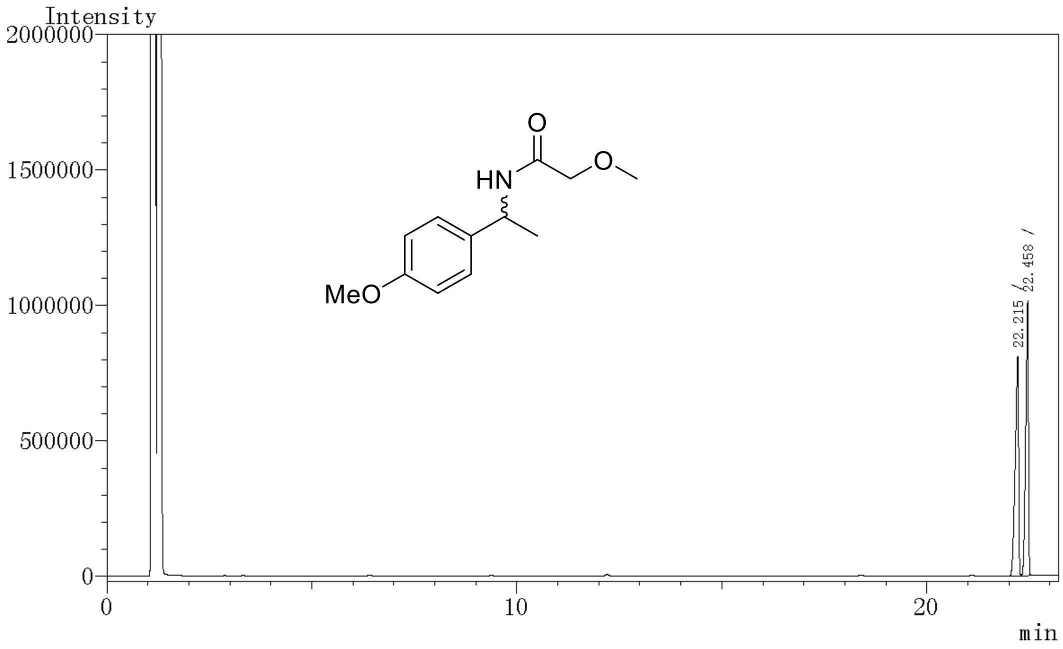

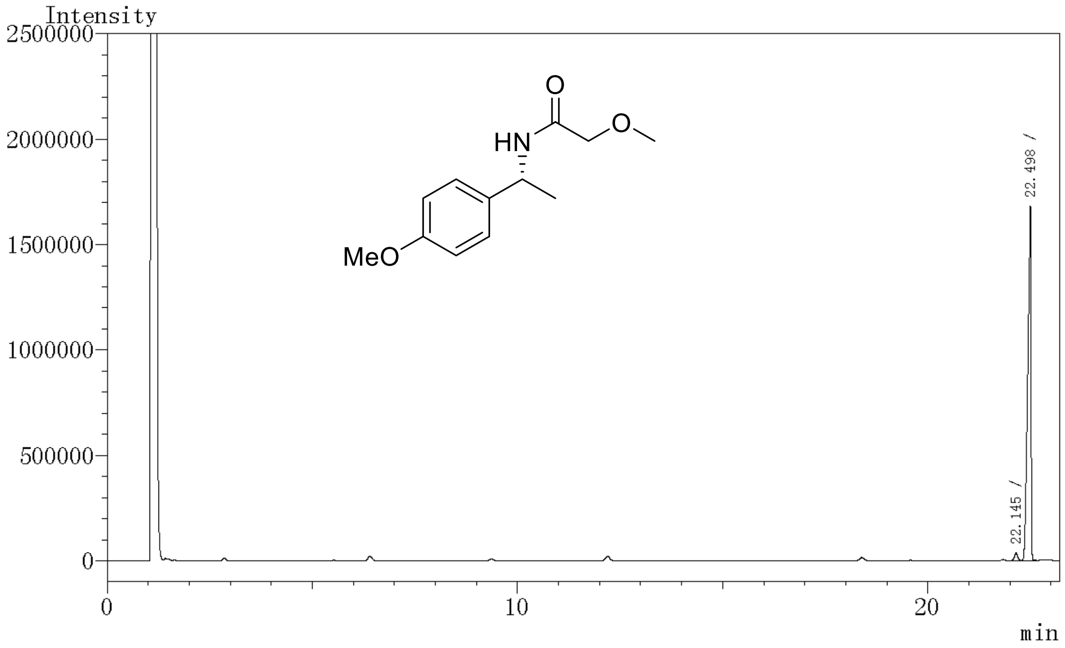

#	Time/min	Area	Height	Area%
1	22.145	181429	35833	1.964
2	22.498	9056503	1668824	98.036

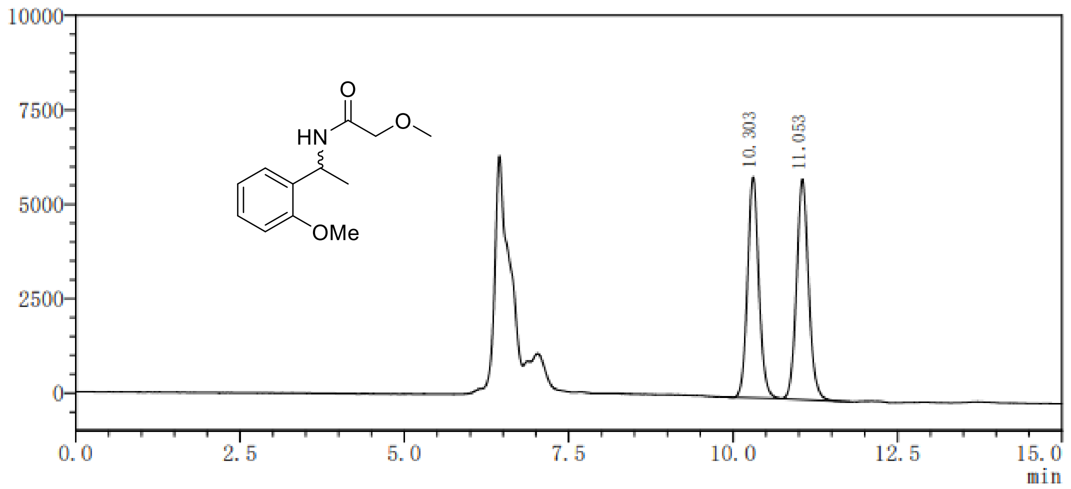

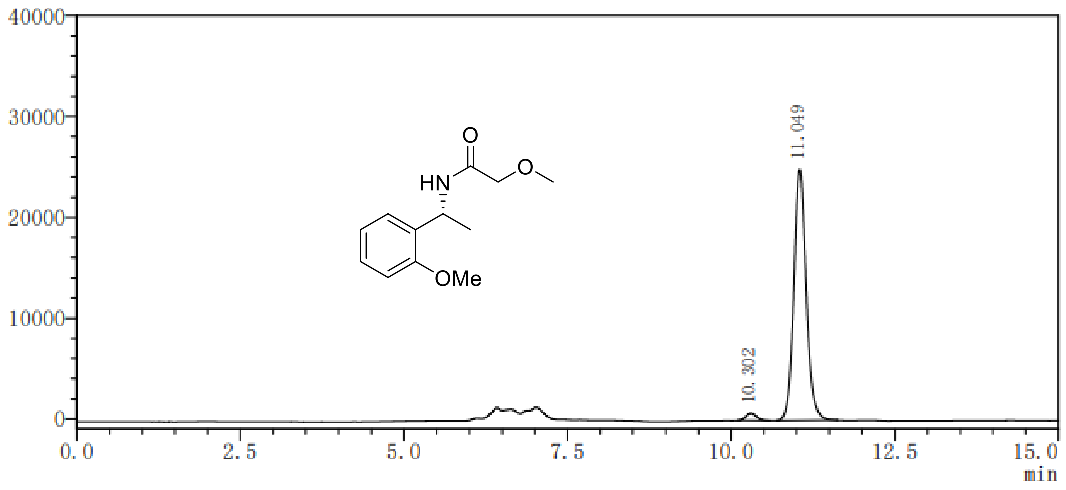

#	Time/min	Area	Height	Area%
1	10.302	8137	729	2.508%
2	11.049	328224	24915	97.492%

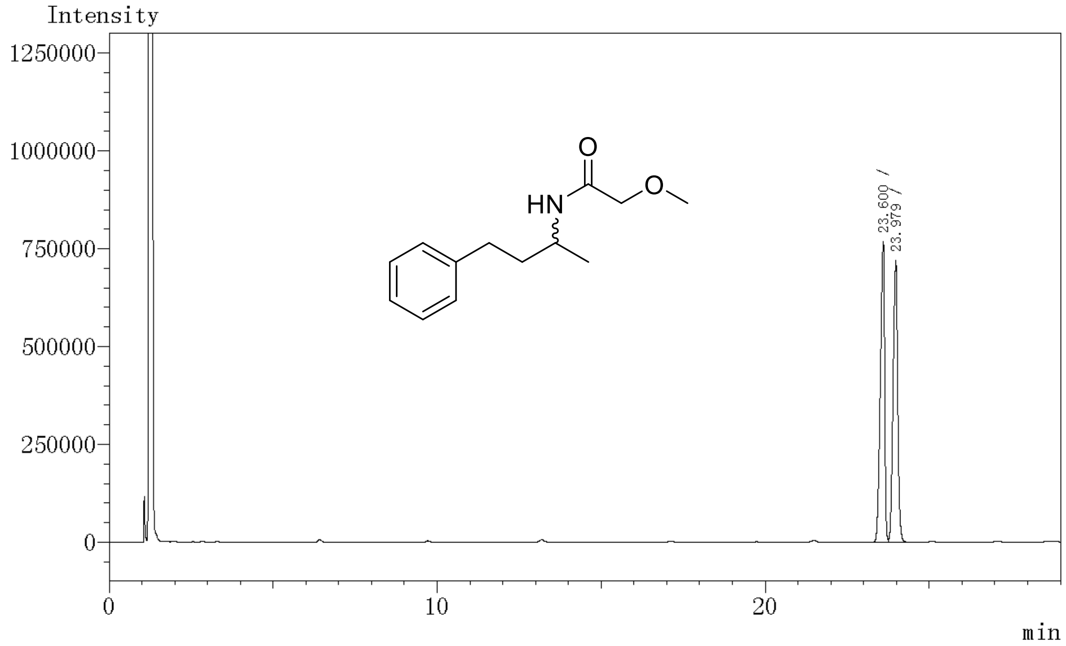

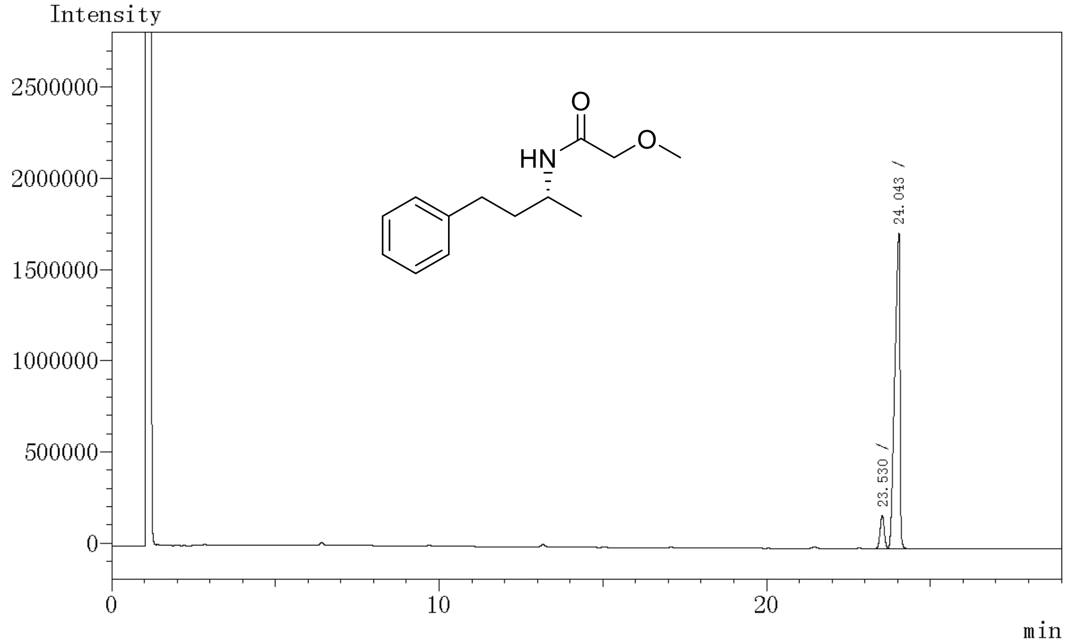

#	Time/min	Area	Height	Area%
1	23.530	1621783	183470	7.950
2	24.043	18777374	1730770	92.050

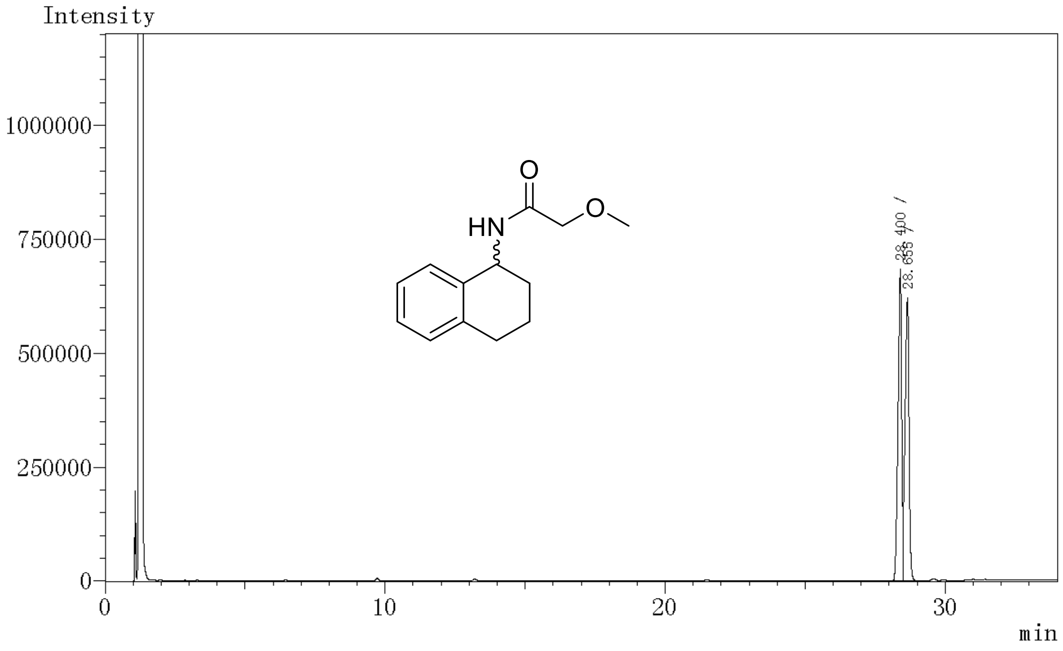

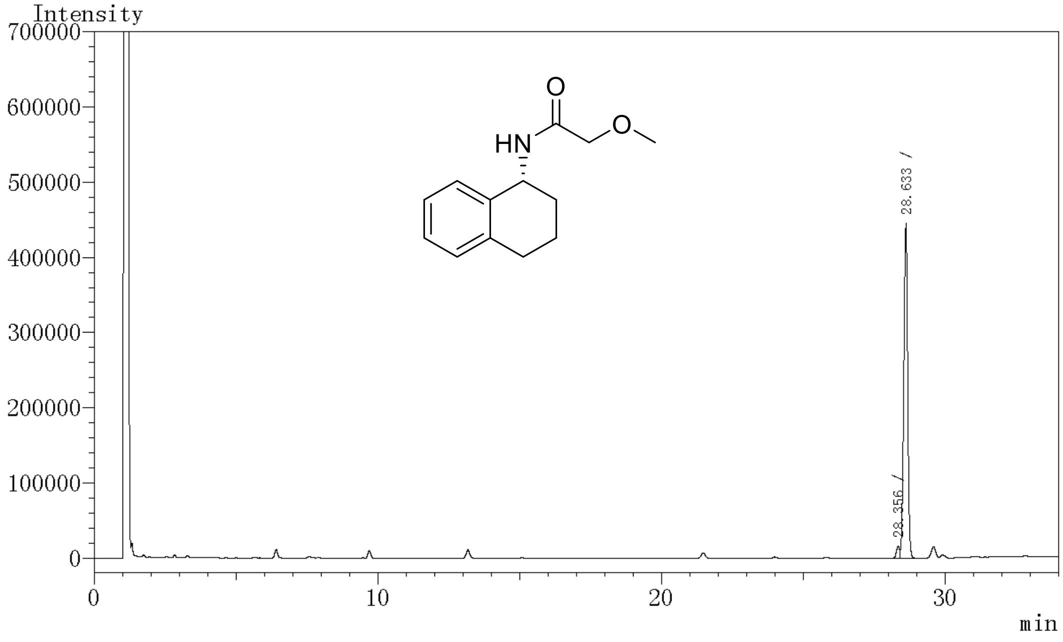

#	Time/min	Area	Height	Area%
1	28.356	121581	15744	2.792
2	28.633	4232868	445639	97.208

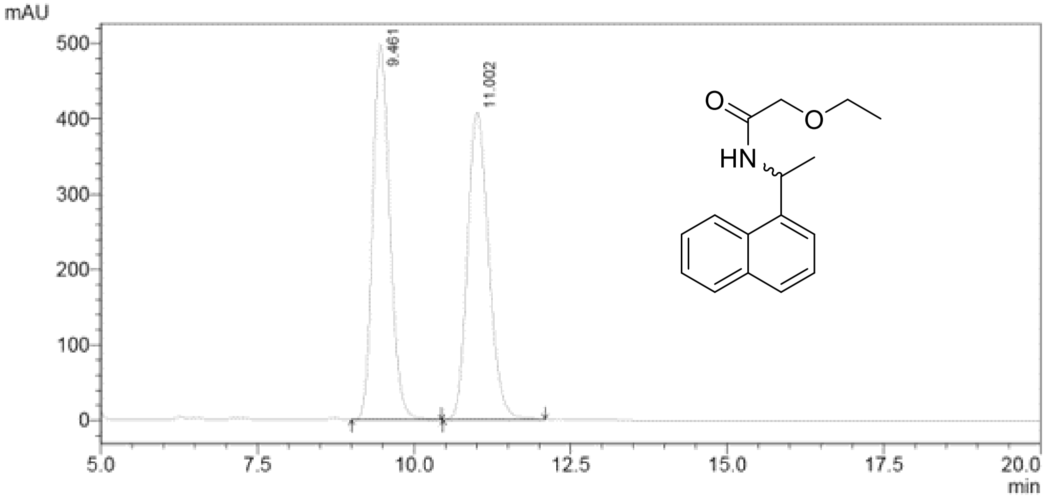

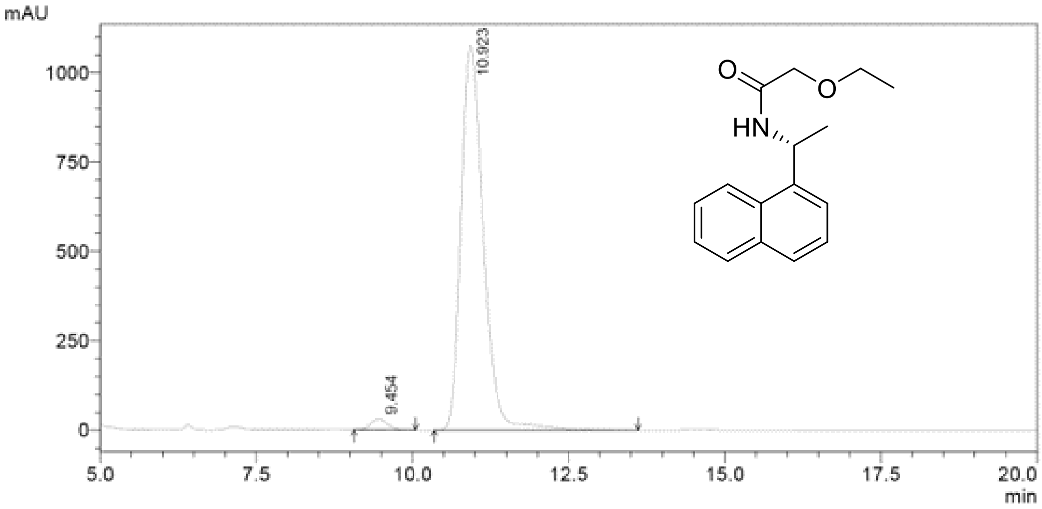

#	Time/min	Area	Height	Area%
1	9.454	560360	30013	2.713
2	10.923	26826935	1076078	97.287

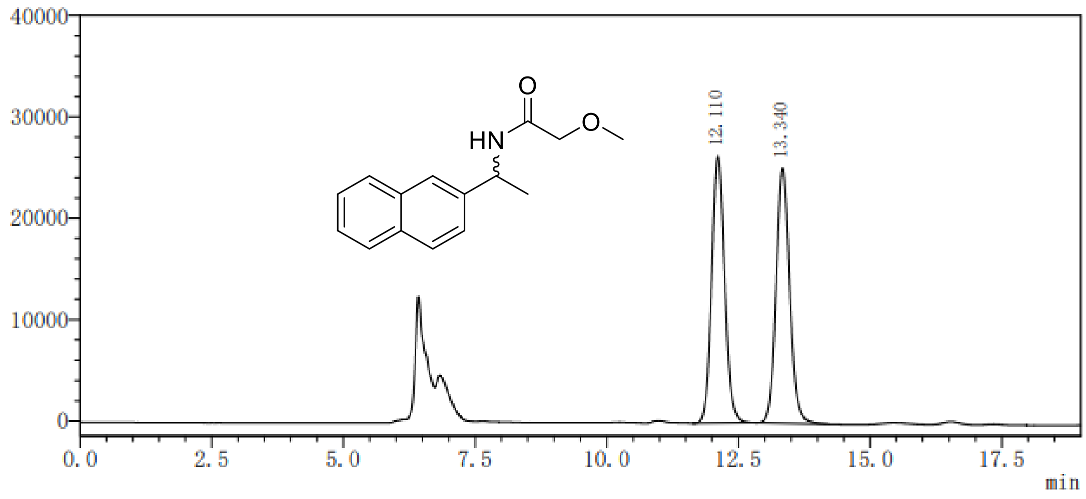

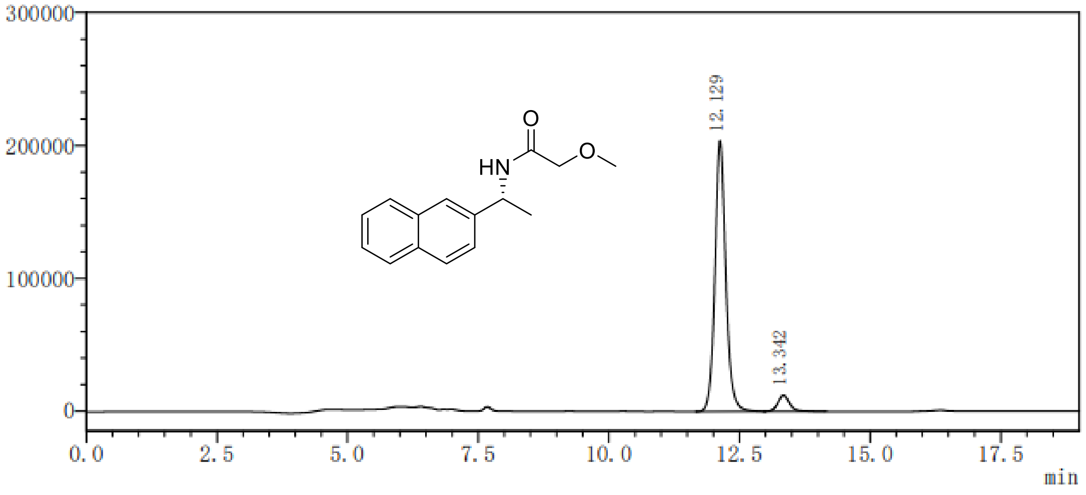

#	Time/min	Area	Height	Area%
1	12.129	2951836	203852	93.729%
2	13.342	197491	12297	6.271%

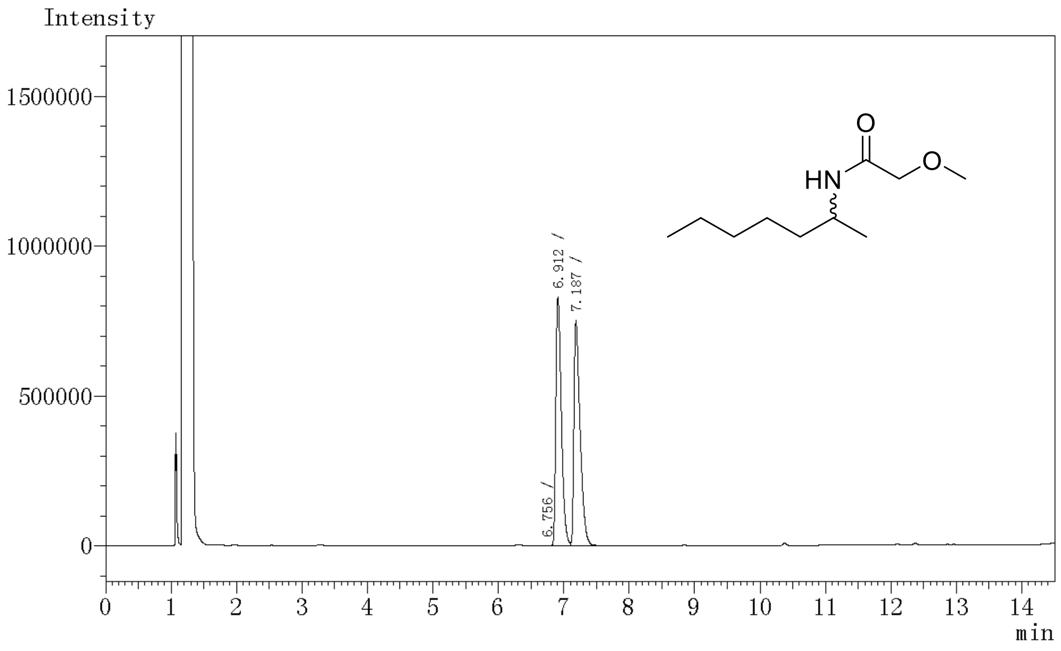

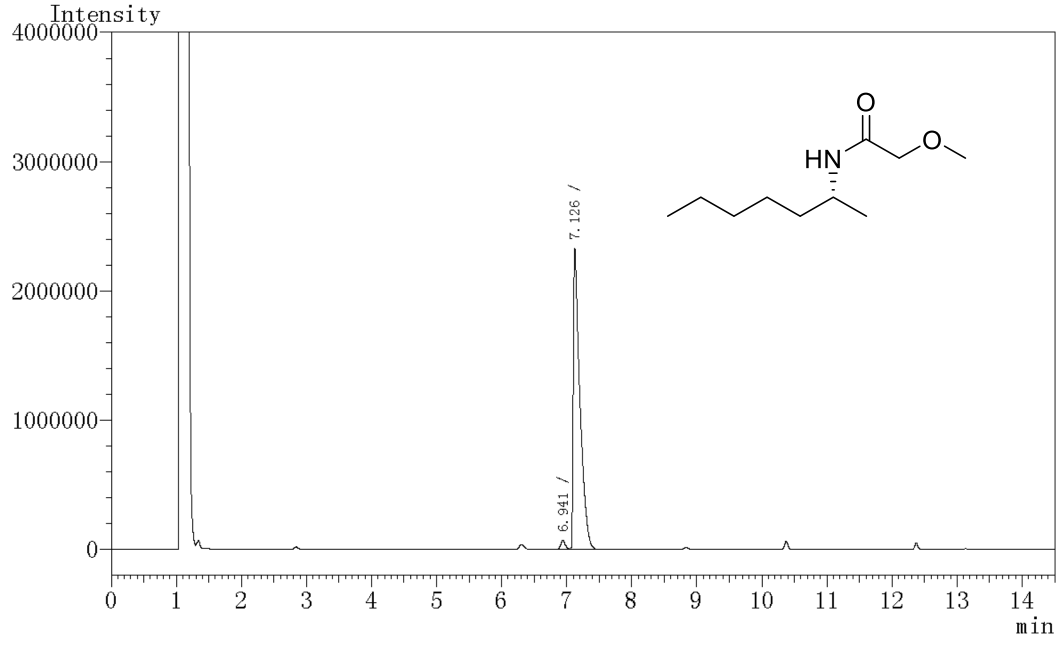

#	Time/min	Area	Height	Area%
1	6.941	356527	72943	2.122
2	7.126	16447498	2322913	97.878

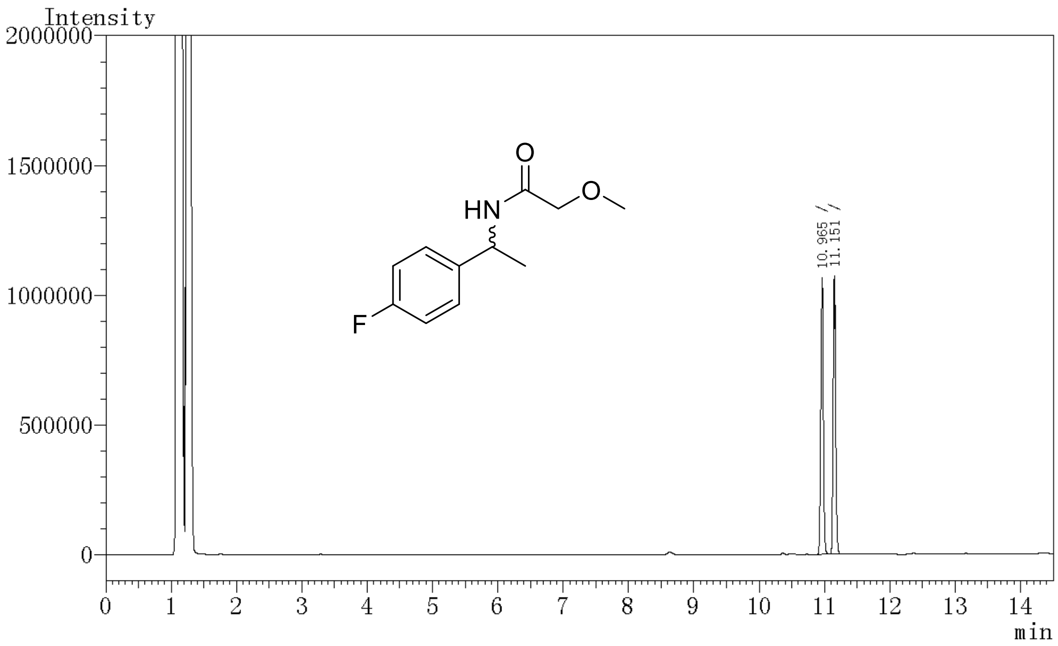

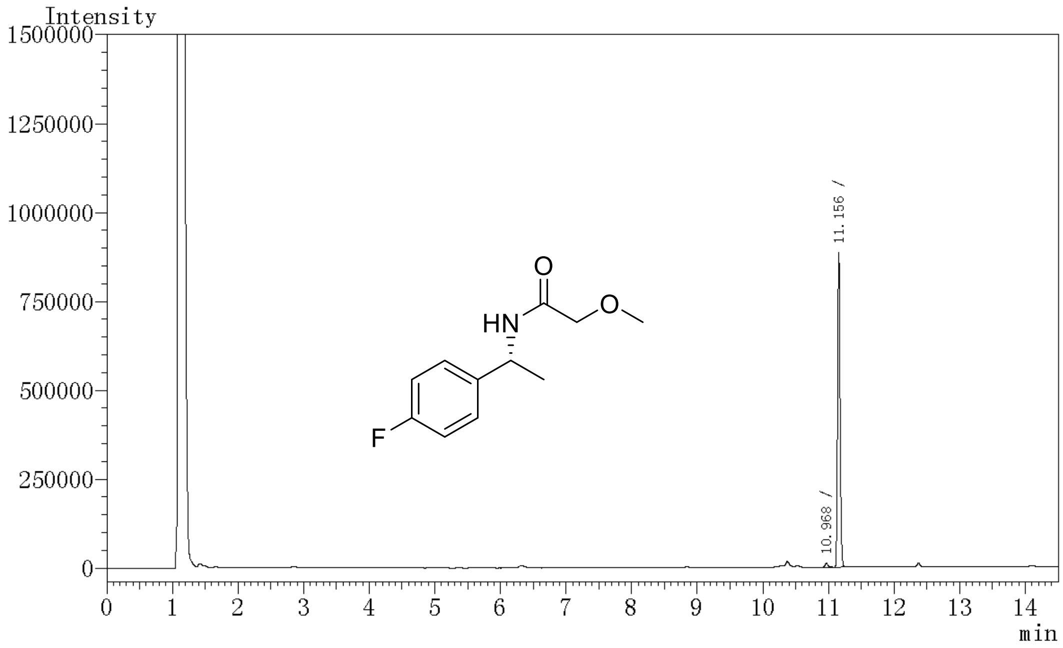

#	Time/min	Area	Height	Area%
1	10.968	39477	11680	1.682
2	11.156	2307196	881160	98.318

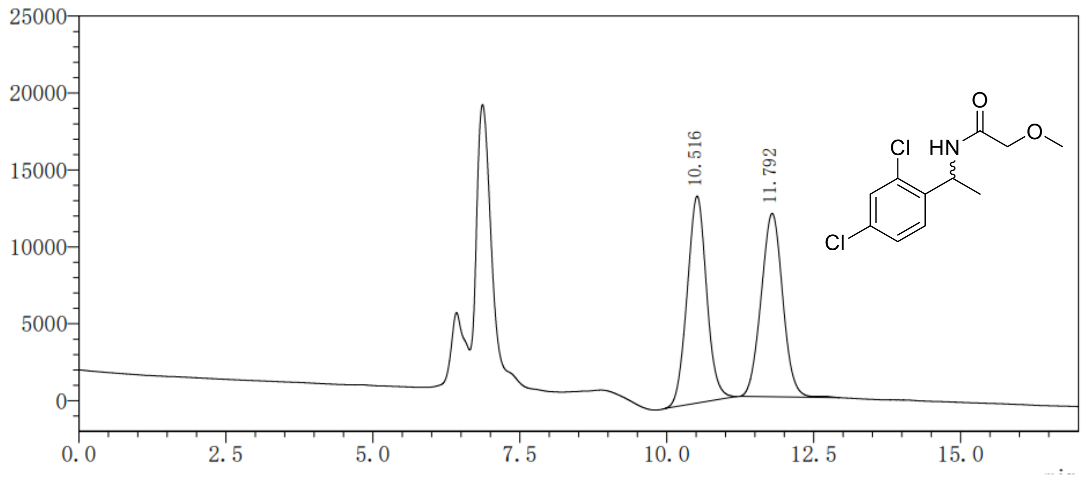

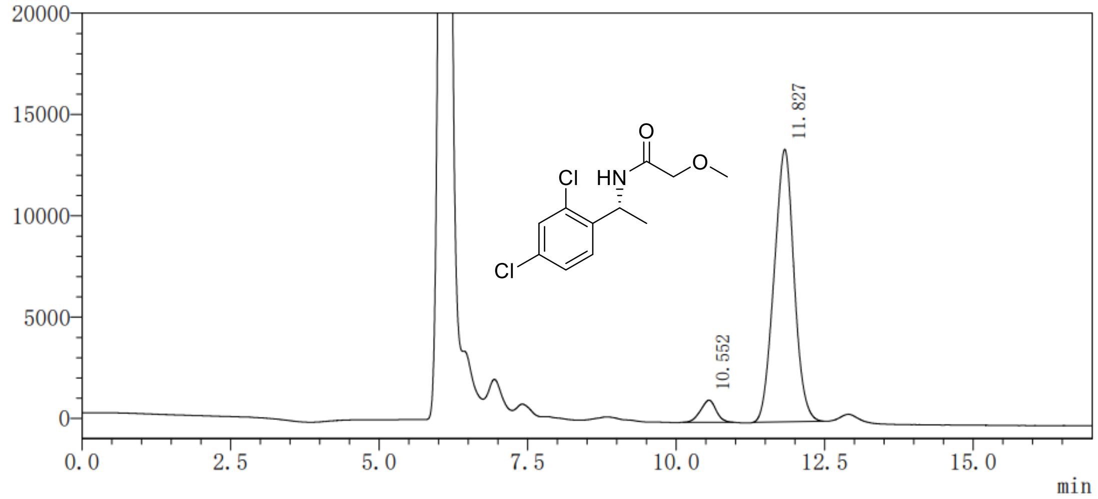

#	Time/min	Area	Height	Area%
1	10.552	19929	1095	5.967%
2	11.827	314040	13445	94.033%

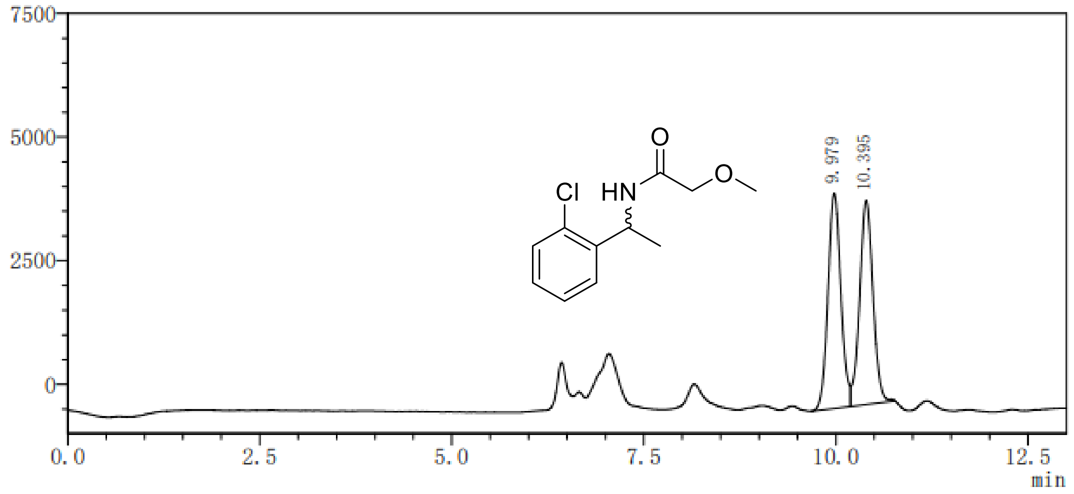

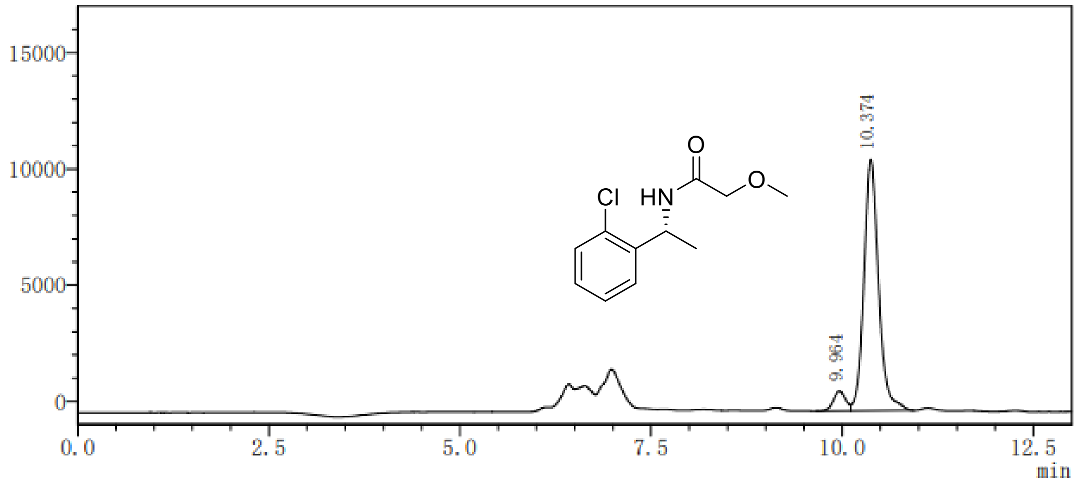

#	Time/min	Area	Height	Area%
1	9.964	9488	855	6.167%
2	10.374	144360	10819	93.833%

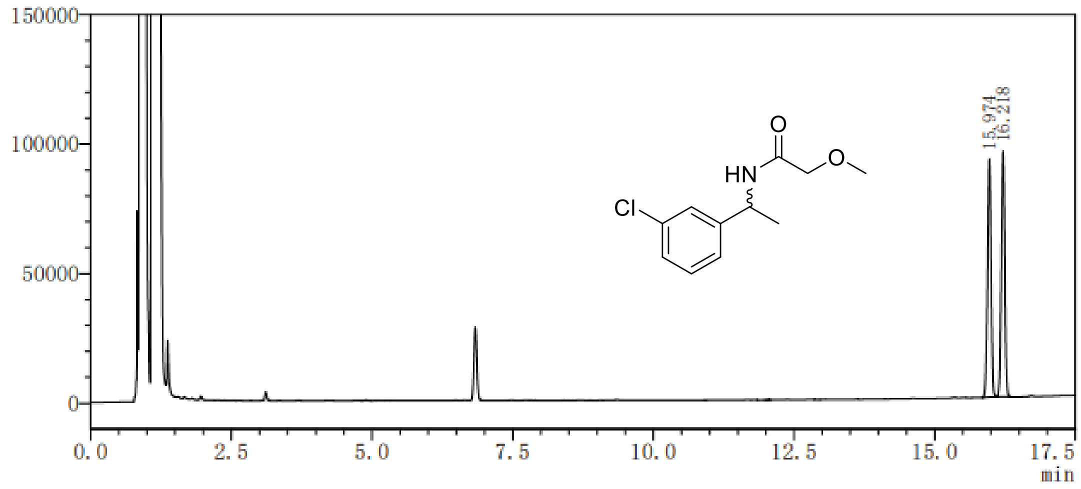

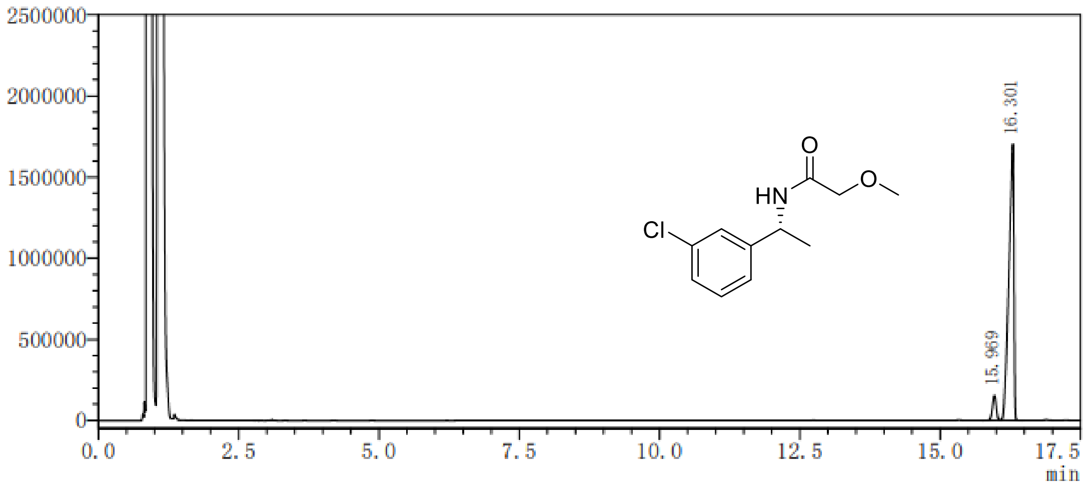

#	Time/min	Area	Height	Area%
1	15.969	715727	153729	6.394%
2	16.301	10477334	1697163	93.606%

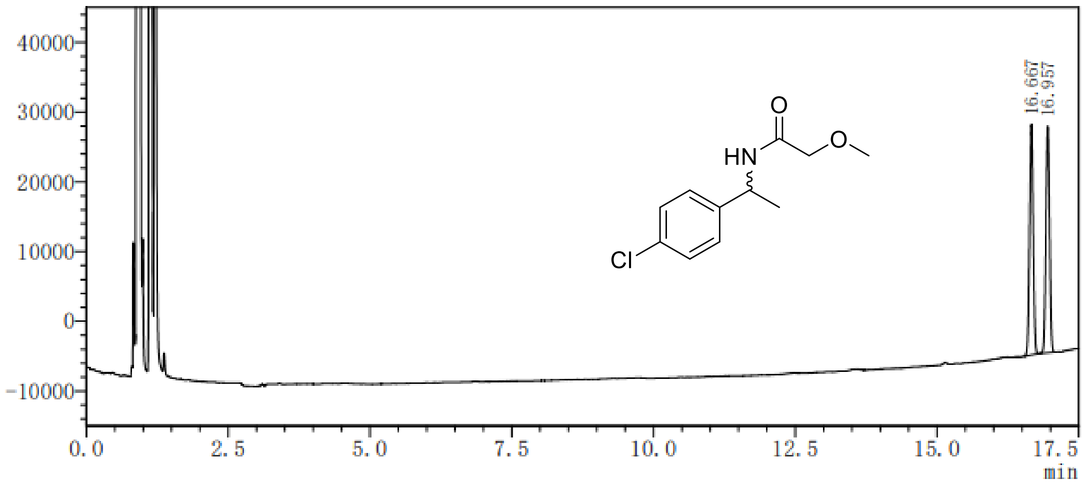

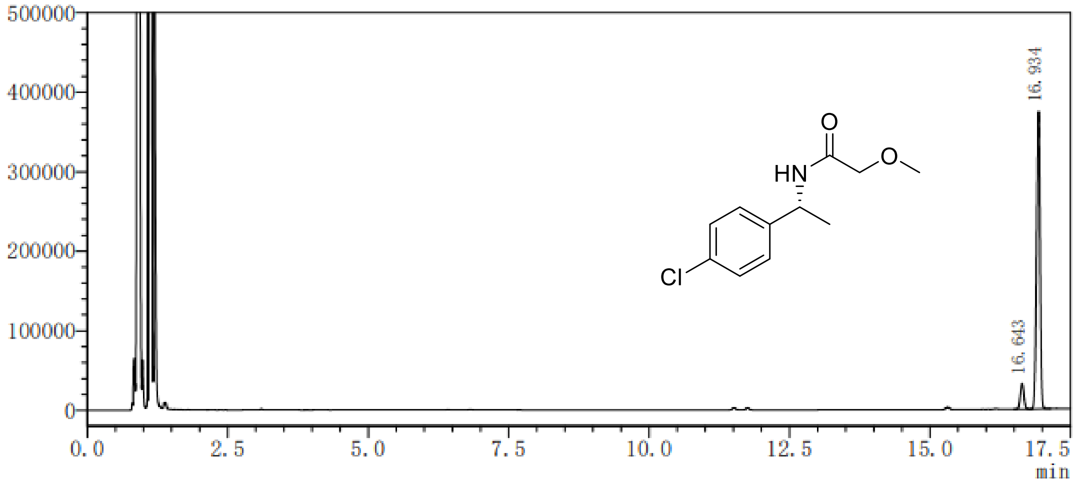

#	Time/min	Area	Height	Area%
1	16.643	146739	32294	8.418%
2	16.934	1596352	372901	91.582%

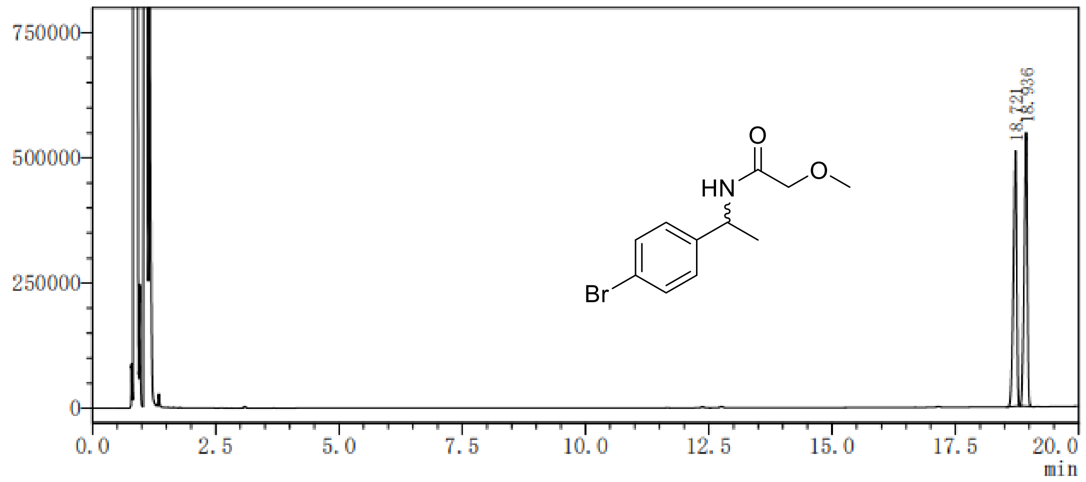

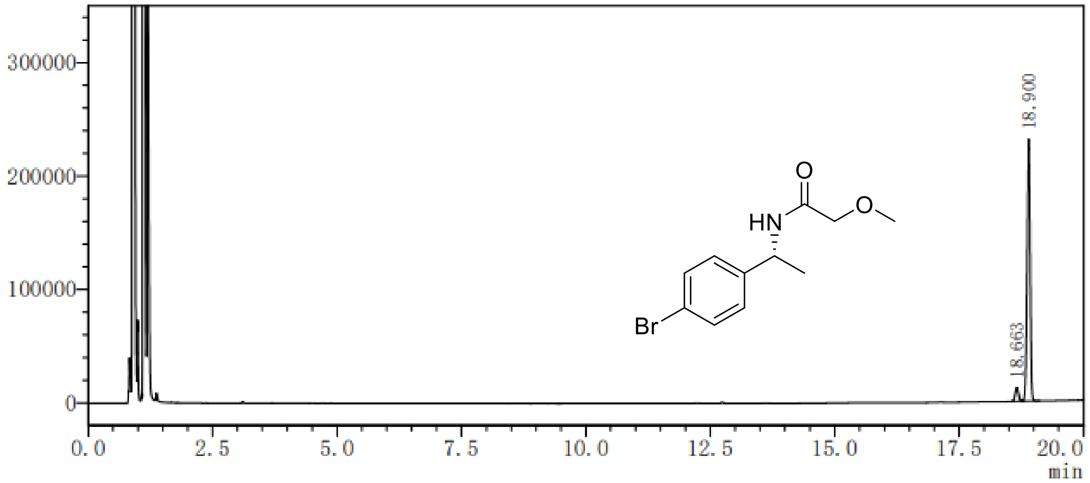

#	Time/min	Area	Height	Area%
1	18.663	54761	11954	5.071%
2	18.900	1025050	231017	94.929%

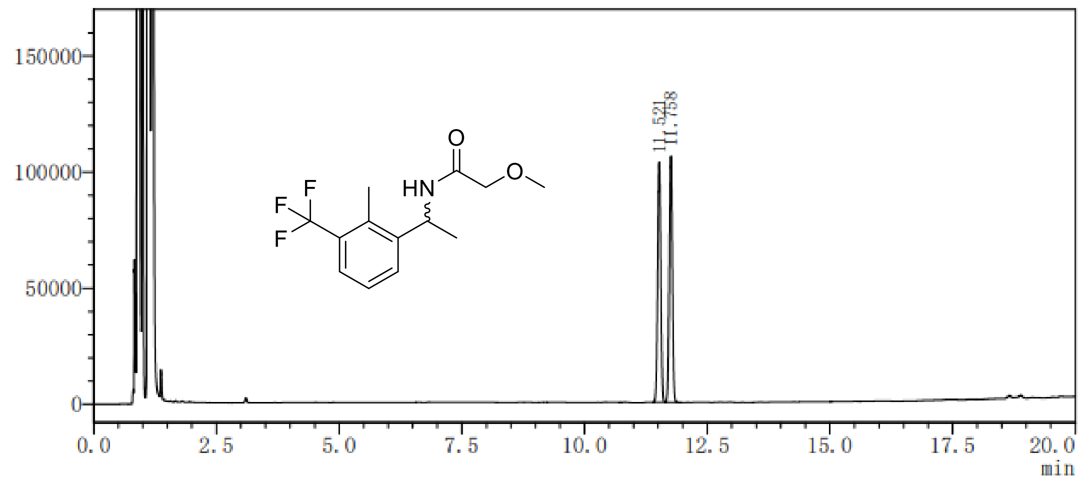

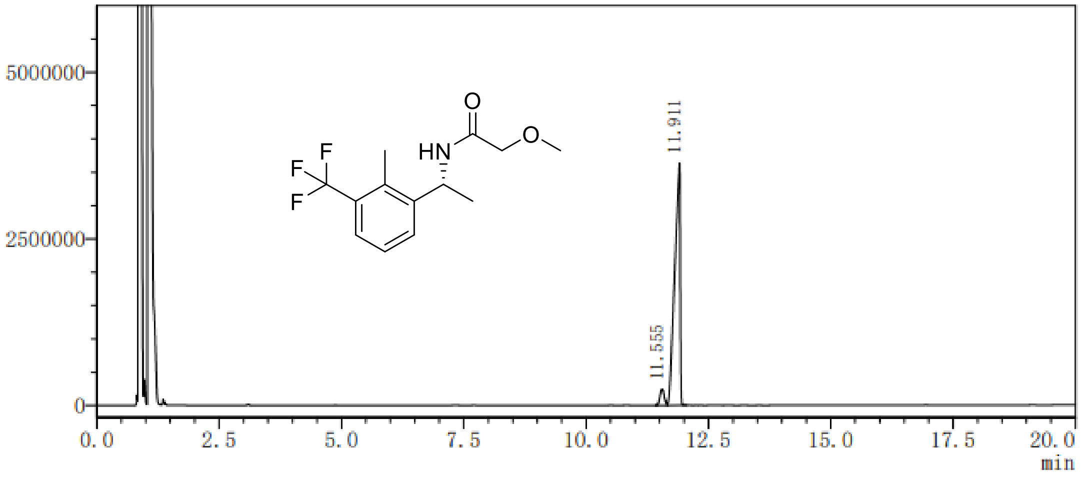

#	Time/min	Area	Height	Area%
1	11.555	1492153	242468	5.055%
2	11.911	28026963	3630025	94.945%

6. ^{1}H NMR spectra of compounds

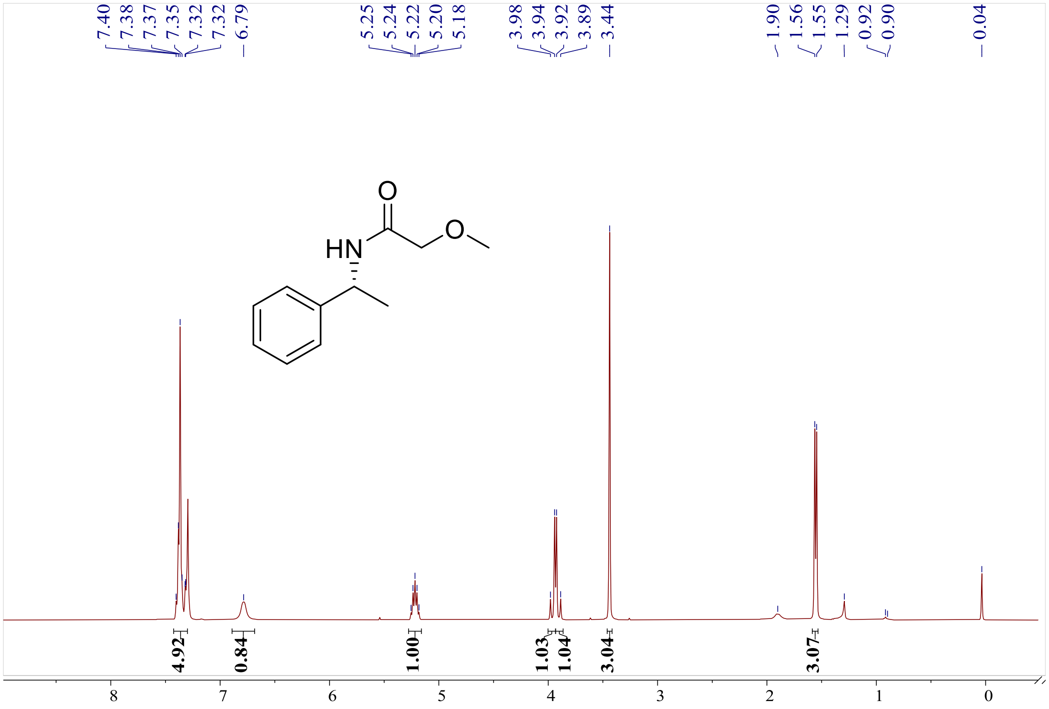

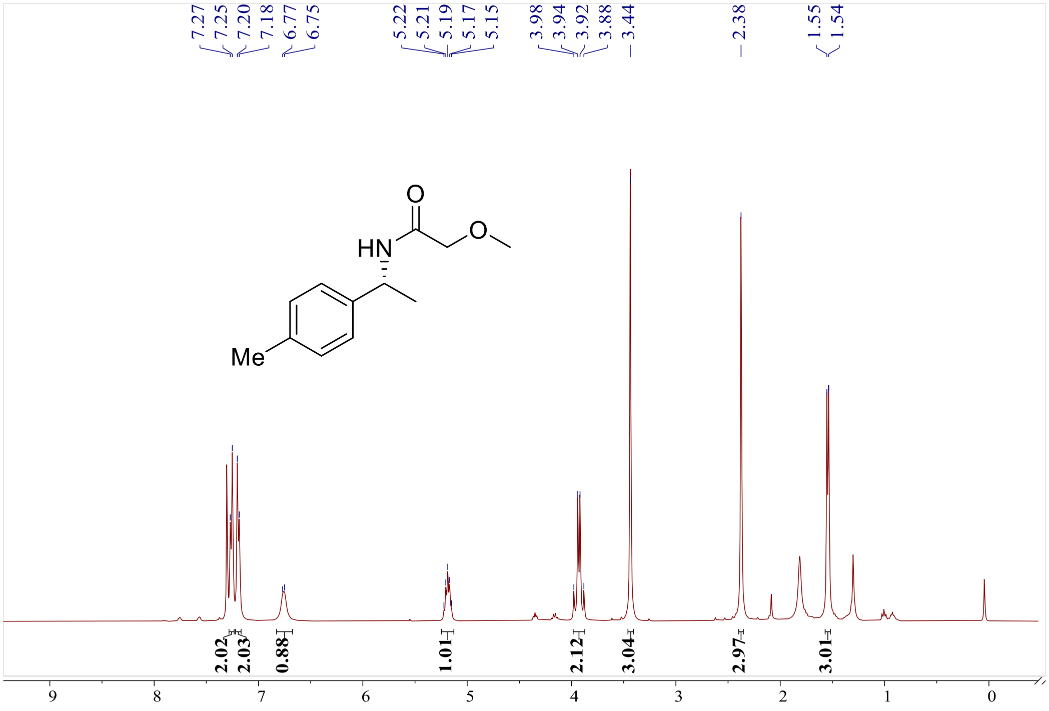

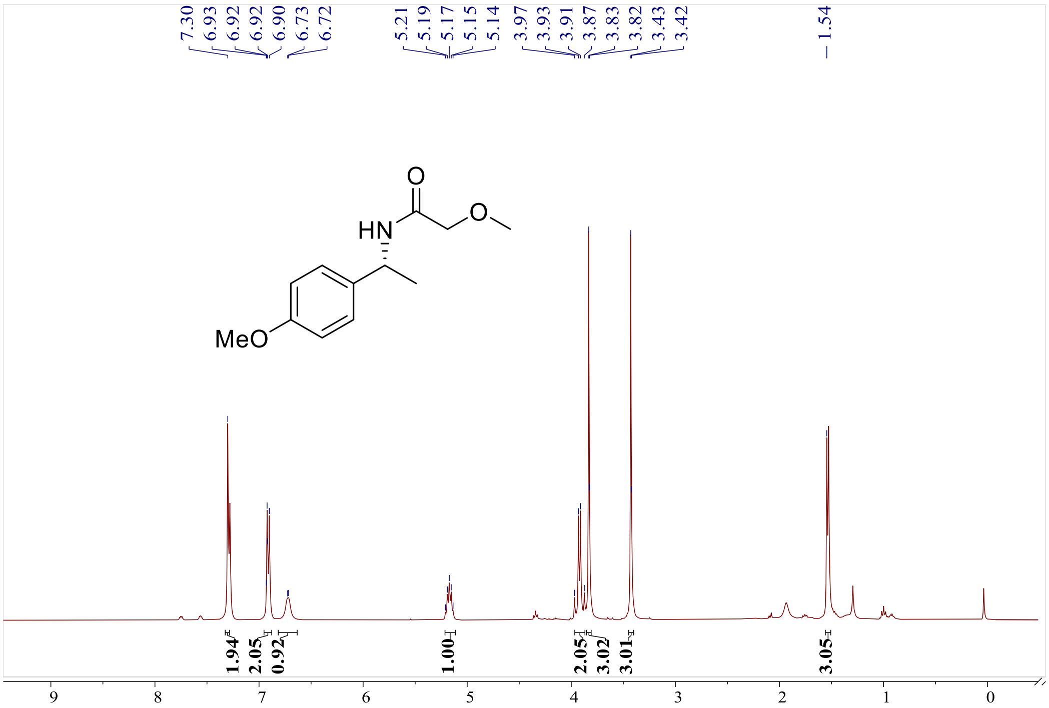
7.30
6.93
6.92
6.92
6.90
6.73
6.72
5.21
5.19
5.17
5.15
5.14
3.97
3.93
3.91
3.87
3.83
3.82
3.43
3.42
1.54
MeO
HN
O
O
1.94
2.05
0.92
1.00
2.05
3.02
3.01
3.05
9
8
7
6
5
4
3
2
1
0

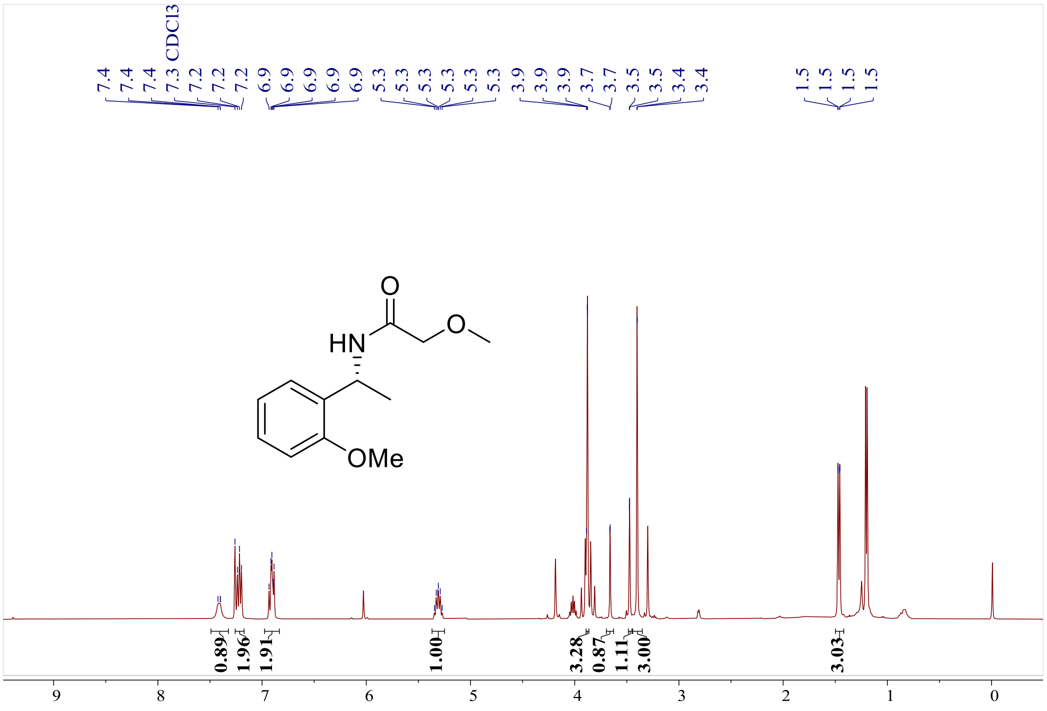
7.4
7.4
7.4
7.3 CDCl3
7.2
7.2
7.2
6.9
6.9
6.9
6.9
6.9
5.3
5.3
5.3
5.3
5.3
5.3
3.9
3.9
3.9
3.7
3.7
3.5
3.5
3.4
3.4
1.5
1.5
1.5
1.5
HN
O
O
OMe
0.89
1.96
1.91
1.00
3.28
0.87
1.11
3.00
3.03
9
8
7
6
5
4
3
2
1
0

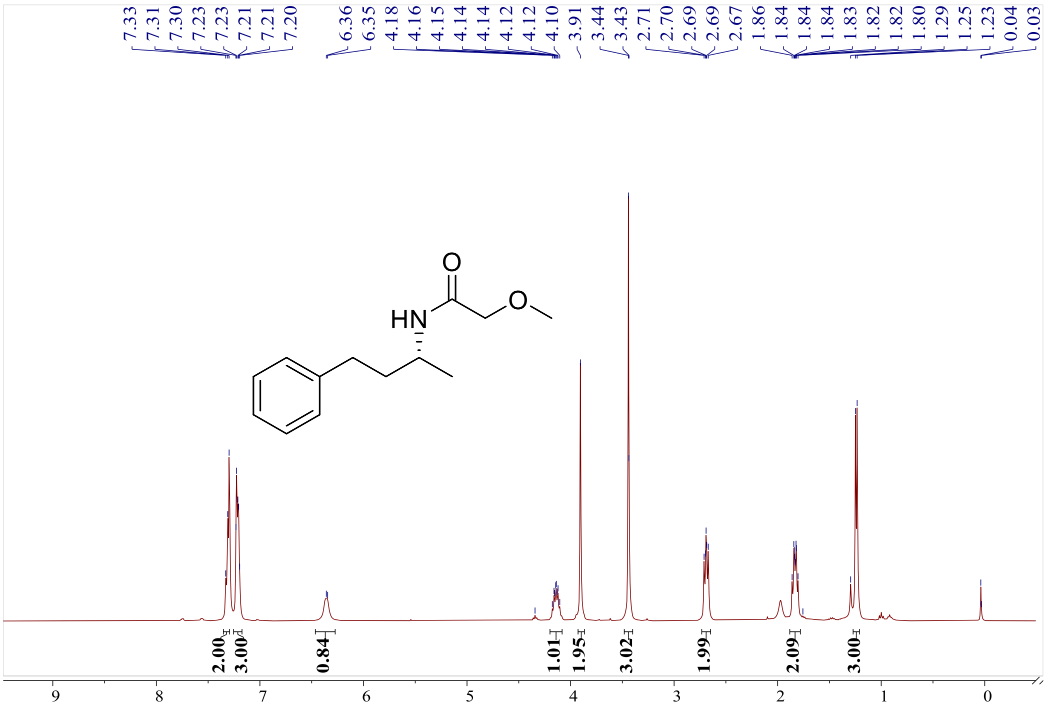
7.33
7.31
7.30
7.23
7.23
7.21
7.21
7.20
6.36
6.35
4.18
4.16
4.15
4.14
4.14
4.12
4.12
4.10
3.91
3.44
3.43
2.71
2.70
2.69
2.69
2.67
1.86
1.84
1.84
1.84
1.83
1.82
1.82
1.80
1.29
1.25
1.23
0.04
0.03
O
HN
O
2.00
3.00
0.84
1.01
1.95
3.02
1.99
2.09
3.00
9
8
7
6
5
4
3
2
1
0

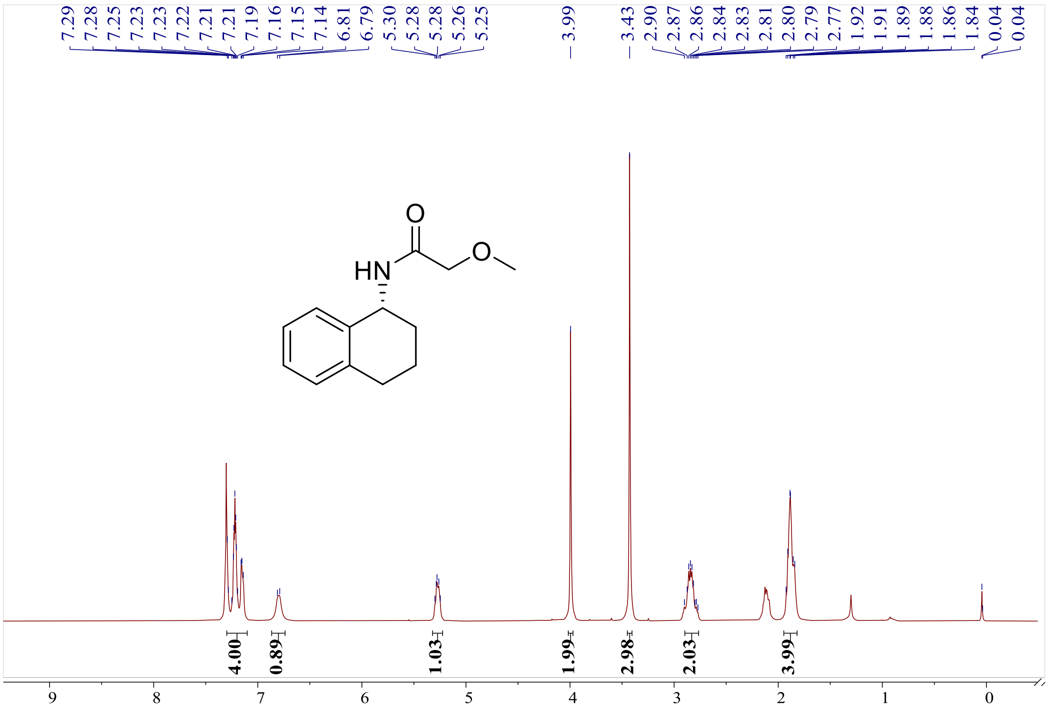
7.29
7.28
7.25
7.23
7.23
7.22
7.21
7.21
7.19
7.16
7.15
7.14
6.81
6.79
5.30
5.28
5.28
5.26
5.25
3.99
3.43
2.90
2.87
2.86
2.84
2.83
2.81
2.80
2.79
2.77
1.92
1.91
1.89
1.88
1.86
1.84
0.04
0.04
O
HN
O
4.00
0.89
1.03
1.99
2.98
2.03
3.99
9
8
7
6
5
4
3
2
1
0

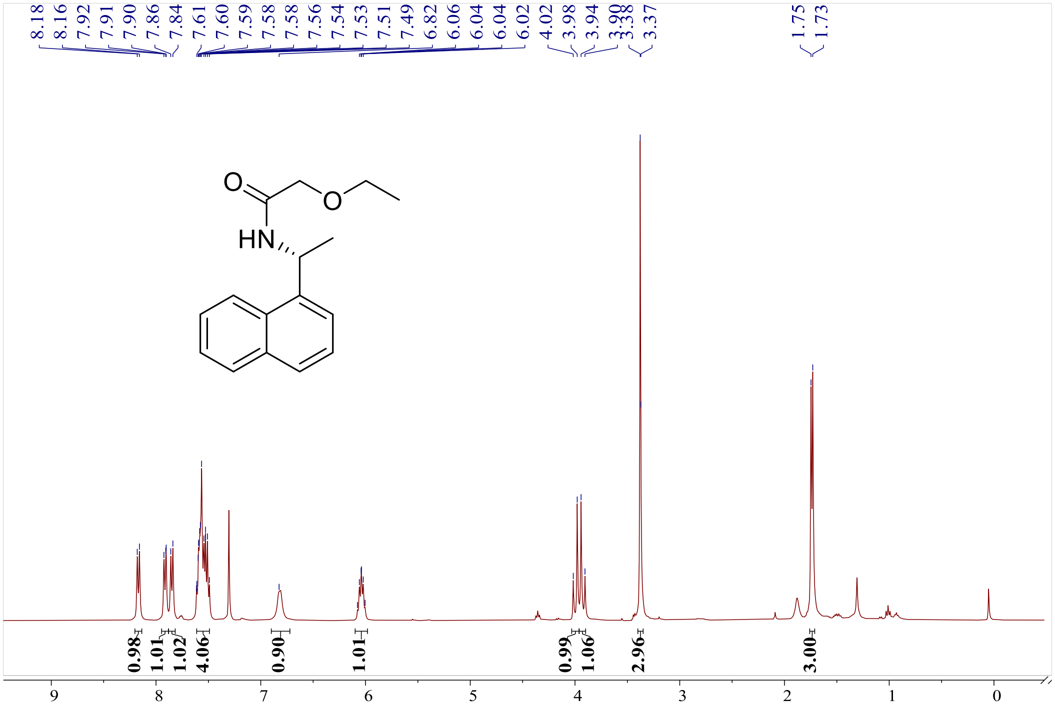
8.18 8.16 7.92 7.91 7.90 7.86 7.84 7.61 7.60 7.59 7.58 7.58 7.56 7.54 7.53 7.51 7.49 6.82 6.06 6.04 6.04 6.02 4.02 3.98 3.94 3.90 3.38 3.37 1.75 1.73
O
HN
O
0.98 1.01 1.02 4.06 0.90 1.01 0.99 1.06 2.96 3.00
9 8 7 6 5 4 3 2 1 0

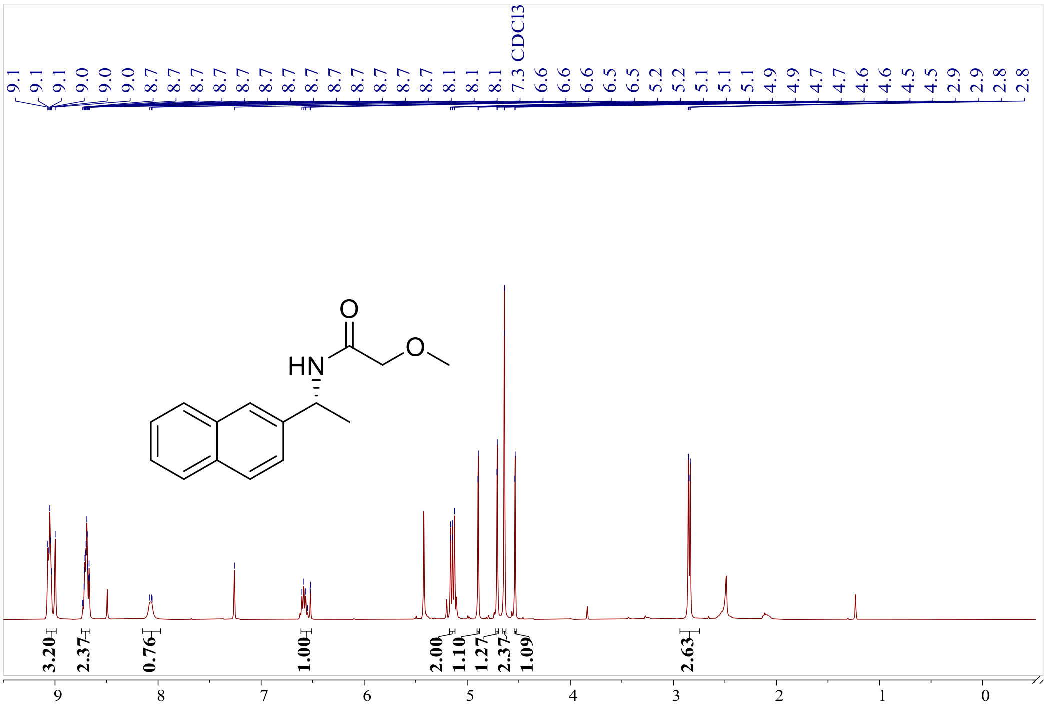
9.1 9.1 9.1 9.0 9.0 8.7 8.7 8.7 8.7 8.7 8.7 8.7 8.7 8.7 8.7 8.7 8.7 8.1 8.1 8.1 7.3 CDCl3 6.6 6.6 6.6 6.5 6.5 5.2 5.2 5.1 5.1 5.1 4.9 4.9 4.7 4.7 4.6 4.6 4.5 4.5 2.9 2.9 2.8 2.8
O
HN
O
3.20 2.37 0.76 1.00 2.00 1.10 1.27 2.37 1.09 2.63
9 8 7 6 5 4 3 2 1 0

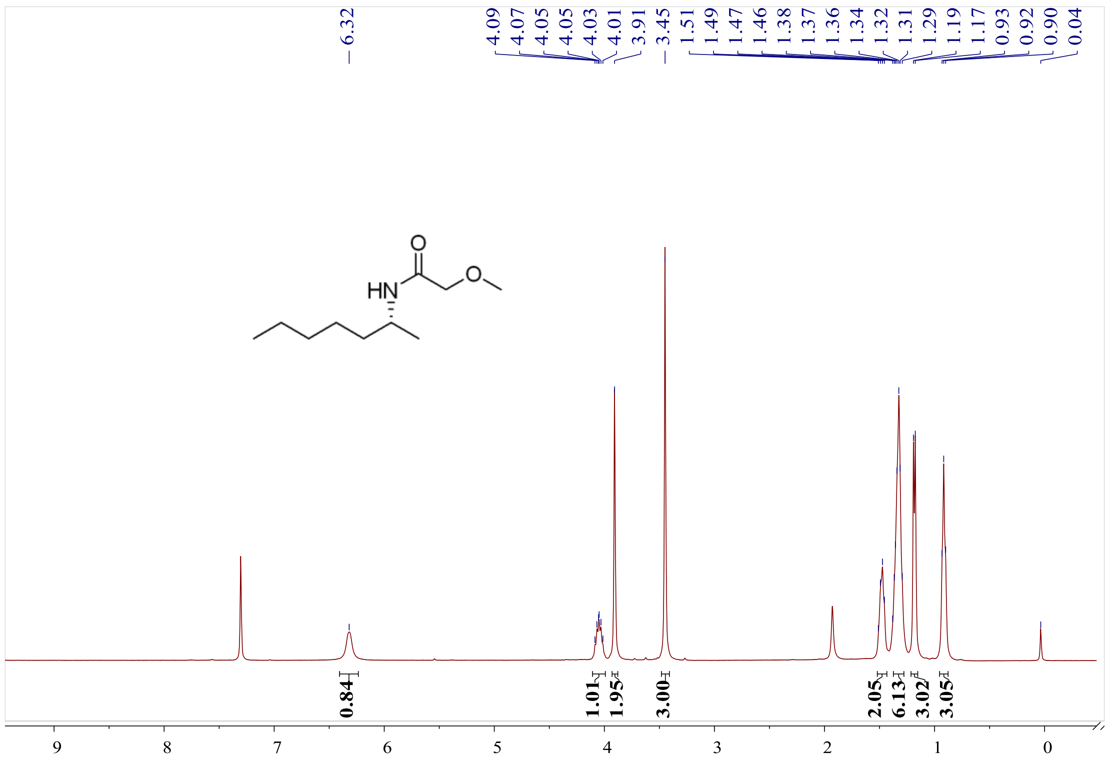
6.32
4.09 4.07 4.05 4.05 4.03 4.01 3.91 3.45 1.51 1.49 1.47 1.46 1.38 1.37 1.36 1.34 1.32 1.31 1.29 1.19 1.17 0.93 0.92 0.90 0.04
HN
O
0.84
1.01 1.95
3.00
2.05 6.13 3.02 3.05
9 8 7 6 5 4 3 2 1 0

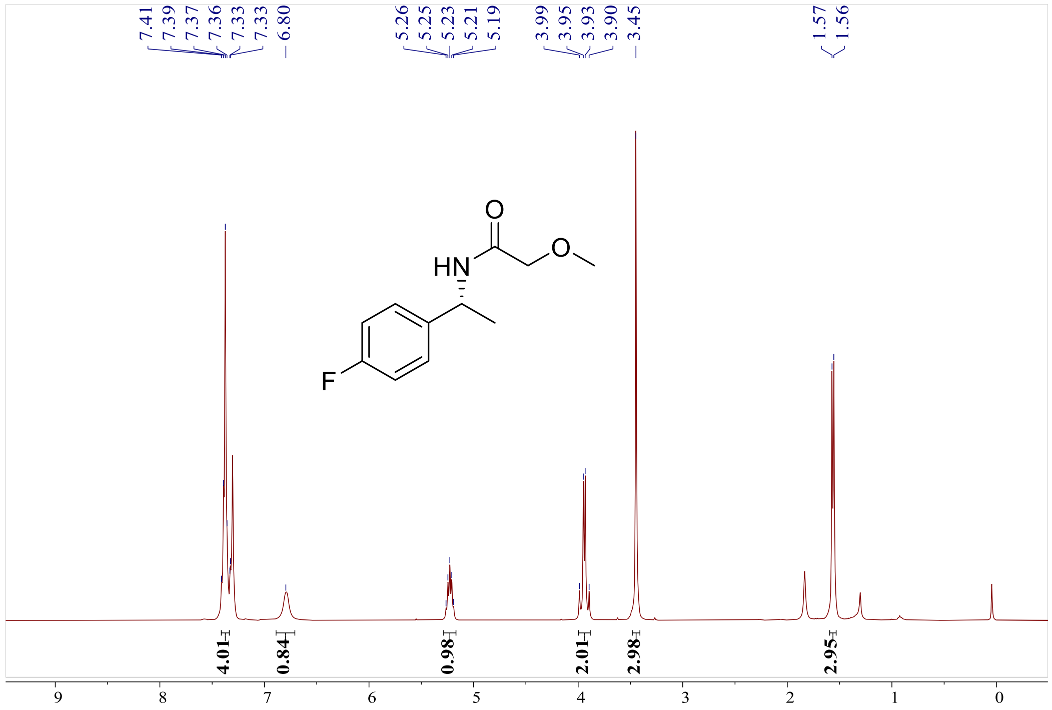
7.41 7.39 7.37 7.36 7.33 7.33 6.80
5.26 5.25 5.23 5.21 5.19
3.99 3.95 3.93 3.90 3.45
1.57 1.56
HN
O
F
4.01 0.84
0.98
2.01 2.98
2.95
9 8 7 6 5 4 3 2 1 0

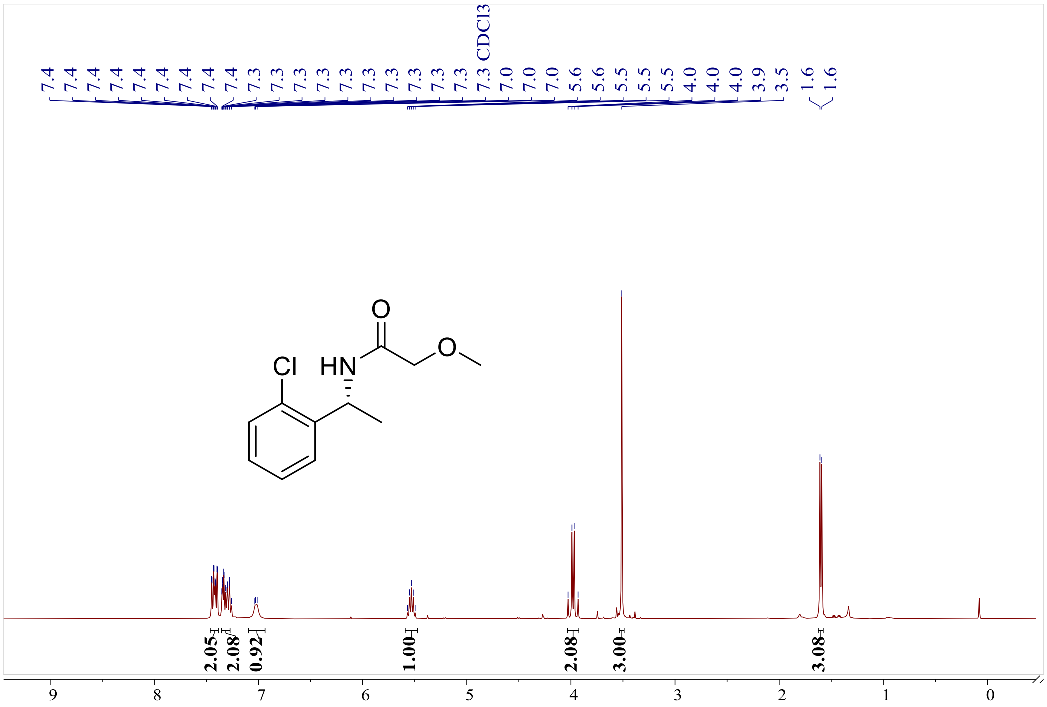
7.4
7.4
7.4
7.4
7.4
7.4
7.4
7.4
7.3
7.3
7.3
7.3
7.3
7.3
7.3
7.3
7.3
7.3 CDCl3
7.0
7.0
7.0
5.6
5.6
5.5
5.5
5.5
4.0
4.0
4.0
3.9
3.5
1.6
1.6
Cl
HN
O
O
2.05
2.08
0.92
1.00
2.08
3.00
3.08
9
8
7
6
5
4
3
2
1
0

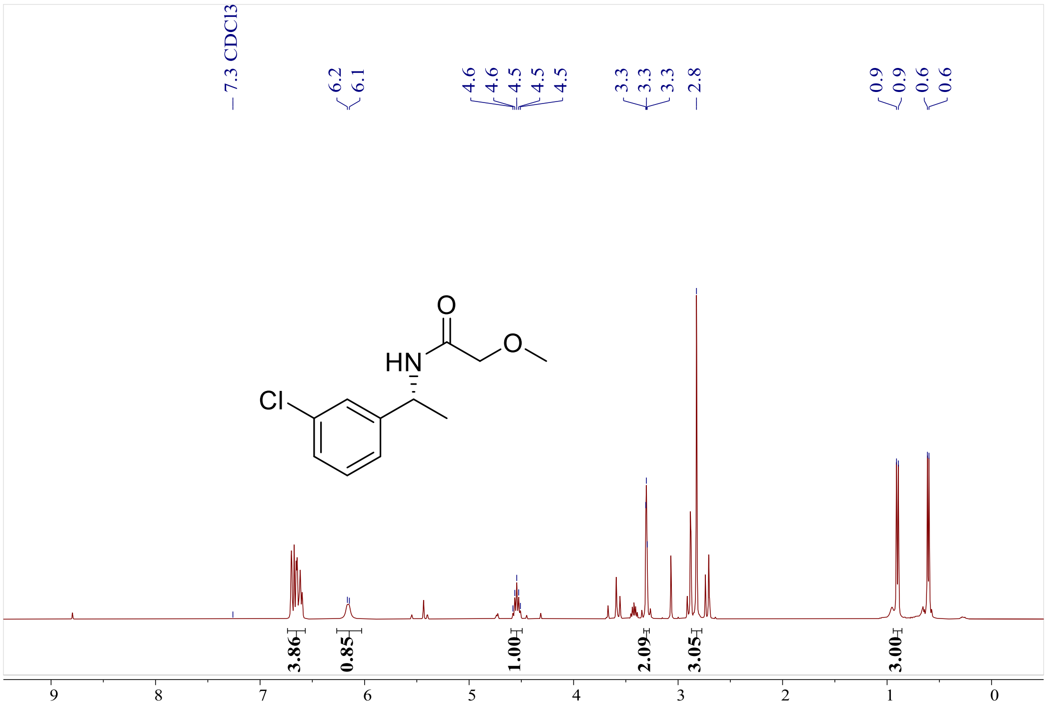
7.3 CDCl3
6.2
6.1
4.6
4.6
4.5
4.5
4.5
3.3
3.3
3.3
2.8
0.9
0.9
0.6
0.6
Cl
HN
O
O
3.86
0.85
1.00
2.09
3.05
3.00
9
8
7
6
5
4
3
2
1
0

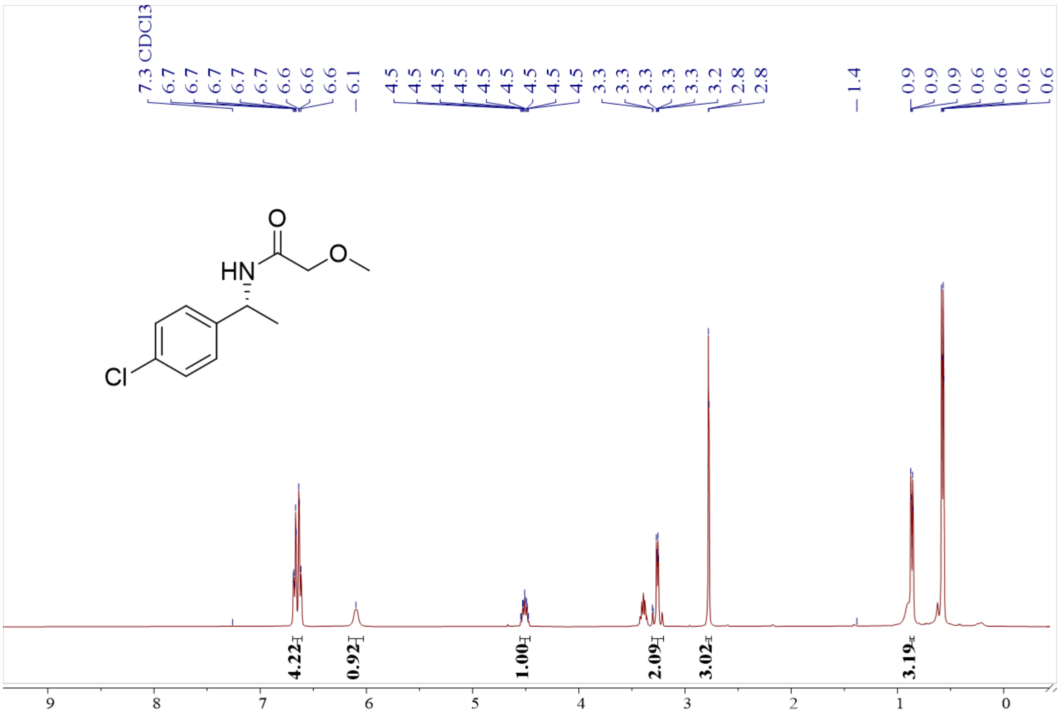
7.3 CDCl3
6.7
6.7
6.7
6.7
6.7
6.6
6.6
6.6
6.1
4.5
4.5
4.5
4.5
4.5
4.5
4.5
4.5
4.5
3.3
3.3
3.3
3.3
3.3
3.2
2.8
2.8
1.4
0.9
0.9
0.9
0.6
0.6
0.6
0.6
O
HN
O
Cl
4.22
0.92
1.00
2.09
3.02
3.19
9
8
7
6
5
4
3
2
1
0

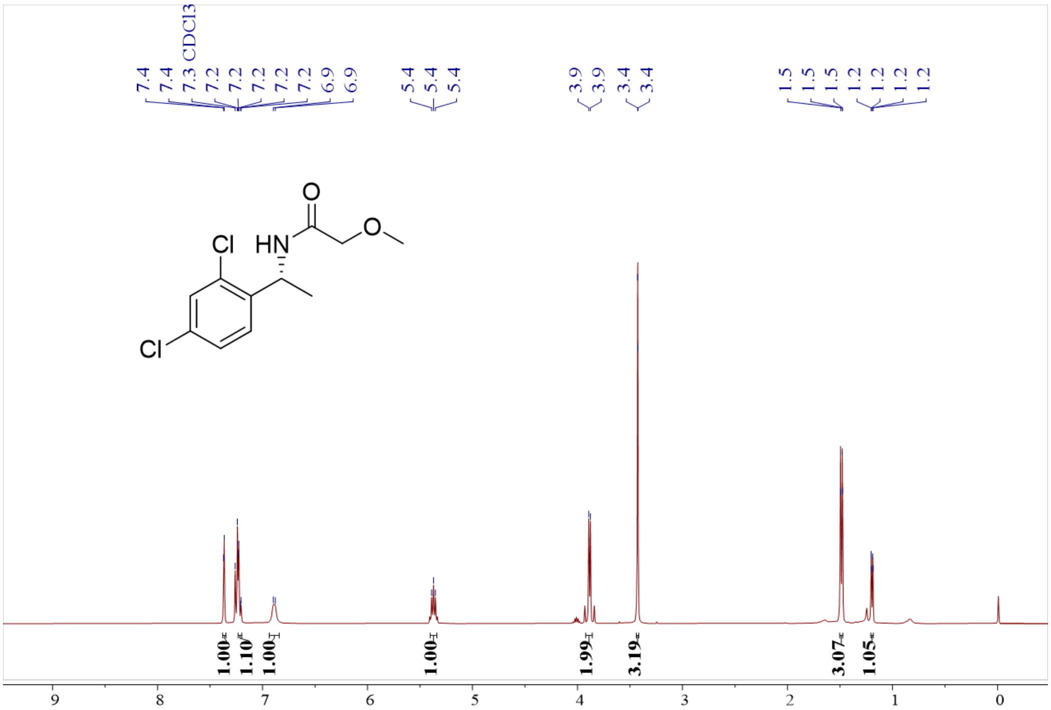
7.4
7.4
7.3 CDCl3
7.2
7.2
7.2
7.2
7.2
6.9
6.9
5.4
5.4
5.4
3.9
3.9
3.4
3.4
1.5
1.5
1.5
1.2
1.2
1.2
1.2
O
Cl
HN
O
Cl
1.00
1.10
1.00
1.00
1.99
3.19
3.07
1.05
9
8
7
6
5
4
3
2
1
0

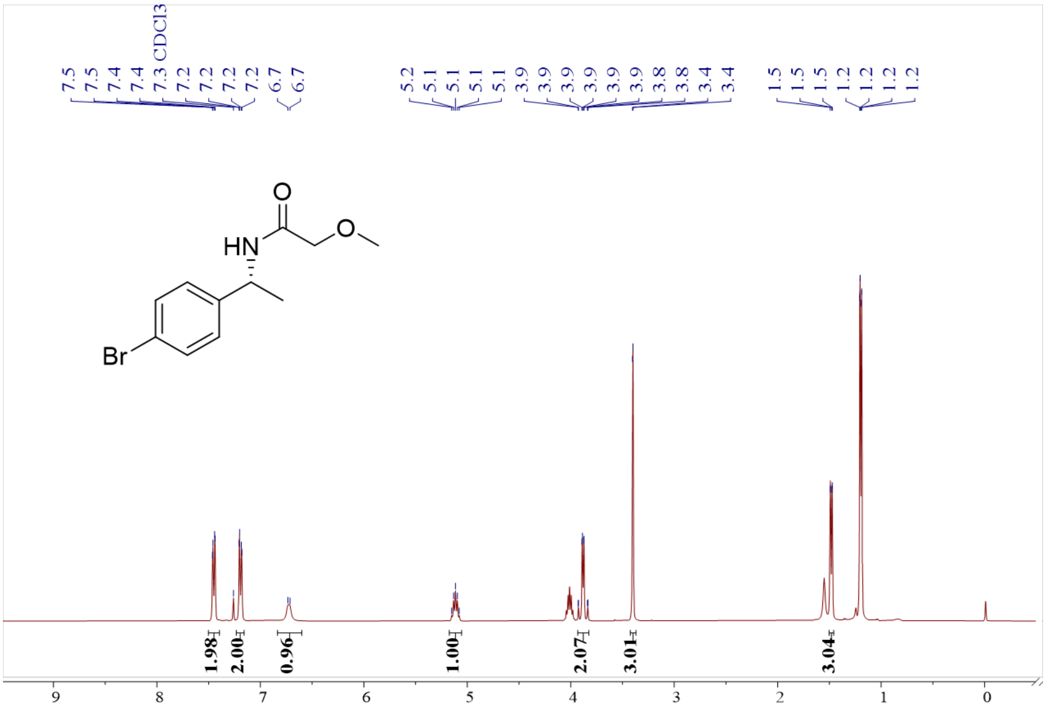
7.5
7.5
7.4
7.4
7.3 CDCl3
7.2
7.2
7.2
7.2
6.7
6.7
5.2
5.1
5.1
5.1
5.1
3.9
3.9
3.9
3.9
3.9
3.9
3.8
3.8
3.4
3.4
1.5
1.5
1.5
1.2
1.2
1.2
1.2
O
HN
O
Br
1.98
2.00
0.96
1.00
2.07
3.01
3.04
9
8
7
6
5
4
3
2
1
0

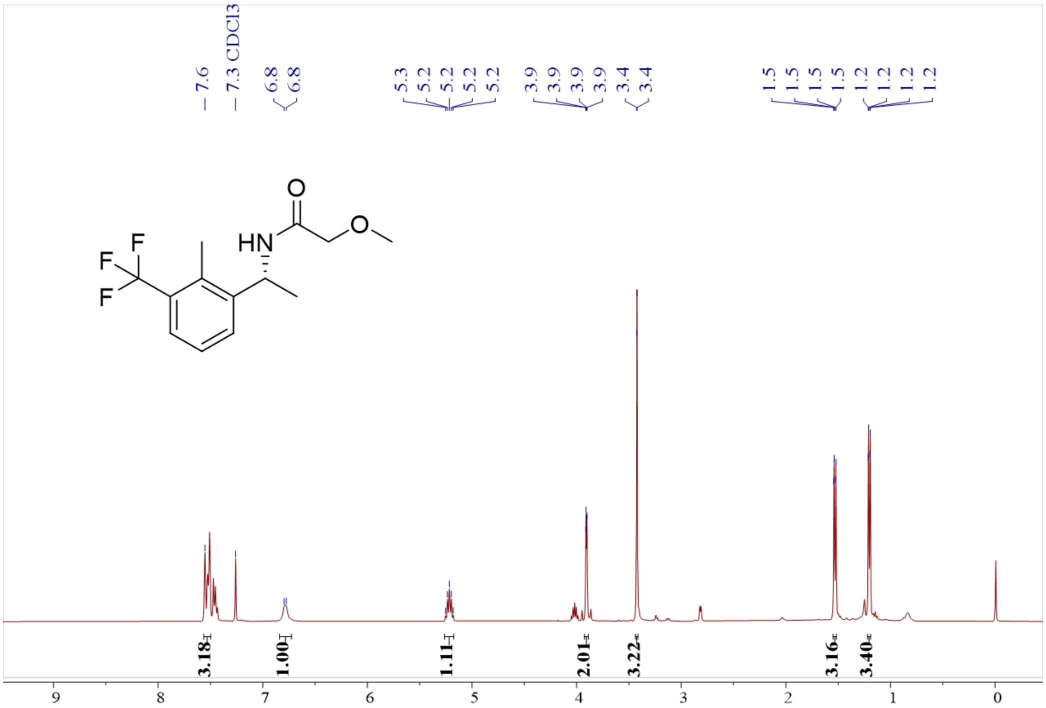
7.6
7.3 CDCl3
6.8
6.8
5.3
5.2
5.2
5.2
5.2
3.9
3.9
3.9
3.9
3.4
3.4
1.5
1.5
1.5
1.5
1.2
1.2
1.2
1.2
O
HN
O
F
F
F
3.18
1.00
1.11
2.01
3.22
3.16
3.40
9
8
7
6
5
4
3
2
1
0

7. Reference

[1] Q. Sun, C.-W. Fu, B. Aguila, J. Perman, S. Wang, H.-Y. Huang, F.-S. Xiao, S. Ma, *J. Am. Chem. Soc.* **2018**, *140* (3), 984.

[2] a) C. Bannwarth, E. Caldeweyher, S. Ehlert, A. Hansen, P. Pracht, J. Seibert, S. Spicher, S. Grimme, *Wiley Interdiscip. Rev.-Comput. Mol. Sci. Science* **2021**, *11* (2), e1493; b) P. Pracht, E. Caldeweyher, S. Ehlert, S. Grimme, **2019**.

[3] B. Stauch, S. J. Fisher, M. Cianci, *J. Lipid Res.* **2015**, *56* (12), 2348.

[4] C. Xing, P. Mei, Z. Mu, B. Li, X. Feng, Y. Zhang, B. Wang, *Angew. Chem., Int. Ed.* **2022**, *61* (21).
